# Effects of nursery production methods on fungal community diversity within soil and roots of *Abies alba* Mill.

**DOI:** 10.1038/s41598-023-48047-y

**Published:** 2023-12-02

**Authors:** Marlena Baranowska, Jolanta Behnke-Borowczyk, Władysław Barzdajn, Janusz Szmyt, Robert Korzeniewicz, Adrian Łukowski, Mirzeta Memišević-Hodžić, Natalia Kartawik, Wojciech Kowalkowski

**Affiliations:** 1 Faculty of Forestry and Wood Technology, Poznań University of Life Sciences, Wojska Polskiego 71a, 60-625 Poznan, Poland; 2 Faculty of Forestry, University of Sarajevo, Zagrebačka 20, 71000 Sarajevo, Bosnia and Herzegovina

**Keywords:** Plant immunity, Plant reproduction, Plant symbiosis, Environmental sciences

## Abstract

The aim of this study was to elucidate how different nursery production methods influence the composition of and relationship between soil and root community levels of *Abies alba*. In the Międzylesie Forest District, we quantified the responses of samples of both community-level fine roots and surrounding soil to environmental changes evoked by various seedling production methods. Fungi levels were identified based on their ITS 1 region and 5.8 S rDNA component. Analysis was conducted using Illumina SBS technology, and the obtained sequences were compared with reference samples deposited in the UNITE. Chemical analysis of the soil was also performed. Different nursery production methods resulted in a strong decoupling in the responses of fungal community levels between soil and roots. Changes in growth conditions imposed by production methods were significant in determining species composition. We found differences in fungal communities among functional groups of samples. In the soil, the dominant species of mycorrhizal fungi were *Tylospora asterophora*, *Amanita rubescens*, and *Russula ionochlora*. Mycorrhizal fungi in roots included *Tuber anniae, Thelephoraceae* sp., and *Acephala applanata*. Specific soil substrate conditions significantly influenced fungal community composition, leading to an increase in abundance of mycorrhizal fungi, specifically *T. anniae*.

## Introduction

Silver fir (*Abies alba* Mill.) is one of the most important forest tree species in the mountainous and upland regions of Poland, as well as in all important European alpine zones^1^. The presence of silver fir increases the biodiversity of forest ecosystems and enhances their resistance to wind, snow, and ice storms, making forest stands less susceptible to natural disturbances, such as fungal diseases and specialised insect herbivores^1^. Widespread introduction of spruce monocultures, deforestation, and a strong vulnerability to air and soil pollution have reduced areas occupied by this species and reduced not only distribution to new areas, but also spontaneous restoration. This restricted migration and limited genetic variability has made *A. alba* particularly sensitive to changes in climate. When taking ongoing climate change into account, these attributes could result in not only a stronger limit on range, but also a strong effect on natural regeneration and growth, making this species with its discrete provenances adapted to specific mountain conditions more prone to extinction. For this reason, the State Forests (Poland) introduced a programme of artificial restoration of valuable and unique fir resources in the Sudeten Mountains^2–10^.

Both biotic and abiotic factors strongly affect plant growth in forest stands, but even more significantly regulate seedling growth in the initial stages, even in forest nurseries^11,12^. High soil fertilisation and overwatering could play a large role in seedlings’ inability to adapt in order to overcome future water deficits, but can also positively result in a root and associated fungal community composition characterised by acquisitive ability within competitive mountain environments^13–15^. In view of the richness of mycocenoses, it is essential to determine the community of soil fungi accompanying a given tree species at the stage of nursery production, in order to ensure a controlled effect on its future composition and abundance in order to produce the best possible quality of planting material. The quality of forest nursery seedlings is affected by many elements, including production technology, site, seeding time, sowing rate, fertilisation, type of irrigation, and lighting^16^, but soil quality is one of the most important factors affecting plant growth. The physico-chemical properties of soils modify the community structure of soil fungi and bacteria^17,18^. Furthermore, a growing body of evidence shows that there is a relationship between crop fertilisation and fungal community character, as well as indicating that the full range of their complexity may also result from aboveground resource accessibility^19,20^. We therefore assumed that variations in seedling production may interact with fungal community and with light conditions (different conditions under the forest canopy and in open field nurseries), thereby affecting plant growth and resistance to pathogens via the shaping of fungal communities. For example, Menkis et al.^21^ found moderate similarity in mycorrhizal communities between pine and spruce, and among different cultivation systems. Photosynthesis, driving the amount of carbohydrates translocated to the root system, can shape not only root acquisitive traits, but also microbial composition and acquisitive potential^22,23^. Thus, the intensity of fertilisation alone is not predictive of the composition of root fungal communities. Although soil fertilisation during seedling production in nurseries has been recognised as a crucial modulator of seedlings’ future growth^24^, the effect of interactions between nutrient abundance and light availability on fungal community composition remains poorly understand due to difficulties in assessing all these variables. To our knowledge, the possible ways in which technologies associated with silver fir production can modify fungal composition have received little, if any, attention.

**Figure 1:**
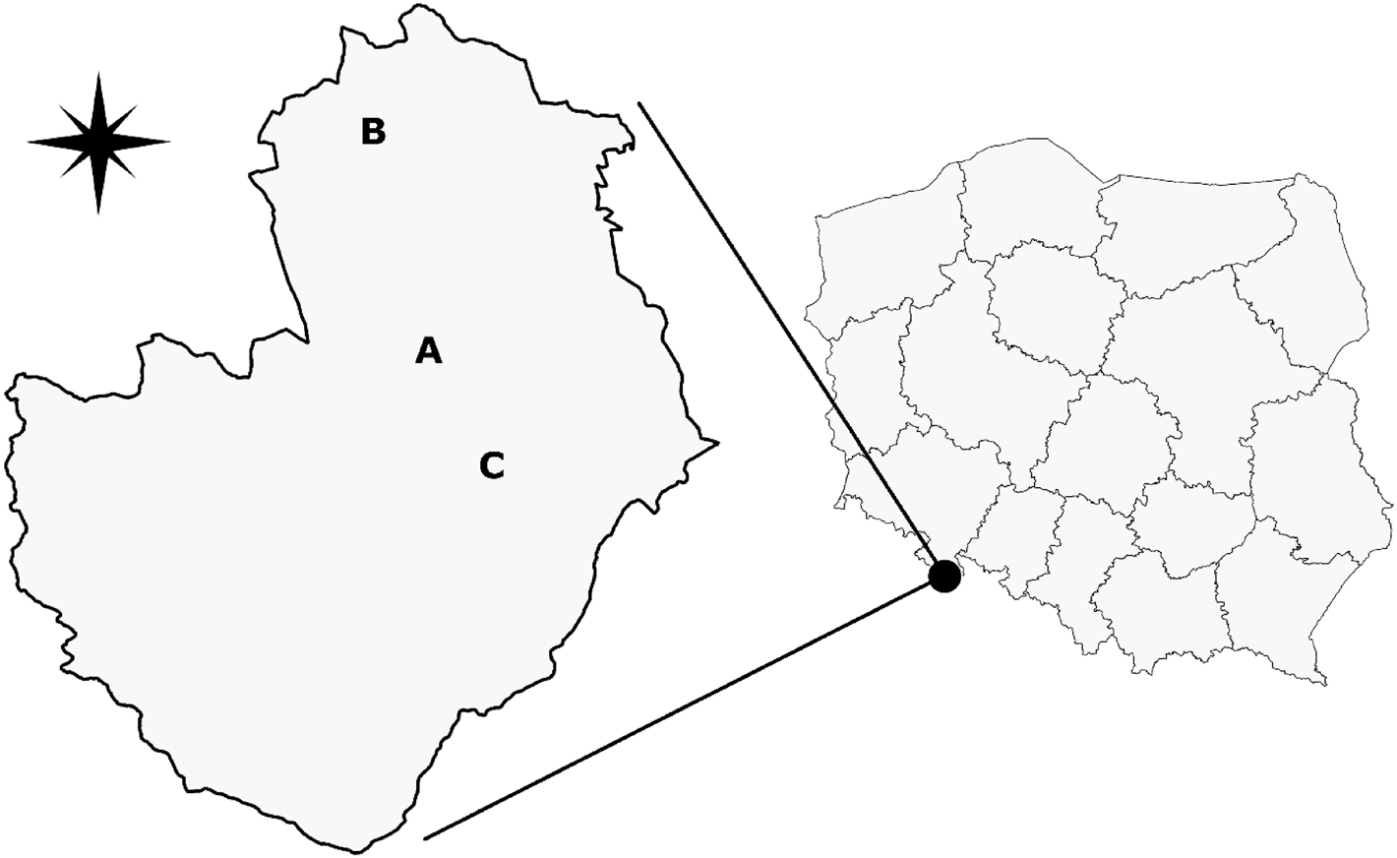
Location of the study site in the Międzylesie Forest District of the National State Forest, Poland, and location of specific forest nurseries within the Międzylesie Forest District. A—field and container nurseries, B—nursery under Norway spruce canopy, C—nursery under Scots pine canopy and natural regeneration in a silver fir stand.

This study aimed to elucidate how fungal community diversity is affected by varied techniques of fir seedling production, as well assessing if their functional diversity is affected by alternation in resource availability (i.e. nutrients and light). To achieve these goals, we investigated the fungal community around fine roots and collected samples of soil from under fir seedlings being grown via one of six different production techniques: (I) an open field nursery, where 3-year-old fir seedlings were produced, (II) an open field nursery, where 3-year-old fir seedlings that had been transplanted (to reduce their density) after their second year of production, (III) a containers nursery, (IV) a nursery under a Scots pine canopy, (V) a nursery under a Norway spruce canopy, and (VI) natural regeneration in a silver fir stand. We assumed that mycorrhizal fungi and saprotrophs would represent a dominant portion of such communities. The following research question was posed: Will the communities of soil fungi and fine roots of silver fir differ depending on the method of nursery production? This research shed light on how the ecology of fungal communities around fir seedlings can be shaped by the influence of the soil properties of different forest nurseries (Fig. 1).

## Results

### Chemical composition of soil

Analysis of the chemical traits of soil samples showed that nursery sites were characterised by a high nitrogen concentration that generally exceeded optimal values^25^. Soil sampled from under seedlings growing in natural or quasi-natural conditions (nurseries under shelterwood) exhibited lower levels of nitrogen than in the nurseries in open fields or in peat substrate (Table 1).

**Table 1:** Concentrations of macronutrients, pH levels, and organic matter content calculated as an average of the five soil samples taken from each treatment.

Parameters^1^	Treatments					
	IS	IIS	IIIS	IVS	VS	VIS
	SP30	SP21	KOST	SO	SW	NAT
N-NO_3_ [mg/100 g soil]	36.91	44.47	170.26	67.52	135.60	6.42
N-NH_4_ [mg/100 g soil]	10.86	7.75	18.78	10.09	22.32	48.53
Total N [%]	0.19	0.24	0.63	0.45	0.33	0.22
P_2_O_5_ [mg/100 g soil]	11.29	10.79	20.69	2.84	6.19	3.55
K_2_O [mg/100 g soil]	18.18	12.88	41.66	8.25	13.48	17.10
MgO [mg/100 g soil]	22.38	30.01	63.83	16.26	7.78	12.50
K:Mg	1.12	0.59	0.90	0.70	2.39	1.88
C [% d.m.]	2.60	3.21	13.95	10.26	5.62	3.73
C:N	14	17	23	13	17	22
pH_KCl_	5.1	3.5	3.1	4.4	3.4	5.3

More information regarding individual treatments and sampling can be found in “Materials and methods” and “Molecular identification of fungal communities” sections.

^1^Optimal values: 20 mg/100 g soil of N-NO_3_ and NNH_4_ together, 18 mg/100 g of K_2_O, 5 mg/100 g of P_2_O_5_, and 7 mg/100 g of MgO^25^.

### Molecular identification of fungal communities

**Figure 2:**
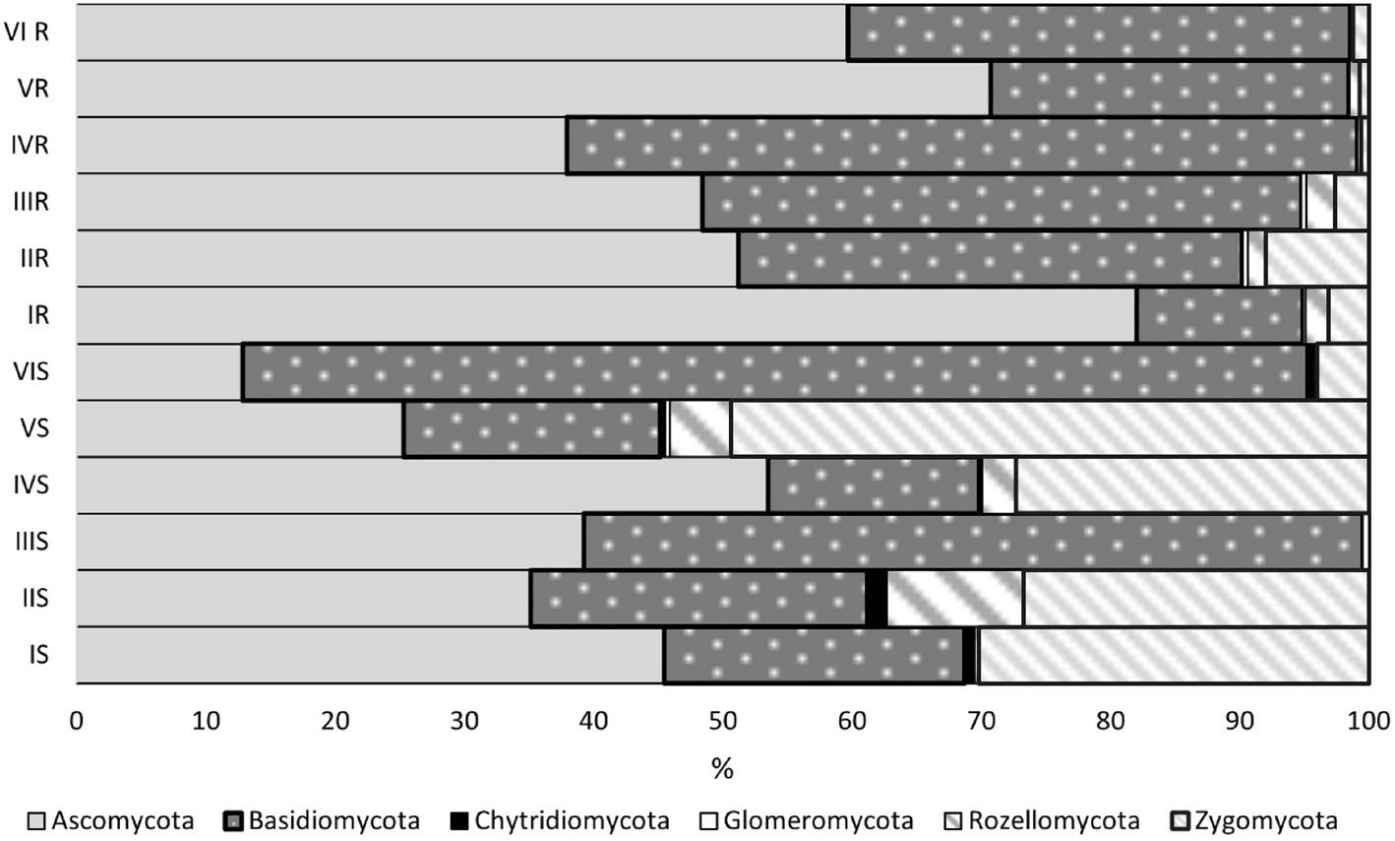
Share [%] of phyla in fungal communities around fine roots and in soil represented. More information regarding individual treatments can be found in “Materials and methods” and “Molecular identification of fungal communities” sections.

A total of 742,903 OTUs were obtained, 77.26% of which belonged to fungi. The identified fungal taxa mainly represented six phyla: Ascomycota, which comprised 9.174–60.41% of each treatment–site community (Fig. 2); Basidiomycota, which comprised 7.706–72.591% of each community; Zygomycota, which comprised 0–0.63% of each community; Glomeromycota, which comprised 0.131–2.399% of each community; Rozellomycota, which comprised 0.096–2.768% of each community; and Chytridiomycota, which comprised 0–0.352% of each community.

**Figure 3:**
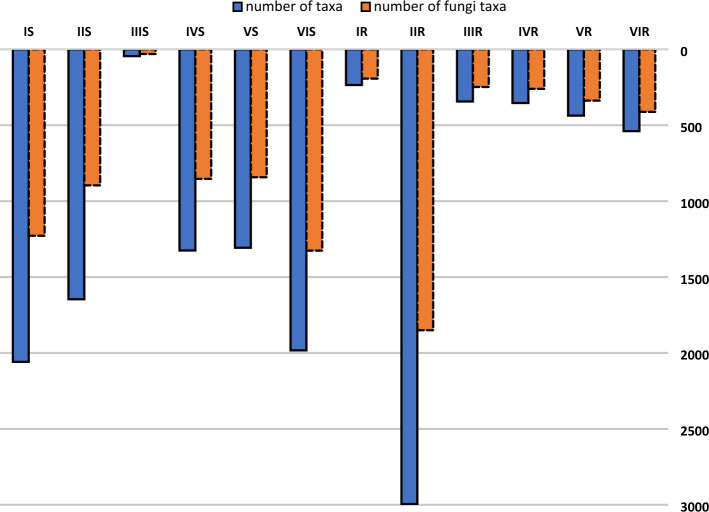
The total number of taxa (blue) identified in soil samples and the number of fungal taxa (orange) identified in soil samples. More information regarding individual treatments can be found in “Materials and methods” and “Molecular identification of fungal communities” sections.

Overall, 13,269 taxa were identified, including 8479 fungal taxa. Fine root samples from the IIR treatment (3-year-old silver fir seedlings transplanted after their second year of growth in an open field nursery) contained the greatest number of fungal taxa, and the fewest fungal taxa were found in soil samples from the IIIS treatment (nursery production in containers; Fig. 3). Detailed results of the identification of fungi, along with their function in the community, are included in Supplementary Appendix 2.

**Figure 4:**
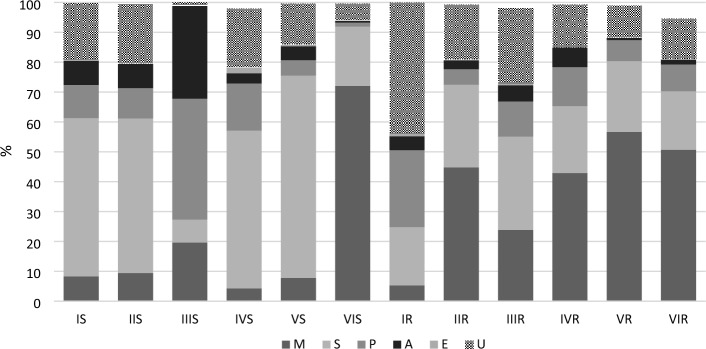
Share [%] of fungal OTUs belonging to individual functional groups: mycorrhizae (M), saprotrophs (S), pathogens (P), antagonists (A), entomopathogens (E), unknown (U). More information regarding individual treatments can be found in “Materials and methods” and “Molecular identification of fungal communities” sections.

The majority of the soil fungal community consisted of saprotrophic fungi, mycorrhizal fungi, and those for which a function could not be identified (Fig. 4). Although we expected to observe few mycorrhizal fungi within under-canopy nursery treatments, we observed similar mycorrhizal fungi proportions in both the open field nursery and the nursery under a Scots pine canopy, as well as a consistent pattern of occurrence of mycorrhizal fungi among soil and fine roots of fir seedlings growing under a Norway spruce canopy, and a shift in community composition between soil and fine roots in the container nursery. Specifically, we observed a rise in the abundance of mycorrhizal fungi colonising fine roots in relation to soil chemical composition, and an opposite trend was observed for pathogens and antagonists. In addition, pathogens and antagonists of forest tree pathogens, as well as limited numbers of entomopathogenic fungi, were also detected (Fig. 4).

### Results of statistical and bioinformatic analysis

The highest value for Margalef’s index, which defined relative species richness in relation to the total number of species and total number of all specimens in a given community, was recorded for the fungal community present in fine roots in the IIR treatment (3-year-old silver fir seedlings transplanted after their second year of growth in an open field nursery; Table 2). In turn, the lowest community abundance was found in the soil of the IIIS treatment (nursery production in containers with a peat bed). The fine roots of silver fir were characterised by a higher Shannon’s diversity index than that of the fungal community present in the soil, reaching a maximum in fine roots in the IR treatment (3-year-old silver fir seedlings grown in an open field nursery) and a minimum in soil in the IIIS treatment. Evenness, which describes the proportions of individual species in the community and is defined by Shannon’s evenness index, was highest for the fungal community in soil from the IR treatment and lowest in soil from the IIIS treatment. As in the case of Shannon’s diversity index, higher values for Shannon’s evenness index were obtained for the fungal community of fine roots.

**Table 2:** Biodiversity of fungal communities determined based on the biodiversity indices in individual variants of the experiment.

Index	Treatments											
	Soil						Root system					
	IS	IIS	IIIS	IVS	VS	VIS	IR	IIR	IIIR	IVR	VR	VIR
Index D-Mg	111.44	87.24	3.07	77.83	76.15	115.01	19.43	166.77	24.17	24.14	31.83	38.24
Shannon’s diversity index H	2.98	2.66	1.22	3.50	3.13	3.28	3.80	3.78	3.70	2.91	2.83	3.41
Shannon’s evenness index E	0.42	0.39	0.35	0.52	0.47	0.46	0.72	0.50	0.67	0.52	0.49	0.57
Simpson’s Index	0.22	0.27	0.49	0.09	0.13	0.10	0.05	0.06	0.07	0.14	0.15	0.06
Berger–Parker dominance	0.07	0.05	0.10	0.08	0.11	0.28	0.17	0.12	0.06	0.34	0.35	0.15

More information regarding individual treatments can be found in “Materials and methods” and “Molecular identification of fungal communities” sections.

Simpson’s index, which expresses the probability of finding two specimens belonging to the same species in a random sample, was highest for the fungal community in soil from the IIIS treatment and lowest in fine root samples from the IR treatment. Lower values of this index were obtained for the fungal community of fine roots than for that of soil. The highest values of the dominance index were obtained for the fungal community in roots from the VR treatment (nursery under a Norway spruce canopy), whereas the lowest values were found in soil from the IIS treatment. The analysis of variance resulted in χ^2^ = 131,265 for the structure of functional groups of fungi on roots, and χ^2^ = 33,833 for fungi on roots. The critical value of this statistic for the significance level p = 0.01 was χ^2^ = 59.70, indicating that the structure of the numbers of individual functional groups varied greatly, both in the case of soil fungi and fungi isolated from roots.

It was assumed that the numbers of OTUs in individual groups obtained from soil and roots would correlate, as the same fungi should be found on roots as in the soil in which the roots were growing. A comparison of the numbers of OTUs in individual taxa from soil and from roots provided a Pearson’s correlation coefficient of r = 0.4883 (p = 0.01), confirming this correlation, although it was far from fully consistent and defined as moderate.

Linear correlation coefficients between quantitatively determined properties of soil and nursery container substrate, and the numbers of isolated fungi belonging to identified functional groups are presented in Table 3. The presence of mycorrhizal fungi in soil was related to a high ammonia nitrogen content and high acidity, but the presence of these fungi on roots was not dependent on soil nutrient abundance. High levels of phosphorus and magnesium did not promote the presence of saprotrophic fungi in soil.

**Table 3:** Linear correlation coefficients between numbers of fungal OTUs from soil and roots, and soil properties such as nutrient concentrations, organic carbon content, and acidity.

Soil property	Mycorrhizal	Saprotrophs	Pathogens	Antagonists	Entomophagous	Unidentified
Soil						
N-NO_3_	− 0.5491	− 0.2373	− 0.0096	0.3499	− 0.1253	− 0.4907
N-NH_4_	**0.9338**	0.2161	− 0.4681	− 0.5425	− 0.1071	0.0076
Total N	**0.8250**	− *0.0849*	− 0.4876	− 0.5870	− 0.1805	− 0.2792
P_2_O_5_	− 0.4324	− **0.8256**	− 0.2982	0.3652	− 0.6803	− **0.8463**
K_2_O	− 0.0746	− 0.7170	− 0.3139	0.1742	− 0.5616	− 0.7987
MgO	− 0.3341	− **0.9151**	− 0.1630	0.1148	− 0.4722	− **0.8460**
K:Mg	0.4444	0.6493	− 0.3790	− 0.0002	− 0.0817	0.2304
C%	0.7856	− 0.2070	− 0.5363	− 0.5700	− 0.2737	− 0.3894
C:N	0.5074	− 0.5109	− 0.7049	− 0.4502	− 0.5575	− 0.7362
pH_KCl_	**0.8487**	0.5590	− 0.1364	− 0.5917	0.3346	0.3972
Roots						
N-NO_3_	− 0.2953	− 0.1884	− 0.5259	− 0.1507	− 0.2183	− 0.6060
N-NH_4_	0.2618	− 0.1541	− 0.1267	− 0.4775	− 0.3507	− 0.4486
Total N	0.1671	− 0.1290	− 0.2593	− 0.3858	− 0.3202	− 0.4824
P_2_O_5_	− 0.6647	− 0.2945	− 0.6051	− 0.2341	0.6106	0.0198
K_2_O	− 0.6317	− 0.4579	− 0.5466	− 0.3182	0.3423	− *0.3235*
MgO	− 0.5345	− 0.1501	− 0.4386	0.0462	0.4523	0.0435
K:Mg	0.2065	− 0.2643	− 0.2944	− 0.7313	− 0.4688	− 0.6928
C%	0.1030	− 0.1358	− 0.3264	− 0.3983	− 0.2229	− 0.4334
C:N	− 0.0189	− 0.0721	− 0.6421	− 0.4734	− 0.0112	− 0.3376
pH_KCl_	0.5342	0.0445	0.1993	− 0.1753	− 0.6735	− 0.4441

Correlation coefficients, considered significant at p = 0.05, are given in bold.

Significant values are in italics.

### Analysis of similarities and fungal indicator species

#### Similarities among the most abundant fungal species

**Figure 5:**
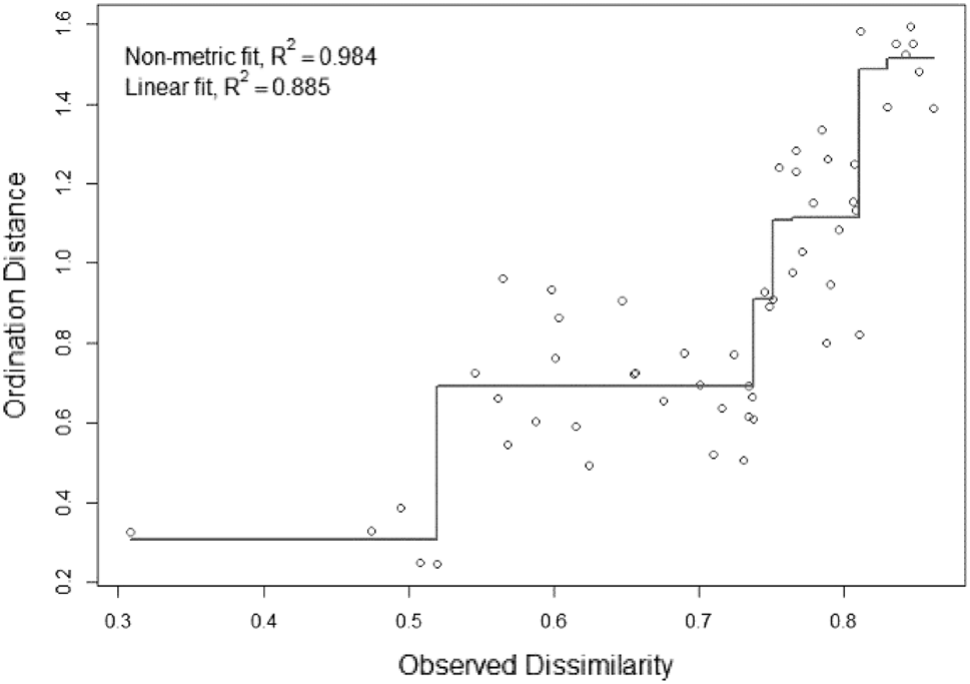
Shepard diagram displaying statistics for goodness of fit between ordination distances and observed dissimilarity. Only the most abundant fungal species were included (share > 1%).

A Shepard diagram indicated scatter around the regression between the inter-point distances in the final configuration (between each pair of species communities) and their original dissimilarities (Fig. 5), suggesting that original dissimilarities are well preserved in the reduced number of dimensions in the final configuration.

**Figure 6:**
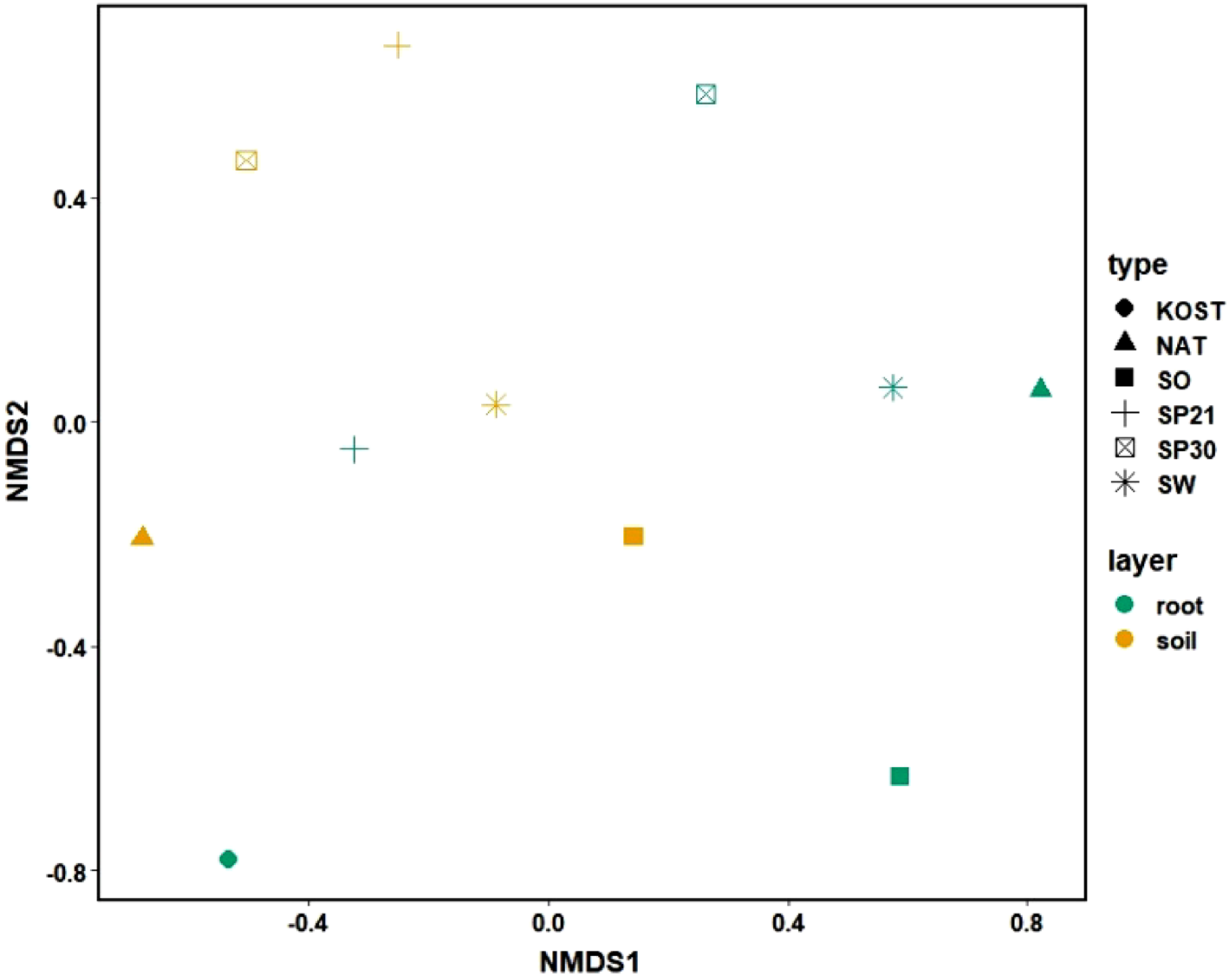
A Bray–Curtis plot of non-metric multidimensional scaling for the most abundant fungal species. Colours indicate the values for fine root samples and for soil; symbols indicate specific methods of silver fir seedling production in the nursery. More information regarding individual treatments can be found in “Materials and methods” and “Molecular identification of fungal communities” sections.

Results of the ANOSIM showed that there was a statistically significant difference (R = 0.34, p = 0.02) between fungal communities around fine roots and those in soil. In the case of nursery production, the NMDS indicated that a lack of difference between fungal species could not be rejected (R = 0.04, p = 0.42; Fig. 6).

#### Analysis of indicator species

The indicator value index showed that, of the 39 fungal species analysed, two could be identified as diagnostic species for nursery production (Table 4). Additionally, results showed that *Tuber anniae* was a good indicator species of root community because it occurred only in root samples. *Rhizoscyphus* spp. were present in all treatments and were largely restricted to the fungal community of fine roots. In the case of the fungal community in soil, five fungal species were identified as indicator species, appearing at all sites belonging to this community (Table 4). *Mortierella horticola*, *Amanita rubescens*, *M. macrocystis*, *Solicoccozyma terrea*, and *M. exiqua* were considered to be good indicators because their Iv values were all very close to 1, indicating their strong association with soil.

**Table 4:** Indicator value index (Iv) calculated for the most abundant fungal species and their association to specific fungal communities (root or soil).

Kind of fungal communities	Species	Iv index	A component	B component	p-value
Root	*Rhizoscyphus*	0.994	0.9883	1.0000	0.0064
	*T. anniae*	0.913	1.0000	0.8333	0.0139
Soil	*M. horticola*	0.988	0.9770	1.0000	0.0087
	*A. rubescens*	0.982	0.9634	1.0000	0.0118
	*M. macrocystis*	0.974	0.9487	1.0000	0.0079
	*S. terrea*	0.972	0.9450	1.0000	0.0080
	*M. exigua*	0.964	0.9285	1.0000	0.0293

Species are presented beginning with the strongest association at the top of the table.

A total of 51 species of fungi were identified, but only one species, *Tuber anniae*, could be identified as a diagnostic species, with its occurrence restricted to soil (Iv = 1.000, p = 0.02).

#### Species of mycorrhizal fungi

**Figure 7:**
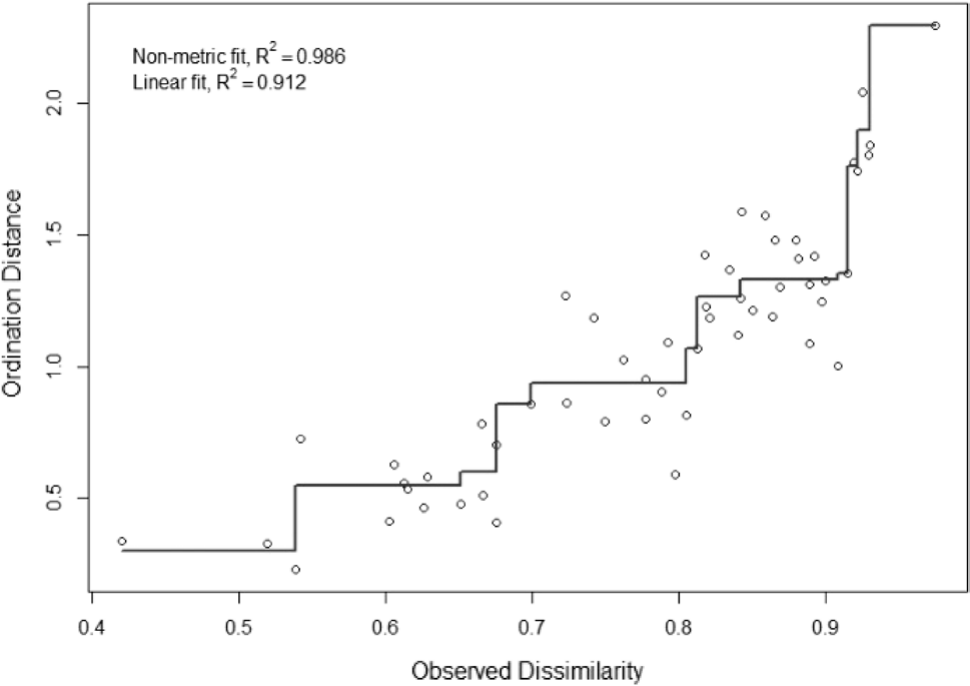
Shepard diagram displaying statistics for goodness of fit between ordination distances and observed dissimilarity for communities of mycorrhizal fungi only. Only the most abundant fungal species were included (share > 1%).

A Shepard diagram computed for species of mycorrhizal fungi suggested that original dissimilarities were well preserved in the reduced number of dimensions in the final configuration (Fig. 7).

**Figure 8:**
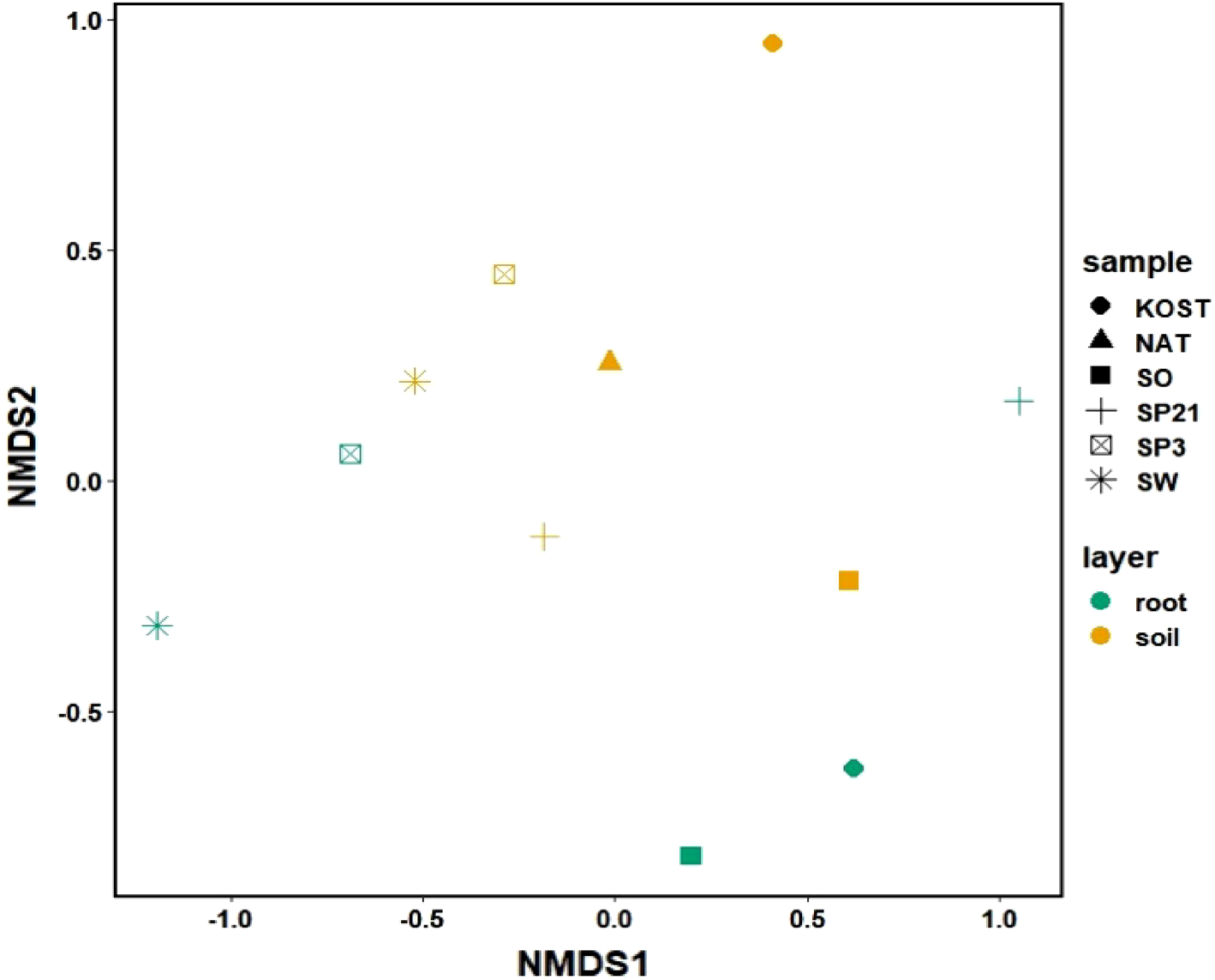
A Bray–Curtis plot of non-metric multidimensional scaling of the species diversity of mycorrhizal fungi. Colours indicate the values for fine roots and for soil (place of sampling); symbols indicate specific methods of silver fir seedling production in the nursery. More information regarding individual treatments can be found in “Materials and methods” and “Molecular identification of fungal communities” sections.

The NMDS for mycorrhizal fungi revealed that samples were distinct based on place of sampling (root or soil; Fig. 8). The closer two points (samples) were on the graph, the more similar those points were considered to be.

The ANOSIM statistic indicated a significant difference between species of mycorrhizal fungi based on place of sampling (R = 0.23, p = 0.07), but no difference among methods of silver fir seedling production in the nursery (R = 0.0, p = 0.61). Thus, the null hypothesis stating a lack of difference between species of mycorrhizal fungi could only be rejected in the case of place of sampling.

## Discussion

In this study, we found that the composition of soil fungal community depended on method of seedling production and was related to the soil conditions of cultivation sites. We also observed that soil and root fungal communities varied in functional group dominance—mycorrhizal fungi dominated in fine roots, whereas saprotrophs were primarily present in soil substrate.

Our investigation showed that members of Zygomycota represented the majority of saprotrophs, particularly in soil communities. Mycorrhizal fungi, however, represented the majority of taxa commonly found in boreal forests and in the temperate zone of Europe (both in the soil and in fine root samples). In the fungal communities we tested, members of the genera *Amphinema, Clavulina, Lactarius, Piloderma, Tuber, Boletus*, and *Cenococcum* were detected, similar to the report of Eberhardt et al.^26^. We also found species belonging to the genera *Inocybe* and *Sebacina*, as did Ishida et al.^27^ and Kranabetter et al.^28^. Tests of soil associated with *Abies religiosa* that were conducted using Illumina MiSeq to sequence the ITS2 region identified 1746 taxa, with the greatest share of fungi belonging to the genera *Russula*, *Mortierella*, and *Piloderma*, and with Russulaceae and Clavulinaceae being the dominant families of mycorrhizal fungi present^29^.

In our study, the extent to which the community of mycorrhizal fungi inhabiting the soil were also present in the roots indicated that, generally, the community composition of mycorrhizal fungi was not driven by the mycorrhizal conditions in the nurseries, but may instead have been a feature of whether or not fir micro-site conditions were suitable for the growth of specific mycorrhizal species. This is consistent with previous studies that have identified the same groups of fungi as being associated with soil and fir roots. Ważny and Kowalski^30^ also identified *Piloderma* sp., *Tylospora asterophora*, and *Russula integra* in 1-year-old firs in a natural regeneration setting. Reports of *Tylospora* accounting for a considerable proportion of fungi in soil communities have previously confirmed that this genus forms mycorrhizae with young trees^31^. A study of fungi associated with the fine roots of firs that were more than 35 years old identified the genera *Elaphomyces*, *Lactarius*, and *Russula* as comprising a majority of the community^32^. Similar to the findings of Ważny^33^, mycorrhizal fungi in the community analysed in the current study included *Clavulina cristata*, *Tomentella* sp., *Tuber puberulum*, and *Clavulina* sp. Additionally, the family Thelephoraceae was a dominant taxon of mycorrhizal fungi detected in the samples tested in our research. The DEEMY database showed that 13 fungal species formed ectomycorrhizae with the genus *Abies*—fungi belonging to the genera *Russula*, *Tricholoma*, *Lactarius*, and *Cortinarius*. As Rudawska et al.^34^ reported when examining the roots of firs found outside their typical range limits, our study of fungal communities also confirmed the presence of *Cenococcum geophilum*, *Xerocomellus pruinatus*, *Tylospora asterophora*, *Amanita muscaria*, and *Laccaria amethystina*.

Our analysis showed that *Rhizoscyphus* spp. were indicator species of the fungal community associated with roots. Indeed, *Rhizoscyphus ericae* (*Hymenoscyphus ericae*) is an ecologically important species complex that includes fungi living in symbiotic relationships with plant roots as either endophytes or mycorrhizal symbionts of plants in the family Ericaceae, and as ectomycorrhizal partners of plants in the families Betulaceae, Fagaceae, Pinaceae and Salicaceae^35^.

Greater fertilisation of the soil affected fungal composition, suggesting that fir saplings invested less in their association with mycorrhizal fungi when nutrients were more abundant, although fertilisation appeared to affect the ways in which mycorrhizal fungi foraged in the surrounding soil. In our study, we showed that natural conditions were characterised by the presence of *Russula*, which possesses a greater ability to regenerate in response to environmental alteration^36^. Although a greater abundance of short- or medium-distance mycorrhizal types may mirror growth conditions of forest nurseries in the early stages of seedling growth, short distance exploration may not provide enough water to fulfil the water demands of older trees. In the case of naturally regenerated fir, long taproots may not enhance foraging in deep soil layers. Furthermore, although fungal composition and richness may be related to canopy conditions, we observed in our study that the greatest richness was present within the IIR (SP21) treatment, as a result of a great abundance of unique species. In fine root samples from the IIR treatment, the following species represented the majority: *Tuber anniae*, *Inocybe rufoalba*, and *Tylospora asterophora*. Our results showed that *T. anniae* was a good indicator species of the root community because it only occurred in that community. In the case of soil community, *Amanita rubescens* was identified as an indicator species, as it appeared in soil at all sites.

Growth conditions and belowground carbon flux is strongly related to light availability and disturbance^37^. We found that even in a closed canopy nursery under Scots pine (IVR), fir seedlings received an energy flux similar to that experienced by seedlings in an open canopy nursery, based on abundance of mycorrhizal fungi. In the root community from the IVR treatment, *Acephala applanata* was the dominant mycorrhizal fungi. A significant portion of the community of fine-root fungi also consisted of this species in the natural regeneration treatment (VIR). Dark septate endophytes of the *Phialocephala fortinii* s.l.–*A. applanata* species complex (PAC) are presumed to be the most abundant root-colonising endophytes of conifers across the Northern hemisphere^38^. Some PAC strains reduce the growth rate of their hosts, but are beneficial in protecting roots against pathogens. Nothing is known about the effects of PAC on mycorrhizal fungi or how the PAC–mycorrhiza association affects plant growth, even though these two fungal groups occur in close proximity in natural habitats^39^. Reininger and Sieber^39^ suggested that global warming could cause a general decrease of mycorrhization, making primary roots more accessible to other symbionts and pathogens. As the role of PAC in fir-associated fungal communities has not yet been recognised, dark septate endophytes in the fir rhizosphere/fungal community require further research.

Typically, increased carbon fluxes to roots and soil are expected to support fungal richness^40^. Contrary to our expectations, however, we observed that in the IIIR and IIIS treatments, the biodiversity of the fungal community did not correspond to light availability or potential changes in carbohydrate availability. Indeed, we observed the smallest fraction of mycorrhizal fungi in the fungal community of substrate (IIIS) after fir seedling production in the container nursery (Kosterkiewicz’s system^10^). This suggests that the composition of fungal communities depends on the type of seedling production. A study by Banach et al.^17^ showed that the growth rates of 1- and 2-year-old containerised silver fir seedlings varied depending on the applied substrate. The application of mycorrhizal inoculum (*Hebeloma crustuliniforme* has been found to have a positive effect on height growth in fir seedlings^17^, which may be explained by variation in the responses of roots to the changes in growth conditions experienced by containerised seedlings, in comparison to those faced by seedlings in open and canopied nurseries. Lower levels of colonisation by mycorrhizal fungi is generally attributed to high fertilisation or shaded growth conditions, which can both reduce the engagement of mycorrhizal fungi in resource acquisition and the availability of carbohydrates. It is not yet apparent, however, if application of high fertilisation in a container in the early stages of development reduces the ability of fir seedlings to establish mycorrhizal symbiosis at later stages of growth in the field. Methods of fir production that prioritise allocation of resources to leaves to maximise carbon gain, in response to fertilisation, could alter the composition of soil fungi, however, which could in turn threaten survival by reducing the probability of establishing beneficial mycorrhizal symbiosis. The relatively low proportion of common species in the mycorrhizal community of containerised fir seedlings may be an obstacle to their further survival, and highlights the requirement for artificial manipulation of the growth substrate by application of mycorrhizal inoculum. The local conditions of the site likely did not allow for switches and adjustments of fungal partners to new conditions, or limited the abundance of ectomycorrhizal fungi in the soil community.

Our investigation showed that greater phosphorus and magnesium content in the soil was associated with fewer saprotrophs and fungi of unspecified function in the community. In contrast, greater nitrogen content was associated with a greater proportion of the soil community consisting of mycorrhizal fungi. The diversity and activity of soil fungi are regulated by various factors, both biotic and abiotic^41,42^. In our study, the greatest diversity in the community of soil fungi was observed in samples from the natural regeneration site for fir, whereas the greatest diversity in the community of root fungi was found in samples from the 3-year-old (2/1) fir seedlings transplanted after their second year of production.

### Conclusion

The abundance of mycorrhizal fungi and low number of pathogens in the community of soil fungi from forest nurseries producing fir seedlings is a good sign, indicating the quality of the nursery environment. We found that similarities in the taxonomic composition and the population size of fungi in soil and on seedling roots existed and were demonstrable, but they were only moderate. The relationship between the taxonomic composition and abundance of fungi, and soil properties remains far from evident, although our study confirmed a positive effect of soil ammonia nitrogen content and acidity on mycorrhizal fungi, as well as a similarly negative effect of phosphorus and magnesium content on saprotrophic fungi.

Identification of soil fungal communities from nurseries producing silver fir seedlings made it possible to determine the spectrum of mycorrhizal fungi characteristic of this tree species at a given age (3 years). *Tuber anniae* was a good indicator species of root group because it occurred only in root samples. *Rhizoscyphus* spp. could also be indicators, as they were present in roots at all sites and were largely restricted to root samples. Finally, fungal presence was very low in the container method of production treatment (peat substrate), indicating that artificial mycorrhization could be beneficial for the sustainable development of seedlings via this production method.

## Materials and methods

### Location

The experiment was conducted in the Międzylesie Forest District, located in the Central Sudeten Mountains in the Śnieżnik massif, the Bystrzyckie Mountains, and the Upper Nysa Graben in the south of Poland (Fig. 1). A detailed description of nurseries is included in Supplementary Appendix 1.

### Treatments and sampling

Samples (denoted as S) were collected in June 2017. Samples from Treatment I, labelled SP30, were collected from soil at a nursery in an open field where 3-year-old (3/0—production symbol used in Polish nurseries) fir seedlings were produced. Samples from Treatment II, labelled SP21, were collected from the same location, but from soil under 3-year-old (2/1—production symbol commonly used in Polish nurseries) fir seedlings that had been transplanted (to reduce their density) after their second year of production. Samples from Treatment III, labelled KOST, consisted of peat substrate derived from nursery containers comprising a system based on J. Kosterkiewicz’s method^10^. Samples from Treatment IV, labelled SO, were collected from soil in a nursery under a Scots pine canopy, and samples from Treatment V, labelled SW, were collected from a nursery under a Norway spruce canopy. Fir seedling production methods carried out at studied nurseries are described by Robakowski et al.^10^. Finally, samples from Treatment VI, labelled NAT, were collected from forest soil in a mature fir stand with natural regeneration. The plant collection and use was in accordance with all the relevant guidelines and legislation. Permissions were obtained from the nursery owners for collection of samples.

In total, 30 soil samples (five samples per each treatment: IS, IIS, IIIS, IVS, VS, VIS) and 30 fine-root samples from 3-year-old seedlings (five samples per each treatment: IR, IIR, IIIR, IVR, VR, VIR) were harvested. Samples were collected from soil at a maximum depth of 25 cm, and each sample collection site was 20 m apart. Individual soil samples were placed in a container and then thoroughly mixed to create a representative sample (approx. 0.5 kg) that was used for further tests. Samples of fine roots were collected from the same plots (nested in treatments) as those from which soil samples were taken. Root samples were packed into paper envelopes, and soil samples were packed into plastic bags. Each sample was packed separately and immediately taken to the laboratory, where seedlings were removed from the ground, adhering soil was removed with water, and then root branches within the exposed root system were randomly selected. Comparably sized subsamples of fine root branches (2 cm in length) were placed in 2-ml screw cap tubes.

### Soil chemical analysis

The soil samples were analysed by the Seed Testing Station of the National Forests Research and Implementation Centre of State Forest in Bedoń, Poland. The analyses included measurements of:

- pH in KCl using an electrochemical technique, as well as phosphorus and potassium content according to Egner–Rhiem via inductively coupled plasma atomic emission spectrometry^9,10^.
- Magnesium content according to Schachtschabel via inductively coupled plasma atomic emission spectrometry^43^.
- Nitrogen content via a direct method using the TruSpec CHNS apparatus, as well as organic carbon content via the modified Tiurin method^44^.
- N-NO_3_ and N-NH_4_ via an electrochemical method following extraction in 0.03 N acetic acid^44^.

### Molecular identification of the fungal community

Root samples were washed on sieves under running water and dried in sterile blotting paper. After drying, the roots were ground in a mortar and frozen to − 70 °C. DNA was then extracted using the Plant Genomic DNA Purification Kit (Thermo Scientific), following the manufacturer’s protocol. DNA extraction from soil was performed using the DNeasy PowerSoil Kit (QIAGEN, Hilden, Germany), according to the manufacturer’s protocol. The DNA was then purified using the Anti-Inhibitor Kit (A&A Biotechnology, Gdynia, Poland). Fungi were identified based on their internal transcribed spacer (ITS) 1 region and their 5.8 S rDNA component. The analysis was performed using the following primers: ITS1FI2 5′-GAACCWGCGGARGGATCA-3′^45^, and 5.8S 5′-CGC TGCGTT CTTCATCG-3′^46^. The reaction mixture consisted of 2.5 µl DNA, 0.2 µl of each primer, and 12.5 µl 2× PCR MIX (A&A Biotechnology), with deionised water added to reach a final volume of 25 µl. Amplification was run in a thermocycler with a cycle consisting of preliminary denaturation (94 °C, 5 min), 35 denaturation cycles (94 °C, 30 s each), annealing (56 °C, 30 s), elongation (72 °C, 30 s), and final elongation (72 °C, 7 min). Next, the product was visualised on 1% agarose gel, using Midori Green Advance DNA (Genetics) for staining. The obtained product was purified and sequenced using SBS technology by Illumina (Genomed S.A., Warszawa). Sequencing was performed on a MiSeq sequencer in paired-end (PE) technology. Negative samples (without DNA) were also sequenced to remove artifacts.

The raw data used in the compilation of the results can be found in Supplementary Appendix 3 and 10.6084/m9.figshare.23404070.v1.

### Statistical and bioinformatic analysis

Results of the fungal DNA isolation were subjected to statistical analysis, as described in detail by Behnke-Borowczyk et al.^47^. A table of operational taxonomic units (OTUs) was prepared by PIPITS, version 1.2.0^48^. Read pairs were joined with PEAR (version 0.9.6^49^, quality‐filtered with a quality threshold of q = 30 by the FASTX–Toolkit (version 0.0.13), converted to Fasta format, and then merged into a single file. Prepared sequences were dereplicated, and subregions of the ITS region were selected with the use of ITSx (version 1.0.11^50^). Unique sequences and those shorter than 100 bp were removed from further analysis. Sequences were compared by applying the BLAST algorithm with reference sequences from the Communication and Identification of DNA database (UNITE community database https://unite.ut.ee/)^51^. For identification, the percentage of sequence similarity with the reference sequence was assumed to be 98–100%, with a minimum coverage of 90%. The resulting representative sequences for each cluster were subjected to chimera detection and removal using the UNITE UCHIME reference data set (version 6.0; https://unite.ut.ee/index.php). The input sequences were then mapped onto the representative sequences, and taxonomy was assigned with the use of the RDP Classifier (version 2.10.2^52^) by comparing against the UNITE fungal ITS reference database (version 11.2^53^). This process resulted in the creation of a table of OTUs. OTU sequences not belonging to Fungi or Oomycota were removed from further analysis. Rarefaction curves were determined for the obtained OTU library. As a result of normalization, two negative samples were removed.

The function of fungi in the community was determined based on literature data and the DEEMY information system for characterization and determination of ectomycorrhizae (http://www.deemy.de^34,54^) and FungalTraits ver. 1.2.Latin nomenclature was adopted from the Index Fungorum (http://www.indexfungorum.org).

Comparisons between treatments (the comparison of proportions among different fungal phyla) were made using Pearson’s (χ^2^) test of homogeneity and Pearson’s linear correlation coefficients. Values of indices obtained in the quantitative analysis were compared using a two-way analysis of variance.

The statistical analysis of biodiversity was conducted using five indices: the Margalef index; the Shannon diversity index, which was used to determine the species richness of the assemblage; the Shannon evenness index; the Berger-Parker index; and the Simpson index^55^.

### Generalised analysis of similarities and indicator species

To determine if fungal communities differed statistically significantly between soil and root samples (treated as groups), and between the different types of seedling production (SP30, SP21, KOST, SO, SW, and NAT; treated as sites), we applied a non-metric multidimensional scaling (NMDS) technique with a Bray–Curtis dissimilarity matrix and 9999 iterations to visualise patterns of species composition among groups and sites (e.g.^56,57^). NMDS is a technique not for statistical assessment, but simply for visualisation. We followed NMDS with a generalised analysis of similarities (ANOSIM) in order to find separation or correlation between species communities^58,59^. With this, we tested a null hypothesis stating that there were no statistically significant differences between fungal communities based on place of sampling (root or soil) or based on type of seedling production. ANOSIM compares the mean of ranked dissimilarities among groups (soil, root) to the mean of ranked dissimilarities within groups. The related test statistic R is expected to fall between − 1 and + 1, but generally lies between 0 and + 1^58^. The greater the value of R, the more dissimilar are the analysed groups, in terms of fungal community composition. Statistical significance was calculated by permuting the grouping vector to obtain the empirical distribution of the R value under the null hypothesis. We conducted ANOSIM using the *Vegan* package in R environment (R Development Team, 2021), and used 9999 permutations to assess the statistical significance of the test.

### Indicator species analysis

To identify the fungal species that were found more often in one group (soil and root) in comparison to the other, we conduced indicator species analysis^60^. The standard assumption of the analysis was that some fungal species could be related to one group of sites, while the others could be related to more than one group. As mentioned previously, ‘group’ referred to the place of sampling (root or soil) and ‘site’ referred to the type of seedling production (SP3, SP21, KOST, SO, SW, and NAT). The aim of this analysis was to determine which fungal species could be used as indicators of a certain sampling place (soil, root). An indicator value index (iv) was used to measure the association between species and site groups^60^. This index consisted of two components: A and B. Component A was a sample estimate of the probability that the surveyed site belonged to the site group, given the fact that the species had been found at that site. Component B was a sample estimate of the probability of finding the species at sites belonging to the site group^60^. Indicator species analysis was conducted using the *indicspecies* package for R^60^.

### Supplementary Information

Supplementary Information 1.

Supplementary Information 2.

Supplementary Information 3.

## Data Availability

The data presented in this study are available on Baranowska, Marlena (2023). fungal community of 3-year-old silver fir (soil and fine roots).xlsx. figshare. Dataset. https://doi.org/10.6084/m9.figshare.23404070.v1.
