# Supplementary material for: Effects of nursery production methods on fungal community diversity within soil and roots of Abies alba Mill
Source: Sci Rep. 2023 Dec 2;13:21284. doi: 10.1038/s41598-023-48047-y (PMC10693611; doi:10.1038/s41598-023-48047-y)
Supplement: Supplementary file 1 — Supplementary Information 1. [file 41598_2023_48047_MOESM1_ESM.docx]

Nursery I, where 3/0 seedlings and 2/1 transplants were produced (WGS 84 N:50.2304, E:16.7280), exhibited acidic brown soils and anthropised soil, with mid-sized mountains and a forest habitat type of mixed mountain fresh forest. This location also hosted a nursery that produced fir from peat substrate in nursery containers, following the system proposed by J. Kosterkiewicz (Fig. 1). For the production of fir seedlings in this container nursery, the following were used: de-acidified peat with dolomite produced in the Rudy Raciborskie Forest District nursery in Nędza (Poland), with a 50% addition of compost from the forest nursery of the Międzylesie Forest District.

Nursery II included a location under Scots pine shelterwood and natural regeneration in a fir stand (WGS 84: N: 50.2883, E: 16.7001). The location hosted a mixed stand with Scots pine (20% 165-year-old trees), European beech (20% 165-year-old trees), silver fir (20% 165-year-old trees), and Norway spruce (20% 165-year-old and 20% 117-year-old trees). The area exhibited acidic brown soils, with low mountains and a forest habitat type of mixed mountain fresh forest (Fig. 1).

Nursery III was located under Norway spruce shelterwood (WGS 84: N: 50.2000, E: 16.7540), in a stand of Norway spruce (60%) mixed with larch (20%), birch (10%), and European beech (10%; 63 years old). The site exhibited acidic brown soils, with mid-sized mountains and a forest habitat type of mixed mountain fresh forest (Fig 1).
