# Supplementary material for: Effects of nursery production methods on fungal community diversity within soil and roots of Abies alba Mill
Source: Sci Rep. 2023 Dec 2;13:21284. doi: 10.1038/s41598-023-48047-y (PMC10693611; doi:10.1038/s41598-023-48047-y)
Supplement: Supplementary file 2 — Supplementary Information 2. [file 41598_2023_48047_MOESM2_ESM.docx]

| **TAXA** | **IS** | **IIS** | **IIIS** | **IVS** | **VS** | **VIS** | **IR** | **IIR** | **IIIR** | **IVR** | **VR** | **VIR** | **ORDER** | **SIMILARITY [%]** | **TROPHIC GROUP** |
| --- | --- | --- | --- | --- | --- | --- | --- | --- | --- | --- | --- | --- | --- | --- | --- |
| **Ascomycota** | | | | | | | | | | | | | | | |
| *Absconditella* sp. | 0.000 | 0.000 | 0.000 | 0.039 | 0.000 | 0.000 | 0.004 | 0.046 | 0.000 | 1,200 | 0.000 | 0.000 | Ostropales | 99 | L |
| *Acarospora macrospora* subsp. *macrospora* | 0.000 | 0.000 | 0.000 | 0.000 | 0.000 | 0.000 | 0.000 | 0.005 | 0.000 | 0.000 | 0.000 | 0.000 | Acarosporales | 100 | L |
| Acarosporaceae | 0.000 | 0.000 | 0.000 | 0.000 | 0.000 | 0.000 | 0.000 | 0.000 | 0.000 | 0.000 | 0.000 | 0.000 |  | 98 | L |
| Acarosporales | 0.001 | 0.000 | 0.000 | 0.000 | 0.021 | 0.000 | 0.000 | 0.000 | 0.000 | 0.000 | 0.000 | 0.000 |  | 98 | L |
| *Acephala* sp. + ***A. applanata*** + *A. macrosclerotiorum* | 0.023 | 0.013 | 0.000 | 0.271 | 0.214 | 0.015 | 0.004 | 0.251 | 0.464 | 4.200 | 0.658 | 9.893 | Helotiales | 97-100 | M |
| *Acidomelania panicicola* | 0.000 | 0.000 | 0.000 | 0.000 | 0.000 | 0.000 | 0.000 | 0.015 | 0.000 | 0.000 | 0.000 | 0.000 | Helotiales | 99 | S |
| ***Acremonium* sp.** + *A. alternatum* + *A. utilum* | 0.181 | 0.069 | 0.000 | 0.000 | 0.000 | 0.008 | 0.000 | 0.025 | 0.000 | 0.009 | 0.000 | 0.017 | Hypocreales | 98-100 | S |
| *Acrogenospora carmichaeliana* | 0.000 | 0.000 | 0.000 | 0.000 | 0.000 | 0.000 | 0.000 | 0.001 | 0.000 | 0.000 | 0.000 | 0.000 | Minutisphaerales | 98 | S |
| *Adisciso tricellulare* | 0.000 | 0.000 | 0.000 | 0.000 | 0.000 | 0.000 | 0.225 | 0.000 | 0.000 | 0.009 | 0.000 | 0.003 | Amphisphaeriales | 100 | S |
| *Alatospora acuminata* + *A. flagellata* | 0.023 | 0.000 | 0.000 | 0.000 | 0.003 | 0.001 | 0.000 | 0.008 | 0.000 | 0.000 | 0.021 | 0.003 | Helotiales | 99-100 | S |
| *Angustimassarina acerina* | 0.000 | 0.000 | 0.000 | 0.000 | 0.000 | 0.000 | 0.004 | 0.000 | 0.000 | 0.000 | 0.000 | 0.001 | Pleosporales | 99 | S |
| Annulatascaceae | 0.000 | 0.000 | 0.000 | 0.000 | 0.001 | 0.000 | 0.000 | 0.000 | 0.000 | 0.000 | 0.000 | 0.000 | Annulatascales | 100 | S |
| *Apiognomonia errabunda* | 0.000 | 0.000 | 0.000 | 0.000 | 0.001 | 0.000 | 0.458 | 0.001 | 0.170 | 0.003 | 0.009 | 0.028 | Diaporthales | 99 | P |
| *Apodus deciduus* | 0.017 | 0.013 | 0.000 | 0.038 | 0.004 | 0.000 | 0.000 | 0.008 | 0.000 | 0.009 | 0.002 | 0.000 | Sordariales | 98 | S |
| ***Arachnopeziza*** sp. | 0.000 | 0.000 | 0.000 | 0.000 | 0.000 | 0.000 | 0.151 | 0.000 | 0.000 | 0.000 | 0.000 | 0.000 | Helotiales | 99 | S |
| ***Archaeorhizomyces* sp.** + *A. borealis* | 0.592 | 0.225 | 0.000 | 0.252 | 0.207 | 0.523 | 0.000 | 4.822 | 0.356 | 0.040 | 0.002 | 0.103 | Helotiales | 99-100 | S |
| Archaeorhizomycetales | 0.000 | 0.000 | 0.000 | 0.008 | 0.000 | 0.000 | 0.000 | 0.000 | 0.000 | 0.000 | 0.000 | 0.000 |  | 100 | S |
| Archaeorhizomycetes | 0.029 | 0.013 | 0.000 | 0.000 | 0.000 | 0.026 | 0.000 | 0.163 | 0.000 | 0.000 | 0.000 | 0.032 |  | 100 | S |
| ***Arthrobotrys conoides*** + *A. oligosporus* | 0.169 | 0.009 | 0.000 | 0.000 | 0.000 | 0.001 | 0.000 | 0.011 | 0.000 | 0.000 | 0.000 | 0.000 | Orbiliales | 98-99 | A |
| *Arthrographis* sp. + *A. pinicola* | 0.018 | 0.000 | 0.000 | 0.000 | 0.000 | 0.000 | 0.000 | 0.004 | 0.000 | 0.000 | 0.000 | 0.000 | Incertae Sedis | 98-99 | A |
| *Articulospora* sp. | 0.000 | 0.000 | 0.000 | 0.007 | 0.000 | 0.000 | 0.000 | 0.000 | 0.000 | 0.000 | 0.000 | 0.018 | Helotiales | 99 | S |
| *Ascobolus* sp. | 0.809 | 0.378 | 0.000 | 0.01 | 0.000 | 0.012 | 0.000 | 0.057 | 0.000 | 0.000 | 0.000 | 0.000 | Pezizales | 98 | S |
| Ascomycota | 1.641 | 0.722 | 0.075 | 3.978 | 1.137 | 0.613 | 2.023 | 1.276 | 2.252 | 0.782 | 0.357 | 3.893 |  | 100 |  |
| *Aspergillus* sp. + *A. costiformis* + *A. inflatus*  + *A. kassunensis* + *A. niger* + *A. parvulus*  + *A. penicillioides* + *A. pseudodeflectus*  + ***A. tardus*** | 0.061 | 0.056 | 0.000 | 0.105 | 0.412 | 0.087 | 0.000 | 0.461 | 0.813 | 0.087 | 0.000 | 0.163 | Eurotiales | 97-100 | A |
| *Atractospora decumbens* | 0.000 | 0.000 | 0.000 | 0.000 | 0.000 | 0.000 | 0.000 | 0.000 | 0.000 | 0.194 | 0.000 | 0.000 | Incertae Sedis | 99 | S |
| *Aureobasidium namibiae* | 0.000 | 0.000 | 0.000 | 0.001 | 0.000 | 0.000 | 0.000 | 0.000 | 0.000 | 0.000 | 0.000 | 0.000 | Dothideales | 99 | P |
| *Austroplaca cirrochrooides* | 0.000 | 0.000 | 0.000 | 0.000 | 0.002 | 0.000 | 0.000 | 0.000 | 0.000 | 0.000 | 0.000 | 0.000 | Teloschistales | 98 | L |
| *Auxarthron californiense* + *A. umbrinum* | 0.035 | 0.000 | 0.000 | 0.003 | 0.004 | 0.001 | 0.000 | 0.006 | 0.000 | 0.000 | 0.000 | 0.000 | Onygenales | 97-99 | E |
| *Beauveria* sp. + *B. bassiana* + *B. caledonica*  + ***B. pseudobassiana*** | 0.000 | 0.004 | 0.000 | 0.02 | 0.000 | 0.000 | 0.282 | 0.002 | 0.108 | 0.002 | 0.002 | 0.018 | Hypocreales | 98-100 | E |
| Bionectriaceae | 0.000 | 0.071 | 0.000 | 0.001 | 0.002 | 0.000 | 0.000 | 0.001 | 0.000 | 0.000 | 0.000 | 0.000 | Hypocreales | 99 | S |
| *Bulgaria inquinans* | 0.000 | 0.000 | 0.000 | 0.000 | 0.000 | 0.002 | 0.000 | 0.000 | 0.000 | 0.000 | 0.000 | 0.000 | Helotiales | 99 | S |
| *Cadophora* sp. + ***C. finlandica*** + *C.* *orchidicola* | 0.070 | 0.054 | 0.000 | 0.153 | 0.178 | 0.043 | 0.695 | 0.557 | 0.037 | 2.080 | 1.414 | 1.119 | Helotiales | 98-100 | P |
| ***Calcarisporiella* sp.** + *C. thermophila* | 0.018 | 0.002 | 0.000 | 0.000 | 0.000 | 0.000 | 0.000 | 0.004 | 0.000 | 0.000 | 0.000 | 0.000 | Calcarisporiellales | 98-99 | S |
| *Calcarisporium arbusculum* | 0.000 | 0.000 | 0.000 | 0.007 | 0.009 | 0.002 | 0.000 | 0.000 | 0.000 | 0.000 | 0.000 | 0.000 | Hypocreales | 99 | S |
| *Calicium salicinum* | 0.000 | 0.000 | 0.000 | 0.000 | 0.003 | 0.000 | 0.000 | 0.000 | 0.000 | 0.003 | 0.000 | 0.011 | Caliciales | 97 | L |
| *Caloplaca obscurella* | 0.000 | 0.000 | 0.000 | 0.069 | 0.012 | 0.000 | 0.000 | 0.000 | 0.000 | 0.000 | 0.002 | 0.000 | Teloschistales | 98 | L |
| *Calyptrozyma* sp. | 0.000 | 0.000 | 0.000 | 0.000 | 0.000 | 0.000 | 0.000 | 0.009 | 0.000 | 0.000 | 0.000 | 0.000 | Incertae Sedis | 98 | S |
| *Canalisporium caribense* | 0.000 | 0.000 | 0.000 | 0.000 | 0.000 | 0.000 | 0.000 | 0.001 | 0.000 | 0.000 | 0.000 | 0.000 | Incertae Sedis | 97 | S |
| ***Candida* sp.** + *C. fructus* + *C. subhashii* | 0.000 | 0.002 | 0.126 | 0.000 | 0.010 | 0.008 | 0.004 | 0.076 | 0.022 | 0.000 | 0.032 | 0.007 | Saccharomycetales | 99-100 | A |
| ***Capnobotryella* sp.** + *C. renispora* | 0.015 | 0.002 | 0.000 | 0.000 | 0.003 | 0.022 | 0.000 | 0.109 | 0.000 | 0.000 | 0.000 | 0.000 | Capnodiales | 98-99 | S |
| Capnodiales | 0.077 | 0.028 | 0.000 | 0.026 | 0.049 | 0.052 | 0.000 | 0.035 | 0.356 | 0.035 | 0.012 | 0.021 |  | 100 | P |
| *Capronia* sp. | 0.000 | 0.000 | 0.000 | 0.001 | 0.002 | 0.000 | 0.000 | 0.000 | 0.000 | 0.000 | 0.000 | 0.001 | Chaetothyriales | 99 | S |
| *Cenococcum* sp. + *C. geophilum* | 0.034 | 0.004 | 0.000 | 0.264 | 0.003 | 1.392 | 0.004 | 0.200 | 0.003 | 0.000 | 0.490 | 1.017 | Incertae Sedis | 98-99 | M |
| *Cercophora* sp. | 0.016 | 0.037 | 0.000 | 0.000 | 0.000 | 0.000 | 1,000 | 0.005 | 0.000 | 0.000 | 0.000 | 0.001 | Sordariales | 98 | S |
| Chaetomiaceae | 0.261 | 0.156 | 0.000 | 0.153 | 0.036 | 0.003 | 0.000 | 0.032 | 0.000 | 0.000 | 0.000 | 0.000 | Sordariales | 100 | S |
| ***Chaetomium* sp.** *+ Ch. grande* + *Ch. succineum* | 0.247 | 0.037 | 0.000 | 0.005 | 0.002 | 0.005 | 0.000 | 0.009 | 0.000 | 0.000 | 0.002 | 0.000 | Sordariales | 98-99 | S |
| *Chaetosphaeria* sp. + **Ch. innumera** + *Ch. vermicularioides* | 0.062 | 0.011 | 0.000 | 0.000 | 0.055 | 0.001 | 0.000 | 0.001 | 0.084 | 0.088 | 0.000 | 0.004 | Chaetosphaeriales | 97-100 | S |
| Chaetosphaeriaceae | 0.002 | 0.000 | 0.000 | 0.033 | 0.018 | 0.023 | 0.000 | 0.001 | 0.000 | 0.019 | 0.000 | 0.001 | Chaetosphaeriales | 100 | S |
| Chaetothyriales | 0.019 | 0.026 | 0.000 | 0.177 | 0.044 | 0.065 | 0.000 | 0.191 | 0.749 | 0.014 | 0.968 | 0.024 |  | 100 |  |
| ***Chalara sp.*** + ***Ch. hyalocuspica*** + *Ch. longipes*  + *Ch. microspora* + *Ch. pseudoaffinis*  + *Ch. piceaeabietis* | 0.293 | 0.082 | 0.000 | 3.934 | 0.233 | 0.046 | 2.526 | 0.245 | 0.046 | 0.307 | 0.644 | 0.285 | Incertae Sedis | 98-100 | P |
| *Cheirosporium triseriale* | 0.005 | 0.000 | 0.000 | 0.000 | 0.000 | 0.000 | 1.848 | 0.000 | 0.000 | 0.000 | 0.201 | 0.027 | Pleosporales | 98 | S |
| *Chlamydotubeufia khunkornensis* | 0.008 | 0.011 | 0.000 | 0.000 | 0.000 | 0.000 | 0.000 | 0.000 | 0.000 | 0.000 | 0.000 | 0.000 | Pleosporales | 99 |  |
| *Chloridium* sp. | 0.000 | 0.000 | 0.000 | 0.069 | 0.000 | 0.001 | 0.000 | 0.003 | 0.000 | 0.000 | 0.000 | 0.000 | Chaetosphaeriales | 100 | S |
| *Chrysothrix* sp. | 0.000 | 0.000 | 0.000 | 0.000 | 0.000 | 0.000 | 0.000 | 0.000 | 0.213 | 0.000 | 0.000 | 0.000 | Arthoniales | 100 | L |
| *Ciliciopodium brevipes* | 0.000 | 0.002 | 0.000 | 0.000 | 0.000 | 0.004 | 0.000 | 0.000 | 0.000 | 0.000 | 0.000 | 0.000 | Hypocreales | 99 | P |
| *Ciliophora* sp. | 0.089 | 0.558 | 0.000 | 0.367 | 0.104 | 0.072 | 0.000 | 0.682 | 0.003 | 0.136 | 0.047 | 0.316 | Incertae Sedis | 98 | S |
| *Cirrenalia macrocephala* | 0.006 | 0.043 | 0.000 | 0.000 | 0.000 | 0.001 | 0.000 | 0.001 | 0.000 | 0.000 | 0.000 | 0.000 | Microascales | 99 | S |
| *Cistella* sp. + *C. acuum* + *C. albidolutea* | 0.011 | 0.011 | 0.000 | 0.012 | 0.004 | 0.001 | 0.000 | 0.000 | 0.000 | 0.000 | 0.000 | 0.000 | Helotiales | 98-99 | S |
| *Citeromyces siamensis* | 0.000 | 0.000 | 0.000 | 0.208 | 0.023 | 0.000 | 0.000 | 0.000 | 0.000 | 0.000 | 0.000 | 0.000 | Saccharomycetales | 97 |  |
| ***Cladophialophora sp.*** + *C. chaetospira*  + *C. minutissima* | 0.014 | 0.004 | 0.000 | 0.324 | 0.053 | 0.062 | 0.098 | 0.19 | 0.232 | 0.178 | 0.191 | 0.302 | Chaetothyriales | 97-99 | P |
| ***Cladorrhinum brunnescens*** + *C. bulbillosum*  + *C. flexuosum* | 0.121 | 0.024 | 0.000 | 0.000 | 0.000 | 0.003 | 0.000 | 0.011 | 0.000 | 0.000 | 0.000 | 0.000 | Sordariales | 98-99 | A |
| *Clonostachys* sp. + ***C. divergens*** + *C. rosea* | 0.006 | 0.006 | 0.000 | 0.017 | 0.003 | 0.001 | 0.061 | 0.018 | 0.093 | 0.049 | 0.026 | 0.028 | Hypocreales | 98-100 | A |
| *Coccomyces australis* | 0.000 | 0.000 | 0.000 | 0.000 | 0.000 | 0.000 | 0.000 | 0.000 | 0.000 | 0.466 | 0.000 | 0.000 | Rhytismatales | 99 | P |
| *Colletotrichum antirrhinicola*  + ***C. cliviae*** | 0.002 | 0.000 | 0.000 | 0.144 | 0.002 | 0.007 | 0.000 | 0.019 | 0.000 | 0.000 | 0.000 | 0.000 | Glomerellales | 98-99 | P |
| *Colpoma quercinum* | 0.000 | 0.000 | 0.000 | 0.000 | 0.000 | 0.000 | 0.000 | 0.001 | 0.000 | 0.000 | 0.000 | 0.000 | Rhytismatales | 100 | P |
| *Coniochaeta* sp. *+ C. fodinicola*  + ***C. gigantospora*** + *C. lignicola* | 0.000 | 0.000 | 0.000 | 0.000 | 0.000 | 0.007 | 0.172 | 0.008 | 0.238 | 0.000 | 0.007 | 0.001 | Coniochaetales | 98-100 | P |
| Coniochaetaceae | 0.003 | 0.000 | 0.000 | 0.000 | 0.002 | 0.004 | 0.000 | 0.008 | 0.285 | 0.000 | 0.023 | 0.000 | Coniochaetales | 98-100 | P |
| Coniochaetales | 0.000 | 0.000 | 0.000 | 0.000 | 0.003 | 0.000 | 0.000 | 0.009 | 0.000 | 0.000 | 0.000 | 0.003 |  | 100 | P |
| *Conlarium dupliciascosporum* | 0.014 | 0.004 | 0.000 | 0.000 | 0.000 | 0.000 | 0.000 | 0.001 | 0.000 | 0.000 | 0.000 | 0.000 | Atractosporales | 100 | P |
| *Corallomycetella repens* | 0.000 | 0.000 | 0.000 | 0.000 | 0.000 | 0.000 | 0.008 | 0.000 | 0.000 | 0.04 | 0.000 | 0.014 | Incertae Sedis | 100 | S |
| *Cordyceps brongniartii* + *C. cateniannulata* | 0.001 | 0.000 | 0.000 | 0.000 | 0.000 | 0.000 | 0.282 | 0.000 | 0.282 | 0.000 | 0.000 | 0.000 | Hypocreales | 99-100 | E |
| *Corynespora smithii* | 0.000 | 0.000 | 0.000 | 0.000 | 0.000 | 0.001 | 0.000 | 0.000 | 0.000 | 0.000 | 0.000 | 0.000 | Pleosporales | 99 | S |
| Crocicreas | 0.000 | 0.000 | 0.000 | 0.056 | 0.009 | 0.006 | 0.000 | 0.000 | 0.000 | 0.000 | 0.000 | 0.000 | Helotiales | 100 | S |
| *Cryptosporiopsis* sp. | 0.000 | 0.000 | 0.000 | 0.056 | 0.027 | 0.000 | 0.000 | 0.000 | 0.000 | 0.000 | 0.000 | 0.000 | Helotiales | 99 | P |
| *Custingophora olivacea* | 0.006 | 0.037 | 0.000 | 0.000 | 0.000 | 0.000 | 0.000 | 0.001 | 0.000 | 0.000 | 0.000 | 0.000 | Incertae Sedis | 99 | S |
| *Cylindrosympodium* sp. + *C. lauri* | 0.006 | 0.004 | 0.000 | 0.000 | 0.000 | 0.015 | 0.000 | 0.029 | 0.000 | 0.000 | 0.000 | 0.000 | Pleosporales | 99-100 | S |
| *Dactylaria lanosa* | 0.000 | 0.000 | 0.000 | 0.004 | 0.013 | 0.007 | 0.000 | 0.009 | 0.000 | 0.000 | 0.000 | 0.000 | Helotiales | 99 | P |
| *Dactylella mammillata* + *D. rhopalota*  + *D. ellipsospora* | 0.002 | 0.000 | 0.000 | 0.058 | 0.000 | 0.000 | 0.000 | 0.007 | 0.000 | 0.000 | 0.028 | 0.000 | Incertae Sedis | 98-99 | A |
| *Dendryphion nanum* | 0.225 | 0.112 | 0.000 | 0.000 | 0.000 | 0.002 | 0.082 | 0.018 | 0.000 | 0.000 | 0.000 | 0.000 | Pleosporales | 99 | S |
| Dermateaceae | 1.019 | 0.037 | 0.000 | 0.269 | 0.187 | 0.049 | 1.206 | 0.174 | 0.575 | 0.549 | 1.031 | 0.364 | Dermateaceae | 100 | S |
| *Desertella* sp. | 0.038 | 0.000 | 0.000 | 0.000 | 0.000 | 0.000 | 0.000 | 0.003 | 0.000 | 0.000 | 0.000 | 0.000 | Incertae Sedis | 99 |  |
| *Desmazierella acicola* | 0.000 | 0.000 | 0.000 | 0.022 | 0.000 | 0.000 | 0.000 | 0.000 | 0.000 | 0.021 | 0.000 | 0.000 | Pezizales | 99 | S |
| *Devriesia* sp. | 0.053 | 0.043 | 0.000 | 0.000 | 0.000 | 0.001 | 0.000 | 0.004 | 0.000 | 0.000 | 0.000 | 0.000 | Incertae Sedis | 100 | S |
| Diaporthales | 0.030 | 0.019 | 0.000 | 0.000 | 0.000 | 0.001 | 0.192 | 0.016 | 0.000 | 0.000 | 0.000 | 0.000 |  | 100 | P |
| *Diaporthe* sp. + *D. cynaroidis* + *D. helicis* | 0.000 | 0.000 | 0.000 | 0.000 | 0.001 | 0.000 | 0.188 | 0.004 | 0.000 | 0.016 | 0.000 | 0.000 | Diaporthales | 98-100 | P |
| *Diatrype stigma* | 0.000 | 0.000 | 0.000 | 0.000 | 0.000 | 0.000 | 0.000 | 0.000 | 0.059 | 0.000 | 0.000 | 0.000 | Xylariales | 99 | S |
| *Dictyocatenulata* sp. | 0.000 | 0.004 | 0.000 | 0.000 | 0.000 | 0.003 | 0.000 | 0.008 | 0.000 | 0.000 | 0.000 | 0.000 | Incertae Sedis | 99 | S |
| *Didymella dactylidis + D. protuberans* | 0.001 | 0.000 | 0.000 | 1.147 | 0.009 | 0.000 | 0.208 | 0.004 | 0.037 | 0.619 | 0.000 | 0.314 | Pleosporales | 99-100 |  |
| *Didymellaceae* | 0.000 | 0.000 | 0.000 | 0.000 | 0.000 | 0.000 | 0.000 | 0.000 | 0.229 | 0.000 | 0.000 | 0.000 | Pleosporales | 100 | S |
| *Diplodia intermedia* | 0.000 | 0.000 | 0.000 | 0.000 | 0.000 | 0.000 | 0.012 | 0.003 | 0.009 | 0.000 | 0.000 | 0.006 | Botryosphaeriales | 98 | P |
| *Discosia pseudoartocreas* | 0.005 | 0.000 | 0.000 | 0.000 | 0.001 | 0.000 | 0.000 | 0.000 | 0.000 | 0.000 | 0.000 | 0.000 | Amphisphaeriales | 99 | P |
| Dothideomycetes | 0.112 | 0.017 | 0.000 | 1.112 | 0.169 | 0.071 | 0.282 | 0.125 | 0.235 | 0.899 | 0.04 | 0.149 |  | 100 | P |
| *Drechmeria balanoides* + *D. campanulata*  + *D. zeospora* | 0.001 | 0.000 | 0.000 | 0.000 | 0.001 | 0.006 | 0.000 | 0.007 | 0.000 | 0.02 | 0.021 | 0.000 | Hypocreales | 98-100 | E |
| *Drechslera* sp. | 0.017 | 0.000 | 0.000 | 0.000 | 0.017 | 0.000 | 0.000 | 0.003 | 0.000 | 0.01 | 0.007 | 0.000 | Pleosporales | 99 | P |
| *Drechslerella* sp. | 0.000 | 0.000 | 0.000 | 0.000 | 0.000 | 0.001 | 0.000 | 0.004 | 0.000 | 0.000 | 0.000 | 0.000 | Orbiliales | 97 | P |
| *Elaphomyces granulatus* + ***E. muricatus*** | 0.006 | 0.002 | 0.000 | 0.000 | 0.010 | 0.799 | 0.000 | 0.05 | 1.553 | 0.000 | 0.000 | 0.000 | Elaphomycetales | 99-100 | M |
| *Epicoccum nigrum* | 0.001 | 0.000 | 0.000 | 0.001 | 0.000 | 0.000 | 0.008 | 0.003 | 0.015 | 0.000 | 0.000 | 0.024 | Pleosporales | 99 | S |
| ***Erysiphe euonymicola*** + *E. hypophylla* | 0.000 | 0.000 | 0.000 | 0.000 | 0.000 | 0.000 | 1.218 | 0.003 | 0.000 | 0.000 | 0.000 | 0.029 | Erysiphales | 99-100 | P |
| Eurotiales | 0.007 | 0.000 | 0.000 | 0.000 | 0.000 | 0.013 | 0.000 | 0.048 | 0.096 | 0.000 | 0.000 | 0.000 |  | 100 |  |
| *Exophiala equina* + *E. moniliae*  + *E. opportunistica* + *E. sideris* + *E. xenobiotica* | 0.001 | 0.004 | 0.000 | 0.000 | 0.005 | 0.002 | 0.233 | 0.020 | 0.000 | 0.000 | 0.049 | 0.008 | Chaetothyriales | 97-100 | S |
| *Fimetariella* sp. | 0.000 | 0.000 | 0.000 | 0.000 | 0.027 | 0.012 | 0.000 | 0.251 | 0.08 | 0.035 | 0.149 | 0.010 | Sordariales | 97 | S |
| *Foliocryphia eucalypti* | 0.000 | 0.000 | 0.000 | 0.038 | 0.000 | 0.000 | 0.000 | 0.000 | 0.000 | 0.000 | 0.000 | 0.000 | Diaporthales | 99 |  |
| ***Fusarium* sp.** + *F. asiaticum* + *F. concolor*  + ***F. oxysporum*** | 1.312 | 0.262 | 0.000 | 0.633 | 0.014 | 0.015 | 0.11 | 0.235 | 0.089 | 0.000 | 0.035 | 0.014 | Hypocreales | 98-100 | P |
| *Fusicolla violacea* | 0.001 | 0.000 | 0.000 | 0.000 | 0.000 | 0.000 | 0.000 | 0.001 | 0.000 | 0.000 | 0.000 | 0.000 | Hypocreales | 99 | P |
| Geoglossaceae | 0.000 | 0.000 | 0.000 | 0.000 | 0.000 | 0.000 | 0.000 | 0.000 | 0.133 | 0.000 | 0.000 | 0.000 | Geoglossales | 100 |  |
| *Geoglossum geesterani* | 0.000 | 0.000 | 0.000 | 0.003 | 0.000 | 0.000 | 0.000 | 0.000 | 0.000 | 0.000 | 0.000 | 0.000 | Geoglossales | 99 | S |
| *Geomyces* sp. + ***G. asperulatus*** + *G. auratus* | 0.041 | 0.033 | 0.000 | 0.250 | 0.328 | 0.068 | 0.478 | 0.26 | 0.000 | 1.110 | 0.295 | 0.140 | Incertae Sedis | 99-100 | S |
| *Graphostroma platystomum* | 0.034 | 0.000 | 0.000 | 0.000 | 0.000 | 0.000 | 0.000 | 0.000 | 0.000 | 0.000 | 0.000 | 0.000 | Xylariales | 99 | S |
| *Gymnostellatospora canadensis* | 0.002 | 0.000 | 0.000 | 0.000 | 0.004 | 0.001 | 0.000 | 0.001 | 0.000 | 0.000 | 0.000 | 0.000 | Incertae Sedis | 98 | S |
| ***Gyoerffyella* sp.** + *G. entomobryoides* | 0.088 | 0.000 | 0.000 | 4.942 | 0.367 | 0.000 | 0.196 | 0.013 | 0.056 | 6.144 | 0.315 | 4.588 | Incertae Sedis | 99-100 | S |
| *Halokirschsteiniothelia maritima* | 0.170 | 0.050 | 0.000 | 0.014 | 0.000 | 0.003 | 0.495 | 0.027 | 0.000 | 0.000 | 0.000 | 0.000 | Mytilinidiales | 99 | S |
| Halosphaeriaceae | 0.022 | 0.032 | 0.000 | 0.000 | 0.000 | 0.000 | 0.454 | 0.008 | 0.232 | 0.014 | 0.012 | 0.000 | Microascales | 100 |  |
| *Hanseniaspora uvarum* | 0.000 | 0.000 | 0.000 | 0.000 | 0.000 | 0.000 | 0.000 | 0.000 | 0.006 | 0.000 | 0.000 | 0.004 | Saccharomycetales | 99 | S |
| *Harposporium subuliforme* | 0.000 | 0.000 | 0.000 | 0.003 | 0.001 | 0.000 | 0.000 | 0.000 | 0.000 | 0.000 | 0.000 | 0.000 | Hypocreales | 99 | E |
| Harzia cameroonensis + *H. sphaerospora* | 0.000 | 0.002 | 0.000 | 0.008 | 0.124 | 0.008 | 0.000 | 0.005 | 0.000 | 0.000 | 0.000 | 0.000 | Melanosporales | 99-100 | S |
| *Helgardia* sp. | 0.000 | 0.000 | 0.000 | 0.024 | 0.000 | 0.000 | 0.000 | 0.000 | 0.000 | 0.003 | 0.000 | 0.001 | Incertae Sedis | 99 | P |
| *Helicoma dennisii* | 0.006 | 0.002 | 0.000 | 0.000 | 0.000 | 0.002 | 0.000 | 0.038 | 0.000 | 0.000 | 0.000 | 0.000 | Tubeufiales | 99 | S |
| Helotiaceae | 0.034 | 0.004 | 0.000 | 0.211 | 0.099 | 0.565 | 1.627 | 0.051 | 4.176 | 0.423 | 0.009 | 0.097 | Helotiales | 99 |  |
| Helotiales | 0.740 | 0.264 | 0.000 | 1.555 | 2.011 | 1.219 | 1.545 | 0.957 | 3.860 | 1.850 | 2.964 | 1.481 |  | 100 |  |
| Herpotrichiellaceae | 0.049 | 0.002 | 0.000 | 0.000 | 0.029 | 0.068 | 0.000 | 0.089 | 0.000 | 0.000 | 0.000 | 0.027 | Chaetothyriales | 100 |  |
| ***Humicola* sp.** + *H. grisea* | 0.022 | 0.013 | 0.000 | 0.004 | 0.001 | 0.001 | 0.029 | 0.008 | 0.006 | 0.014 | 0.002 | 0.000 | Sordariales | 99-100 | S |
| H*yalopeziza* sp. | 0.001 | 0.013 | 0.000 | 0.000 | 0.001 | 0.001 | 0.000 | 0.000 | 0.000 | 0.000 | 0.014 | 0.000 | Helotiales | 99 | L |
| *Hyalorbilia inflatula* | 0.001 | 0.000 | 0.000 | 0.000 | 0.000 | 0.000 | 0.000 | 0.001 | 0.000 | 0.000 | 0.000 | 0.000 | Orbiliales | 99 | S |
| *Hyaloscypha aureliella* + *H. monodictys* | 0.002 | 0.022 | 0.000 | 0.000 | 0.000 | 0.000 | 0.000 | 0.001 | 0.000 | 0.000 | 0.000 | 0.000 | Helotiales | 99-100 | S |
| Hyaloscyphaceae | 0.003 | 0.002 | 0.000 | 0.000 | 0.008 | 0.468 | 0.000 | 0.028 | 0.000 | 0.005 | 0.000 | 0.000 | Helotiales | 100 | S |
| *Hyaloseta nolinae* | 0.000 | 0.000 | 0.000 | 0.000 | 0.000 | 0.003 | 0.000 | 0.000 | 0.000 | 0.000 | 0.000 | 0.000 | Hypocreales | 99 |  |
| ***Hydnotrya cerebriformis*** + *H. michaelis*  + *H. tulasnei* | 0.000 | 0.000 | 0.000 | 0.000 | 0.000 | 0.000 | 0.118 | 0.026 | 0.025 | 0.114 | 9.754 | 0.024 | Pezizales | 98-100 | M |
| *Hymenoscyphus* sp. | 0.000 | 0.002 | 0.000 | 0.000 | 0.001 | 0.105 | 0.000 | 0.016 | 0.65 | 0.003 | 0.000 | 0.000 | Helotiales | 99 | S |
| *Hyphodiscus* sp. | 0.000 | 0.000 | 0.000 | 0.000 | 0.000 | 0.000 | 0.000 | 0.004 | 0.000 | 0.000 | 0.000 | 0.000 | Helotiales | 99 | S |
| Hypocreaceae | 0.003 | 0.000 | 0.000 | 0.000 | 0.000 | 0.000 | 0.000 | 0.023 | 0.000 | 0.000 | 0.000 | 0.000 | Hypocreales | 100 |  |
| Hypocreales | 0.878 | 0.272 | 0.000 | 0.021 | 0.141 | 1.170 | 6.888 | 5.206 | 0.498 | 0.045 | 0.410 | 0.008 |  | 100 |  |
| *Hypomyces* sp. + *H. microspermus*  + *H. ochraceus* + *H. perniciosus* | 0.008 | 0.000 | 0.000 | 0.008 | 0.011 | 0.106 | 0.000 | 0.044 | 0.000 | 0.000 | 0.000 | 0.000 | Hypocreales | 99-100 | E |
| *Hypotrachyna physcioidea* | 0.000 | 0.000 | 0.000 | 0.000 | 0.000 | 0.000 | 0.000 | 0.003 | 0.000 | 0.000 | 0.000 | 0.000 | Lecanorales | 99 | L |
| *Hypoxylon fragiforme* | 0.000 | 0.000 | 0.000 | 0.000 | 0.000 | 0.000 | 0.041 | 0.000 | 0.000 | 0.000 | 0.000 | 0.000 | Xylariales | 99 | S |
| Hysteriales | 0.002 | 0.000 | 0.000 | 0.000 | 0.000 | 0.003 | 0.000 | 0.029 | 0.000 | 0.000 | 0.000 | 0.000 |  | 100 | P |
| *Ilyonectria morspanacis* + ***I. robusta*** | 0.104 | 0.039 | 0.000 | 0.026 | 0.067 | 0.004 | 1.983 | 1.057 | 0.232 | 0.333 | 0.541 | 0.693 | Hypocreales | 99-100 | P |
| *Infundichalara* sp. + ***I. minuta*** | 0.005 | 0.013 | 0.000 | 0.004 | 0.009 | 0.004 | 0.000 | 0.001 | 0.121 | 0.002 | 0.026 | 0.276 | Helotiales | 99-100 | S |
| *Issatchenkia orientalis* | 0.000 | 0.000 | 0.000 | 0.000 | 0.000 | 0.003 | 0.000 | 0.013 | 0.000 | 0.000 | 0.000 | 0.000 | Saccharomycetales | 98 | S |
| *Kabatiella* sp. | 0.000 | 0.000 | 0.000 | 0.000 | 0.000 | 0.000 | 0.000 | 0.008 | 0.000 | 0.000 | 0.000 | 0.000 | Dothideales | 99 | S |
| *Kazachstania naganishii* | 0.000 | 0.000 | 0.000 | 0.000 | 0.000 | 0.000 | 0.000 | 0.008 | 0.000 | 0.000 | 0.000 | 0.000 | Saccharomycetales | 99 | S |
| *Kluyveromyces marxianus* | 0.006 | 0.006 | 0.000 | 0.000 | 0.005 | 0.001 | 0.221 | 0.001 | 0.000 | 0.000 | 0.014 | 0.000 | Saccharomycetales | 99 | S |
| *Knufia peltigerae* | 0.018 | 0.002 | 0.000 | 0.056 | 0.004 | 0.001 | 0.000 | 0.000 | 0.000 | 0.000 | 0.000 | 0.000 | Incertae Sedis | 99 | L |
| Kohlmeyeriopsis medullaris | 0.000 | 0.000 | 0.000 | 0.000 | 0.000 | 0.000 | 0.000 | 0.005 | 0.000 | 0.000 | 0.000 | 0.000 | Incertae Sedis | 100 | S |
| *Kretzschmaria pavimentosa* | 0.000 | 0.000 | 0.000 | 0.000 | 0.001 | 0.000 | 0.000 | 0.000 | 0.000 | 0.000 | 0.000 | 0.000 | Xylariales | 99 | P |
| *Lachancea quebecensis* | 0.000 | 0.000 | 0.000 | 0.03 | 0.000 | 0.000 | 0.000 | 0.000 | 0.000 | 0.000 | 0.000 | 0.000 | Saccharomycetales | 99 |  |
| *Lachnellula calyciformis*  + *L. flavovirens* | 0.000 | 0.000 | 0.000 | 0.000 | 0.000 | 0.000 | 0.008 | 0.000 | 0.068 | 0.000 | 0.000 | 0.08 | Helotiales | 99-100 | P |
| *Lachnum virgineum* | 0.000 | 0.002 | 0.000 | 0.000 | 0.002 | 0.000 | 0.000 | 0.004 | 0.000 | 0.000 | 0.000 | 0.000 | Helotiales | 97 | S |
| *Lanzia echinophila* | 0.026 | 0.000 | 0.000 | 0.004 | 0.000 | 0.001 | 0.000 | 0.000 | 0.000 | 0.000 | 0.000 | 0.000 | Helotiales | 99 | S |
| Lasiosphaeriaceae | 0.512 | 0.000 | 0.000 | 0.000 | 0.004 | 0.005 | 0.147 | 0.033 | 0.000 | 0.01 | 0.000 | 0.007 | Sordariales | 100 | S |
| *Lasiosphaeris hispida* | 0.016 | 0.000 | 0.000 | 0.000 | 0.000 | 0.002 | 0.000 | 0.003 | 0.000 | 0.000 | 0.000 | 0.000 | Sordariales | 99 | S |
| ***Lecanicillium* sp.** + *L. fusisporum*  + *L. kalimantanense* + *L. primulinum* | 0.031 | 0.007 | 0.000 | 0.747 | 0.03 | 0.004 | 0.000 | 0.017 | 0.000 | 0.000 | 0.002 | 0.021 | Incertae Sedis | 97-100 | E |
| Lecanorales | 0.000 | 0.000 | 0.000 | 0.000 | 0.000 | 0.000 | 0.000 | 0.001 | 0.000 | 0.000 | 0.000 | 0.006 |  | 100 | E |
| Lecanoromycetes | 0.009 | 0.002 | 0.000 | 0.047 | 0.043 | 0.054 | 0.000 | 0.009 | 0.068 | 0.000 | 0.000 | 0.01 |  | 100 | E |
| *Lectera longa* | 0.021 | 0.019 | 0.000 | 0.000 | 0.000 | 0.000 | 0.000 | 0.004 | 0.000 | 0.000 | 0.000 | 0.000 | Glomerellales | 99 | P |
| *Lecythophora fasciculata* | 0.073 | 0.134 | 0.000 | 0.000 | 0.000 | 0.003 | 0.000 | 0.011 | 0.000 | 0.000 | 0.000 | 0.000 | Coniochaetales | 99 | P |
| *Leotia lubrica* | 0.001 | 0.002 | 0.000 | 0.000 | 0.000 | 0.248 | 0.000 | 0.019 | 0.724 | 0.000 | 0.000 | 0.000 | Helotiales | 99 | L |
| *Leotiomycetes* sp. | 0.083 | 0.006 | 0.000 | 0.869 | 0.137 | 0.059 | 0.061 | 0.409 | 0.003 | 0.608 | 0.802 | 3.303 | Incertae Sedis | 98 | M |
| *Lepraria caesiella* | 0.000 | 0.000 | 0.000 | 0.000 | 0.000 | 0.005 | 0.000 | 0.001 | 0.000 | 0.000 | 0.000 | 0.000 | Lecanorales | 99 | L |
| *Leptobacillium leptobactrum* | 0.000 | 0.000 | 0.000 | 0.000 | 0.000 | 0.000 | 0.000 | 0.027 | 0.077 | 0.000 | 0.000 | 0.000 | Hypocreales | 99 | P |
| *Leptodiscella* sp. | 0.006 | 0.019 | 0.000 | 0.000 | 0.000 | 0.000 | 0.000 | 0.000 | 0.000 | 0.000 | 0.000 | 0.000 | Muyocopronales | 99 | S |
| ***Leptodontidium* sp.** + *L. trabinellum* | 0.018 | 0.006 | 0.000 | 0.000 | 0.039 | 0.028 | 0.000 | 0.064 | 0.000 | 0.000 | 0.000 | 0.000 | Incertae Sedis | 99-100 | P |
| *Leptogium* sp. | 0.000 | 0.000 | 0.000 | 0.000 | 0.000 | 0.000 | 0.000 | 0.005 | 0.000 | 0.000 | 0.000 | 0.000 | Peltigerales | 99 | L |
| *Leptosphaeria* sp. | 0.005 | 0.060 | 0.000 | 0.000 | 0.201 | 0.001 | 0.000 | 0.004 | 0.241 | 0.198 | 0.000 | 0.065 | Pleosporales | 99 | S |
| *Leuconeurospora* sp. | 0.000 | 0.000 | 0.000 | 0.001 | 0.017 | 0.000 | 0.000 | 0.004 | 0.000 | 0.000 | 0.000 | 0.000 | Incertae Sedis | 98 | S |
| *Lipomyces* sp. + *L. starkeyi* | 0.002 | 0.000 | 0.000 | 0.000 | 0.000 | 0.000 | 0.000 | 0.001 | 0.000 | 0.000 | 0.002 | 0.000 | Saccharomycetales | 99-100 | S |
| *Lopadostoma polynesium* | 0.000 | 0.000 | 0.000 | 0.000 | 0.000 | 0.006 | 0.000 | 0.000 | 0.312 | 0.000 | 0.000 | 0.000 | Xylariales | 99 | S |
| *Lophium* sp. + *L. arboricola* + *L. mytilinum* | 0.015 | 0.002 | 0.000 | 0.000 | 0.002 | 0.021 | 0.000 | 0.061 | 0.000 | 0.000 | 0.000 | 0.001 | Mytilinidiales | 98-100 | S |
| ***Lophodermium conigenum*** + *L. piceae*  + *L. pinastri* + *L. seditiosum* | 0.007 | 0.006 | 0.000 | 0.345 | 0.024 | 0.005 | 1.644 | 0.081 | 1.841 | 0.05 | 0.037 | 0.112 | Rhytismatales | 97-100 | P |
| *Maasoglossum* sp. | 0.000 | 0.000 | 0.000 | 0.043 | 0.000 | 0.019 | 0.004 | 0.707 | 0.000 | 0.000 | 0.000 | 0.000 | Geoglossales | 99 | S |
| Magnaporthaceae | 0.000 | 0.000 | 0.000 | 0.000 | 0.004 | 0.001 | 0.000 | 0.000 | 0.000 | 0.000 | 0.000 | 0.000 | Incertae Sedis | 100 | P |
| *Mariannaea elegans* | 0.022 | 0.000 | 0.000 | 0.016 | 0.086 | 0.000 | 0.000 | 0.029 | 0.000 | 0.126 | 0.007 | 0.000 | Hypocreales | 99 | S |
| *Megacapitula villosa* | 0.002 | 0.000 | 0.000 | 0.000 | 0.000 | 0.001 | 0.000 | 0.000 | 0.000 | 0.000 | 0.000 | 0.000 | Incertae Sedis | 99 | S |
| *Meliniomyces bicolor* + ***M. variabilis*** + *M. vraolstadiae* | 0.000 | 0.002 | 0.000 | 0.041 | 0.000 | 0.023 | 0.004 | 0.305 | 0.507 | 0.024 | 0.884 | 0.108 | Incertae Sedis | 98-100 | S |
| *Metacordyceps chlamydosporia* | 0.000 | 0.000 | 0.000 | 0.000 | 0.000 | 0.001 | 0.000 | 0.005 | 0.000 | 0.000 | 0.000 | 0.000 | Hypocreales | 99 | S |
| *Metapochonia bulbillosa* | 0.016 | 0.019 | 0.000 | 0.118 | 0.183 | 0.068 | 0.000 | 0.06 | 0.000 | 0.043 | 0.056 | 0.037 | Hypocreales | 99 | S |
| *Metarhizium anisopliae* + *M. carneum*  + M. *marquandii* | 0.040 | 0.009 | 0.000 | 0.795 | 0.074 | 0.058 | 0.000 | 0.027 | 0.068 | 0.000 | 0.002 | 0.037 | Hypocreales | 98-100 | E |
| *Micarea assimilata* | 0.000 | 0.000 | 0.000 | 0.000 | 0.000 | 0.000 | 0.000 | 0.003 | 0.000 | 0.000 | 0.000 | 0.000 | Lecanorales | 99 | L |
| Microascaceae | 0.001 | 0.000 | 0.000 | 0.000 | 0.000 | 0.001 | 0.000 | 0.005 | 0.000 | 0.000 | 0.000 | 0.000 | Microascales | 100 |  |
| Microascales | 0.013 | 0.000 | 0.000 | 0.000 | 0.000 | 0.000 | 0.000 | 0.000 | 0.000 | 0.000 | 0.000 | 0.004 |  | 100 |  |
| *Microascus restrictus* | 0.030 | 0.069 | 0.000 | 0.000 | 0.000 | 0.002 | 0.000 | 0.004 | 0.000 | 0.000 | 0.000 | 0.000 | Microascales | 100 | S |
| *Microdochium phragmitis* | 0.000 | 0.000 | 0.000 | 0.000 | 0.012 | 0.000 | 0.000 | 0.000 | 0.000 | 0.000 | 0.000 | 0.000 | Xylariales | 99 | P |
| *Minutisphaera parafimbriatispora* | 0.006 | 0.309 | 0.000 | 0.000 | 0.000 | 0.002 | 0.000 | 0.027 | 0.000 | 0.000 | 0.000 | 0.000 | Minutisphaerales | 99 |  |
| *Mollisia* sp. + *M. cinerea* | 0.007 | 0.000 | 0.000 | 0.008 | 0.000 | 0.000 | 0.000 | 0.006 | 0.000 | 0.000 | 0.000 | 0.008 | Helotiales | 99-100 | S |
| *Monacrosporium drechsleri* | 0.000 | 0.000 | 0.000 | 0.000 | 0.000 | 0.001 | 0.000 | 0.000 | 0.000 | 0.000 | 0.000 | 0.000 | Orbiliales | 99 | A |
| *Monascus eremophilus* | 0.000 | 0.000 | 0.000 | 0.000 | 0.000 | 0.000 | 0.000 | 0.003 | 0.000 | 0.000 | 0.000 | 0.000 | Incertae Sedis | 99 | P |
| *Monochaetia kansensis* | 0.000 | 0.000 | 0.000 | 0.000 | 0.000 | 0.000 | 0.000 | 0.001 | 0.000 | 0.000 | 0.000 | 0.000 | Amphisphaeriales | 99 | S |
| Montagnulaceae | 0.000 | 0.000 | 0.000 | 0.000 | 0.000 | 0.000 | 0.000 | 0.003 | 0.000 | 0.000 | 0.000 | 0.000 | Pleosporales | 100 | P |
| *Mycoarthris corallina* | 0.000 | 0.000 | 0.000 | 0.000 | 0.000 | 0.000 | 0.000 | 0.005 | 0.000 | 0.000 | 0.000 | 0.000 | Incertae Sedis | 99 | S |
| *Mycocentrospora acerina* | 0.000 | 0.000 | 0.000 | 0.026 | 0.000 | 0.000 | 0.000 | 0.000 | 0.000 | 0.000 | 0.023 | 0.028 | Pleosporales | 99 | P |
| *Mycofalcella calcarata* | 0.000 | 0.000 | 0.000 | 0.000 | 0.000 | 0.000 | 0.004 | 0.000 | 0.006 | 0.002 | 0.000 | 0.003 | Incertae Sedis | 99 | S |
| *Mycoleptodiscus* sp. | 0.001 | 0.000 | 0.000 | 0.000 | 0.000 | 0.000 | 0.000 | 0.003 | 0.000 | 0.000 | 0.000 | 0.000 | Muyocopronales | 97 | S |
| *Mycopappus* sp. | 0.017 | 0.000 | 0.000 | 0.000 | 0.002 | 0.025 | 0.000 | 0.004 | 0.000 | 0.000 | 0.000 | 0.000 | Helotiales | 99 | S |
| Mycosphaerellaceae | 0.000 | 0.000 | 0.000 | 0.076 | 0.007 | 0.003 | 0.000 | 0.008 | 0.473 | 0.005 | 0.000 | 0.087 | Capnodiales | 100 |  |
| *Mycothermus thermophilus* | 0.013 | 0.026 | 0.000 | 0.000 | 0.000 | 0.001 | 0.000 | 0.001 | 0.000 | 0.000 | 0.000 | 0.000 | Sordariales | 99 | S |
| Myxotrichaceae | 0.003 | 0.000 | 0.000 | 0.000 | 0.000 | 0.026 | 0.000 | 0.000 | 0.000 | 0.000 | 0.000 | 0.000 | Incertae Sedis | 100 |  |
| *Nadsonia commutata* | 0.000 | 0.000 | 0.000 | 0.01 | 0.003 | 0.040 | 0.000 | 0.000 | 0.000 | 0.000 | 0.000 | 0.000 | Saccharomycetales | 98 | S |
| *Nakazawaea populi* | 0.000 | 0.000 | 0.000 | 0.000 | 0.000 | 0.000 | 0.094 | 0.000 | 0.000 | 0.000 | 0.000 | 0.000 | Saccharomycetales | 99 | S |
| ***Nectria* sp.** + *N. dacryocarpa* | 0.239 | 0.173 | 0.000 | 0.000 | 0.000 | 0.001 | 0.000 | 0.025 | 0.000 | 0.000 | 0.000 | 0.000 | Hypocreales | 99-100 | P |
| Nectriaceae | 0.415 | 0.099 | 0.000 | 0.000 | 0.000 | 0.009 | 0.008 | 0.066 | 0.186 | 0.002 | 0.112 | 0.007 | Hypocreales | 100 | P |
| *Nemania* sp. | 0.000 | 0.000 | 0.000 | 0.000 | 0.000 | 0.000 | 0.000 | 0.006 | 0.000 | 0.000 | 0.000 | 0.000 | Xylariales | 100 | S |
| *Neobulgaria* sp. + *N. pura* | 0.062 | 0.361 | 0.000 | 0.000 | 0.008 | 0.056 | 0.879 | 0.018 | 0.130 | 0.029 | 0.138 | 0.000 | Helotiales | 99-100 | S |
| *Neocosmospora solani* | 0.022 | 0.125 | 0.000 | 0.000 | 0.000 | 0.003 | 0.000 | 0.004 | 0.000 | 0.000 | 0.000 | 0.000 | Hypocreales | 99 | S |
| *Neodevriesia stirlingiae* | 0.000 | 0.000 | 0.000 | 0.000 | 0.000 | 0.000 | 0.000 | 0.000 | 0.000 | 0.000 | 0.000 | 0.000 | Capnodiales | 99 | P |
| *Neonectria* sp. + *N. candida* | 0.027 | 0.03 | 0.000 | 0.000 | 0.002 | 0.000 | 0.155 | 0.102 | 0.009 | 0.000 | 0.021 | 0.001 | Hypocreales | 99-100 | P |
| *Ocellularia punctulata* | 0.002 | 0.007 | 0.000 | 0.525 | 0.516 | 0.069 | 0.000 | 0.019 | 0.093 | 0.012 | 0.000 | 0.035 | Ostropales | 99 | E |
| *Oidiodendron* sp. + *O. chlamydosporicum*  + *O. echinulatum* + ***O. majus*** + *O. pilicola*  + *O. rhodogenum* | 0.023 | 0.006 | 0.000 | 0.712 | 1.305 | 1.511 | 0.000 | 0.451 | 0.876 | 1.203 | 0.056 | 1.101 | Incertae Sedis | 97-100 | S |
| Onygenales | 0.001 | 0.006 | 0.000 | 0.000 | 0.000 | 0.001 | 0.000 | 0.000 | 0.000 | 0.000 | 0.000 | 0.000 |  | 100 |  |
| *Ophidiomyces ophodiicola* | 0.000 | 0.000 | 0.000 | 0.000 | 0.000 | 0.000 | 0.000 | 0.001 | 0.000 | 0.000 | 0.002 | 0.000 | Onygenales | 99 | P |
| Ophiocordycipitaceae | 0.000 | 0.002 | 0.000 | 0.03 | 0.000 | 0.005 | 0.000 | 0.013 | 0.000 | 0.000 | 0.000 | 0.000 | Hypocreales | 100 | E |
| *Ophiosphaerella* sp. | 0.000 | 0.000 | 0.000 | 0.000 | 0.000 | 0.000 | 0.397 | 0.000 | 0.244 | 0.01 | 0.014 | 0.007 | Pleosporales | 99 | P |
| ***Ophiostoma* sp.** + *O. nigrocarpum* + *O. novo-ulmi* | 0.000 | 0.000 | 0.000 | 0.005 | 0.030 | 0.000 | 1.537 | 0.004 | 0.405 | 4.134 | 0.023 | 0.692 | Ophiostomatales | 98-100 | P |
| Ophiostomataceae | 0.000 | 0.000 | 0.000 | 0.000 | 0.000 | 0.000 | 0.004 | 0.000 | 0.003 | 0.000 | 0.005 | 0.007 | Ophiostomatales | 100 | P |
| *Orbilia* sp. | 0.000 | 0.000 | 0.000 | 0.000 | 0.000 | 0.000 | 0.000 | 0.004 | 0.000 | 0.000 | 0.000 | 0.000 | Orbiliales | 99 | S |
| Orbiliaceae | 0.001 | 0.000 | 0.000 | 0.081 | 0.000 | 0.000 | 0.000 | 0.008 | 0.000 | 0.012 | 0.000 | 0.000 |  | 100 | S |
| Orbiliales | 0.002 | 0.000 | 0.000 | 0.000 | 0.002 | 0.018 | 0.000 | 0.024 | 0.000 | 0.000 | 0.000 | 0.015 |  | 100 | S |
| *Orbiliomycetes* sp. | 0.003 | 0.000 | 0.000 | 0.000 | 0.000 | 0.003 | 0.000 | 0.004 | 0.000 | 0.000 | 0.000 | 0.000 | Orbiliales | 97 | S |
| Ostropales | 0.001 | 0.000 | 0.000 | 0.000 | 0.000 | 0.000 | 0.000 | 0.000 | 0.000 | 0.000 | 0.026 | 0.000 |  | 100 | L |
| *Otidea subterranea* | 0.000 | 0.000 | 0.000 | 0.000 | 0.005 | 0.000 | 0.000 | 0.000 | 0.000 | 0.000 | 0.000 | 0.000 | Pezizales | 99 | M |
| *Pachyramichloridium pini* | 0.000 | 0.000 | 0.000 | 0.000 | 0.000 | 0.000 | 0.290 | 0.000 | 0.000 | 0.000 | 0.000 | 0.000 | Capnodiales | 99 | P |
| Pannariaceae | 0.000 | 0.000 | 0.000 | 0.008 | 0.000 | 0.001 | 0.000 | 0.001 | 0.000 | 0.000 | 0.000 | 0.000 | Peltigerales | 100 | L |
| *Paraconiothyrium* sp. | 0.001 | 0.000 | 0.000 | 0.000 | 0.000 | 0.000 | 0.000 | 0.000 | 0.000 | 0.000 | 0.000 | 0.000 | Pleosporales | 99 | P |
| Paraleptosphaeria dryadis | 0.000 | 0.000 | 0.000 | 0.000 | 0.000 | 0.000 | 0.270 | 0.000 | 0.000 | 0.000 | 0.000 | 0.011 | Pleosporales | 99 | S |
| *Paraphaeosphaeria parmeliae* | 0.000 | 0.002 | 0.000 | 0.000 | 0.000 | 0.002 | 0.000 | 0.005 | 0.000 | 0.000 | 0.000 | 0.000 | Pleosporales | 99 | E |
| *Paraphoma fimeti* | 0.000 | 0.067 | 0.000 | 0.000 | 0.000 | 0.000 | 0.000 | 0.000 | 0.000 | 0.000 | 0.000 | 0.000 | Pleosporales | 98 | P |
| *Patinella hyalophaea* | 0.000 | 0.002 | 0.000 | 0.000 | 0.000 | 0.000 | 0.000 | 0.000 | 0.000 | 0.000 | 0.000 | 0.000 | Helotiales | 99 | S |
| *Peltaster fructicola* | 0.000 | 0.000 | 0.000 | 0.000 | 0.000 | 0.000 | 0.000 | 0.001 | 0.000 | 0.000 | 0.000 | 0.000 | Incertae Sedis | 99 | S |
| *Penicillifer pulcher* | 0.000 | 0.000 | 0.000 | 0.000 | 0.014 | 0.000 | 0.000 | 0.000 | 0.000 | 0.012 | 0.000 | 0.000 | Hypocreales | 99 | A |
| *Penicillium* sp. + *P. amaliae* + *P. arianeae*  + *P. atrovenetum* + *P. bialowiezense* + *P. brasilianum* + *P. cairnsense*  + *P. caperatum* + *P. chrysogenum*  + *P. citreonigrum* + *P. decaturense*  + *P. formosanum* + *P. glandicola*  + *P. griseolum* + *P. lanosum* + *P. lapidosum*  + *P. malacaense* + *P. nodositatum*  + *P. onobense* + *P. penicillioides* + *P. raphiae* + *P. riverlandense* + *P. senticosum*  + *P. simplicissimum* + *P. singorense*  + *P. subrubescens* + *P. vasconiae* | 0.779 | 0.030 | 0.010 | 0.705 | 0.254 | 0.103 | 1.467 | 0.638 | 1.893 | 0.249 | 0.300 | 0.678 | Eurotiales | 97-100 | A |
| *Pestalotiopsis unicolor* + *P. verruculosa* | 0.002 | 0.000 | 0.000 | 0.000 | 0.000 | 0.001 | 0.000 | 0.006 | 0.000 | 0.000 | 0.000 | 0.000 | Amphisphaeriales | 99-100 | S |
| *Petriella sordida* | 0.000 | 0.000 | 0.000 | 0.000 | 0.000 | 0.000 | 0.298 | 0.001 | 0.046 | 0.000 | 0.005 | 0.013 | Microascales | 99 | S |
| *Pezicula heterochroma* + *P. sporulosa* | 0.000 | 0.000 | 0.000 | 0.000 | 0.000 | 0.000 | 0.074 | 0.005 | 0.000 | 0.000 | 0.000 | 0.014 | Helotiales | 99-100 | S |
| Pezizaceae | 0.011 | 0.067 | 0.000 | 0.000 | 0.000 | 0.002 | 2.121 | 0.155 | 0.000 | 0.000 | 0.007 | 0.004 | Helotiales | 100 |  |
| Pezizales | 0.005 | 0.002 | 0.000 | 0.164 | 0.050 | 0.010 | 0.110 | 0.025 | 0.000 | 0.009 | 0.000 | 0.000 |  | 100 |  |
| *Pezizomycotina* sp. | 0.007 | 0.004 | 0.000 | 0.000 | 0.000 | 0.004 | 0.000 | 0.041 | 0.000 | 0.000 | 0.005 | 0.000 |  | 99 |  |
| *Pezoloma ciliifera* | 0.000 | 0.000 | 0.000 | 0.000 | 0.000 | 0.010 | 0.000 | 0.000 | 0.000 | 0.000 | 0.000 | 0.000 | Helotiales | 99 | S |
| *Phacidium grevilleae* | 0.021 | 0.007 | 0.000 | 0.005 | 0.363 | 0.168 | 0.000 | 0.030 | 0.217 | 0.028 | 0.000 | 0.320 | Phacidiales | 99 | S |
| *Phaeoacremonium* sp. + *P. occidentale* | 0.001 | 0.000 | 0.000 | 0.018 | 0.000 | 0.001 | 0.000 | 0.019 | 0.207 | 0.000 | 0.000 | 0.000 | Diaporthales | 99-100 | S |
| *Phaeomollisia piceae* | 0.000 | 0.000 | 0.000 | 0.000 | 0.000 | 0.000 | 0.000 | 0.000 | 0.012 | 0.000 | 0.000 | 0.000 | Helotiales | 99 | P |
| Phaeosphaeriaceae | 0.000 | 0.000 | 0.000 | 0.000 | 0.000 | 0.000 | 0.004 | 0.169 | 0.000 | 0.000 | 0.002 | 0.000 |  | 100 | S |
| *Phaeothecoidea* sp. | 0.000 | 0.000 | 0.000 | 0.004 | 0.000 | 0.000 | 0.000 | 0.000 | 0.000 | 0.000 | 0.000 | 0.000 | Capnodiales | 99 |  |
| *Phialocephala* sp. + ***P. fortinii*** + *P. fusca*  + *P. glacialis* | 0.001 | 0.000 | 0.000 | 0.029 | 0.079 | 0.005 | 0.000 | 0.015 | 0.053 | 1.033 | 0.468 | 0.627 | Helotiales | 98-100 | P |
| *Phlogicylindrium* sp. | 0.000 | 0.000 | 0.000 | 0.000 | 0.000 | 0.000 | 0.000 | 0.000 | 0.000 | 0.017 | 0.000 | 0.000 | Xylariales | 99 | S |
| *Phomatodes aubrietiae* | 0.000 | 0.000 | 0.000 | 0.000 | 0.000 | 0.000 | 0.106 | 0.000 | 0.000 | 0.000 | 0.000 | 0.000 | Pleosporales | 99 | P |
| *Phoma* sp. + ***P. boeremae*** + *P.schachtii* | 0.010 | 0.007 | 0.000 | 0.005 | 0.000 | 0.001 | 0.343 | 0.009 | 0.093 | 0.040 | 0.009 | 0.096 | Pleosporales | 98-99 | M |
| *Phomatospora dinemasporium* | 0.000 | 0.004 | 0.000 | 0.025 | 0.010 | 0.000 | 0.000 | 0.000 | 0.000 | 0.025 | 0.000 | 0.000 | Phomatosporales | 99 | S |
| *Phyllosticta kerriae* | 0.000 | 0.043 | 0.000 | 0.000 | 0.000 | 0.000 | 0.000 | 0.001 | 0.000 | 0.000 | 0.000 | 0.000 | Botryosphaeriales | 99 | S |
| *Pilidium concavum* | 0.000 | 0.000 | 0.000 | 0.035 | 0.000 | 0.000 | 0.000 | 0.001 | 0.000 | 0.000 | 0.000 | 0.004 | Chaetomellales | 97 | P |
| *Plectania melastoma* | 0.000 | 0.000 | 0.000 | 0.000 | 0.000 | 0.001 | 0.000 | 0.000 | 0.000 | 0.000 | 0.000 | 0.000 | Pezizales | 99 | S |
| *Plectosphaerella cucumerina* | 0.104 | 0.002 | 0.000 | 0.003 | 0.000 | 0.000 | 0.000 | 0.010 | 0.012 | 0.002 | 0.000 | 0.007 | Glomerellales | 99 | P |
| Pleosporaceae | 0.019 | 0.000 | 0.000 | 0.000 | 0.000 | 0.001 | 0.012 | 0.000 | 0.303 | 0.000 | 0.000 | 0.000 | Pleosporales | 100 | P |
| Pleosporales | 0.070 | 0.858 | 0.000 | 0.214 | 0.013 | 0.024 | 0.756 | 0.070 | 0.198 | 0.002 | 0.007 | 0.082 |  | 100 | P |
| *Pleotrichocladium opacum* | 1.134 | 0.636 | 0.000 | 0.229 | 0.450 | 0.028 | 0.258 | 0.523 | 0.000 | 0.107 | 0.238 | 0.172 | Pleosporales | 98 | S |
| *Pleuroascus nicholsonii* | 0.085 | 0.019 | 0.000 | 0.000 | 0.000 | 0.004 | 0.000 | 0.013 | 0.000 | 0.000 | 0.000 | 0.006 | Incertae Sedis | 99 | S |
| *Pleurophoma ossicola* | 0.000 | 0.000 | 2.080 | 0.004 | 0.000 | 0.001 | 0.020 | 0.000 | 0.124 | 0.000 | 0.000 | 0.004 | Pleosporales | 99 | S |
| *Pochonia* sp. + *P. cordycipiticonsociata* | 0.010 | 0.004 | 0.000 | 0.043 | 0.040 | 0.011 | 0.000 | 0.027 | 0.000 | 0.000 | 0.000 | 0.018 | Hypocreales | 99-100 | S |
| *Podospora* sp. + *P. appendiculata* | 0.185 | 0.032 | 0.000 | 0.000 | 0.008 | 0.000 | 0.249 | 0.013 | 0.000 | 0.000 | 0.000 | 0.000 | Sordariales | 99-100 | S |
| *Polydesmia pruinosa* | 0.000 | 0.000 | 0.000 | 0.001 | 0.000 | 0.000 | 0.000 | 0.000 | 0.000 | 0.000 | 0.000 | 0.032 | Helotiales | 99 | S |
| *Preussia persica* | 0.000 | 0.000 | 0.000 | 0.000 | 0.002 | 0.000 | 0.000 | 0.000 | 0.000 | 0.000 | 0.000 | 0.000 | Pleosporales | 97 | S |
| *Proliferodiscus* sp. | 0.000 | 0.000 | 0.000 | 0.000 | 0.000 | 0.000 | 0.585 | 0.000 | 0.337 | 0.003 | 0.016 | 0.023 | Helotiales | 99 | S |
| *Pseudallescheria boydii* | 0.031 | 0.030 | 0.000 | 0.000 | 0.000 | 0.000 | 0.000 | 0.001 | 0.000 | 0.000 | 0.000 | 0.000 | Microascales | 99 | S |
| *Pseudocamarosporium africanum* | 0.000 | 0.000 | 0.000 | 0.000 | 0.000 | 0.004 | 0.000 | 0.004 | 0.000 | 0.000 | 0.000 | 0.006 | Pleosporales | 99 | S |
| *Pseudogymnoascus verrucosus* | 0.116 | 0.013 | 0.000 | 0.427 | 0.169 | 0.062 | 0.061 | 0.272 | 0.000 | 0.461 | 1.131 | 0.213 | Incertae Sedis | 99 | S |
| *Pseudopenidiella pini* | 0.000 | 0.000 | 0.000 | 0.130 | 0.228 | 0.002 | 0.025 | 0.008 | 0.000 | 0.005 | 0.000 | 0.000 | Incertae Sedis | 99 | S |
| *Pseudoplectania nigrella* | 0.001 | 0.000 | 0.000 | 0.000 | 0.000 | 0.002 | 0.000 | 0.008 | 0.000 | 0.000 | 0.000 | 0.000 | Pezizales | 99 | S |
| *Pseudosigmoidea ibarakiensis* | 0.069 | 0.000 | 0.000 | 0.000 | 0.000 | 0.002 | 0.000 | 0.001 | 0.000 | 0.000 | 0.002 | 0.000 | Incertae Sedis | 98 | P |
| *Pustularia* sp. | 0.000 | 0.000 | 0.000 | 0.000 | 0.000 | 0.000 | 0.000 | 0.037 | 0.000 | 0.000 | 0.000 | 0.000 | Pezizales | 99 |  |
| *Pyrenochaeta* sp. | 0.002 | 0.000 | 0.000 | 0.008 | 0.000 | 0.003 | 0.000 | 0.010 | 0.000 | 0.000 | 0.000 | 0.000 | Pleosporales | 99 | P |
| *Pyrenochaetopsis leptospora* | 0.007 | 0.000 | 0.000 | 0.001 | 0.000 | 0.000 | 0.000 | 0.000 | 0.000 | 0.000 | 0.000 | 0.017 | Pleosporales | 99 | L |
| Pyronemataceae | 0.183 | 0.03 | 0.000 | 0.000 | 0.000 | 0.005 | 13.755 | 0.011 | 0.173 | 0.012 | 0.096 | 0.017 | Pezizales | 100 |  |
| *Raffaelea lauricola* | 0.000 | 0.000 | 0.000 | 0.001 | 0.000 | 0.000 | 0.000 | 0.000 | 0.000 | 0.000 | 0.000 | 0.000 | Ophiostomatales | 99 | P |
| *Ramophialophora humicola* | 0.031 | 0.009 | 0.000 | 0.004 | 0.000 | 0.000 | 0.000 | 0.003 | 0.000 | 0.000 | 0.000 | 0.000 | Sordariales | 99 |  |
| *Ramularia lactea* | 0.000 | 0.000 | 0.000 | 0.000 | 0.000 | 0.000 | 0.000 | 0.009 | 0.059 | 0.000 | 0.000 | 0.01 | Capnodiales. | 99 | S |
| *Rasamsonia* sp. | 0.006 | 0.002 | 0.000 | 0.001 | 0.000 | 0.016 | 0.000 | 0.046 | 0.000 | 0.000 | 0.000 | 0.000 | Eurotiales | 99 | S |
| *Remersonia thermophila* | 0.007 | 0.032 | 0.000 | 0.000 | 0.000 | 0.000 | 0.000 | 0.001 | 0.000 | 0.000 | 0.000 | 0.000 | Sordariales | 97 |  |
| *Remispora stellata* | 0.001 | 0.000 | 0.000 | 0.000 | 0.000 | 0.000 | 0.000 | 0.000 | 0.000 | 0.000 | 0.000 | 0.000 | Microascales | 99 | P |
| *Renispora flavissima* | 0.000 | 0.000 | 0.000 | 0.000 | 0.001 | 0.000 | 0.000 | 0.000 | 0.000 | 0.000 | 0.000 | 0.000 | Onygenales | 99 | S |
| *Rhamphoria pyriformis* | 0.000 | 0.000 | 0.000 | 0.000 | 0.000 | 0.000 | 0.000 | 0.000 | 0.000 | 0.000 | 0.000 | 0.018 | Annulatascales | 99 | S |
| *Rhexoacrodictys erecta* | 0.011 | 0.009 | 0.000 | 0.000 | 0.000 | 0.000 | 0.000 | 0.001 | 0.000 | 0.000 | 0.000 | 0.000 | Incertae Sedis | 99 | S |
| *Rhizodermea veluwensis* | 0.000 | 0.000 | 0.000 | 0.000 | 0.001 | 0.001 | 0.000 | 0.001 | 0.000 | 0.000 | 0.000 | 0.000 | Helotiales | 99 | M |
| *Rhizoscyphus* sp. | 0.001 | 0.000 | 0.000 | 0.003 | 0.000 | 0.021 | 0.114 | 0.365 | 0.133 | 0.012 | 0.341 | 2.683 | Helotiales | 99 | M |
| *Rhizosphaera kalkhoffii* | 0.000 | 0.000 | 0.000 | 0.000 | 0.000 | 0.011 | 0.000 | 0.000 | 0.000 | 0.000 | 0.000 | 0.000 | Pleosporales | 99 | P |
| *Rhodoveronaea varioseptata* | 0.009 | 0.004 | 0.000 | 0.008 | 0.000 | 0.030 | 0.221 | 0.118 | 0.000 | 0.000 | 0.037 | 0.000 | Incertae Sedis | 99 | S |
| *Rhymbocarpus neglectus* | 0.000 | 0.000 | 0.000 | 0.000 | 0.000 | 0.009 | 0.000 | 0.004 | 0.000 | 0.000 | 0.000 | 0.000 | Ostropales | 98 | L |
| Rhytismataceae | 0.000 | 0.000 | 0.000 | 0.001 | 0.000 | 0.000 | 0.339 | 0.000 | 0.000 | 0.000 | 0.000 | 0.079 | Rhytismatales | 100 | P |
| *Robillarda sessilis* | 0.000 | 0.532 | 0.000 | 0.000 | 0.000 | 0.003 | 0.000 | 0.014 | 0.000 | 0.000 | 0.000 | 0.000 | Amphisphaeriales | 99 | S |
| *Roccellaria mollis* | 0.001 | 0.013 | 0.000 | 0.009 | 0.013 | 0.000 | 0.000 | 0.003 | 0.000 | 0.000 | 0.000 | 0.000 | Arthoniales | 99 | L |
| *Saccharomycetes* sp. + *S. cerevisiae* | 0.000 | 0.000 | 0.769 | 0.000 | 0.000 | 0.000 | 0.000 | 0.006 | 0.096 | 0.002 | 0.030 | 0.000 | Saccharomycetales | 99-100 | A |
| Saccharomycetales | 0.019 | 0.000 | 0.000 | 0.000 | 0.001 | 0.042 | 0.000 | 0.028 | 0.003 | 0.000 | 0.000 | 0.000 |  | 100 | A |
| *Sagenomella diversispora*+ *S. striatispora* | 0.000 | 0.000 | 0.000 | 0.106 | 0.001 | 0.016 | 0.000 | 0.016 | 0.000 | 0.000 | 0.000 | 0.000 | Eurotiales | 99-100 | S |
| *Sarcosphaera* sp. | 0.000 | 0.000 | 0.000 | 0.018 | 0.015 | 0.000 | 0.000 | 0.000 | 0.000 | 0.000 | 0.000 | 0.000 | Pezizales | 99 | S |
| *Scedosporium dehoogii* | 0.026 | 0.026 | 0.000 | 0.000 | 0.000 | 0.002 | 0.000 | 0.003 | 0.000 | 0.000 | 0.000 | 0.000 | Microascales | 99 | S |
| *Scytalidium album* | 0.008 | 0.000 | 0.000 | 0.000 | 0.025 | 0.001 | 0.000 | 0.001 | 0.000 | 0.000 | 0.000 | 0.000 | Helotiales | 99 | P |
| *Scytalidium lignicola* | 0.000 | 0.000 | 0.000 | 0.000 | 0.000 | 0.000 | 0.000 | 0.004 | 0.000 | 0.000 | 0.000 | 0.000 | Helotiales | 99 | P |
| *Septofusidium herbarum* | 0.000 | 0.000 | 0.000 | 0.000 | 0.000 | 0.000 | 0.000 | 0.005 | 0.000 | 0.000 | 0.000 | 0.000 | Hypocreales | 98 | P |
| *Septoria lepidii* | 0.000 | 0.000 | 0.000 | 0.000 | 0.000 | 0.000 | 0.000 | 0.004 | 0.000 | 0.000 | 0.000 | 0.000 | Capnodiales | 99 | P |
| *Setomelanomma holmii* | 0.000 | 0.000 | 0.000 | 0.000 | 0.000 | 0.000 | 0.000 | 0.014 | 0.000 | 0.000 | 0.000 | 0.000 | Dothideales | 99 | P |
| *Skyttea gregaria* | 0.005 | 0.009 | 0.000 | 0.000 | 0.000 | 0.000 | 0.000 | 0.005 | 0.000 | 0.000 | 0.000 | 0.000 | Ostropales | 99 | L |
| *Sordaria fimicola* | 0.000 | 0.000 | 0.000 | 0.000 | 0.000 | 0.000 | 0.000 | 0.001 | 0.000 | 0.000 | 0.000 | 0.000 | Sordariales | 99 | S |
| Sordariales | 0.048 | 0.015 | 0.000 | 0.000 | 0.008 | 0.002 | 0.098 | 0.024 | 0.139 | 0.000 | 0.040 | 0.000 |  | 100 |  |
| Sordariomycetes | 0.292 | 0.234 | 0.000 | 0.340 | 0.126 | 0.021 | 0.008 | 0.111 | 0.006 | 0.079 | 0.100 | 0.024 |  | 100 |  |
| *Spathularia flavida* | 0.000 | 0.000 | 0.000 | 0.000 | 0.000 | 0.000 | 0.000 | 0.003 | 0.000 | 0.000 | 0.000 | 0.000 | Helotiales | 99 | S |
| *Sphaerodes fimicola* | 0.159 | 0.048 | 0.000 | 0.067 | 0.068 | 0.023 | 0.000 | 0.129 | 0.000 | 0.000 | 0.047 | 0.025 | Hypocreales | 99 | S |
| *Sphaeropsis sapinea* | 0.000 | 0.000 | 0.000 | 0.000 | 0.000 | 0.000 | 0.004 | 0.000 | 0.012 | 0.009 | 0.000 | 0.004 | Botryosphaeriales | 99 | P |
| *Sphaerostilbella aureonitens* | 0.000 | 0.011 | 4.878 | 0.000 | 0.000 | 0.000 | 0.000 | 0.006 | 0.000 | 0.000 | 0.009 | 0.000 | Hypocreales | 100 | A |
| *Spirosphaera caricigraminis* | 0.013 | 0.000 | 0.000 | 0.007 | 0.000 | 0.000 | 0.000 | 0.003 | 0.000 | 0.000 | 0.000 | 0.000 | Helotiales | 99 |  |
| *Sporopachydermia* sp. | 0.000 | 0.019 | 0.000 | 0.000 | 0.000 | 0.000 | 0.000 | 0.000 | 0.000 | 0.000 | 0.000 | 0.000 | Saccharomycetales | 99 |  |
| Sporormiaceae | 0.071 | 0.095 | 0.000 | 0.000 | 0.000 | 0.001 | 0.339 | 0.009 | 0.000 | 0.000 | 0.016 | 0.000 | Pleosporales | 100 | S |
| *Sporothrix* sp. + ***S. brunneoviolacea*** + *S. dimorphospora* | 0.085 | 0.000 | 0.000 | 0.005 | 0.000 | 0.002 | 0.061 | 0.011 | 0.000 | 0.024 | 0.040 | 0.034 | Ophiostomatales | 97-99 | P |
| *Stagonospora pseudovitensis* | 0.000 | 0.000 | 0.000 | 0.000 | 0.000 | 0.000 | 0.392 | 0.008 | 0.000 | 0.000 | 0.000 | 0.003 | Pleosporales | 99 | P |
| *Stagonosporopsis* sp. | 0.000 | 0.035 | 0.000 | 0.000 | 0.000 | 0.000 | 0.000 | 0.003 | 0.000 | 0.000 | 0.000 | 0.000 | Pleosporales | 99 | P |
| Stictidaceae | 0.000 | 0.000 | 0.000 | 0.000 | 0.000 | 0.000 | 0.000 | 0.000 | 0.000 | 0.000 | 0.000 | 0.004 | Ostropales | 100 |  |
| *Strumella* sp. | 0.000 | 0.000 | 0.000 | 0.000 | 0.000 | 0.000 | 0.736 | 0.005 | 0.000 | 0.010 | 0.000 | 0.025 | Pezizales | 99 | S |
| *Sugiyamaella pinicola* | 0.000 | 0.000 | 0.000 | 0.000 | 0.015 | 0.000 | 0.000 | 0.000 | 0.000 | 0.000 | 0.000 | 0.000 | Saccharomycetales | 99 | A |
| *Suhomyces vadensis* | 0.009 | 0.007 | 0.000 | 0.000 | 0.000 | 0.000 | 0.000 | 0.000 | 0.000 | 0.000 | 0.000 | 0.000 | Saccharomycetales | 99 | A |
| *Sympodiella acicola* | 0.000 | 0.000 | 0.000 | 0.000 | 0.000 | 0.000 | 0.000 | 0.000 | 0.000 | 0.347 | 0.000 | 0.000 | Incertae Sedis | 99 | S |
| Sympoventuriaceae | 0.000 | 0.000 | 0.000 | 0.013 | 0.003 | 0.017 | 0.000 | 0.001 | 0.009 | 0.000 | 0.023 | 0.018 | Venturiales | 100 |  |
| *Talaromyces* sp. + *T. verruculosus* + *T. stollii*  + *T. verruculosus* | 0.000 | 0.000 | 0.000 | 0.000 | 0.000 | 0.000 | 0.000 | 0.034 | 0.012 | 0.000 | 0.000 | 0.000 | Eurotiales | 98-100 | S |
| *Taphrina carpini* + *T. tormentillae* | 0.021 | 0.015 | 0.000 | 0.001 | 0.012 | 0.002 | 0.000 | 0.005 | 0.000 | 0.000 | 0.000 | 0.011 | Taphrinales | 99-100 | P |
| *Teratosphaeria mexicana* | 0.000 | 0.000 | 0.000 | 0.000 | 0.000 | 0.001 | 0.000 | 0.000 | 0.000 | 0.000 | 0.000 | 0.011 | Capnodiales | 99 | P |
| *Terfezia claveryi* | 0.000 | 0.000 | 0.000 | 0.018 | 0.009 | 0.000 | 0.000 | 0.000 | 0.000 | 0.000 | 0.000 | 0.000 | Pezizales | 99 | M |
| *Terriera illiciicola* | 0.000 | 0.000 | 0.000 | 0.005 | 0.000 | 0.000 | 0.000 | 0.000 | 0.000 | 0.000 | 0.000 | 0.000 | Rhytismatales | 99 |  |
| ***Tetracladium* sp.** + *T. setigerum* | 0.810 | 0.229 | 0.000 | 0.004 | 0.005 | 0.013 | 0.691 | 0.071 | 0.182 | 0.000 | 0.021 | 0.008 | Helotiales | 99-100 | S |
| *Thermomyces dupontii* + *T. lanuginosus* | 0.017 | 0.002 | 0.000 | 0.000 | 0.000 | 0.000 | 0.000 | 0.000 | 0.000 | 0.000 | 0.000 | 0.000 | Eurotiales | 99-100 | A |
| *Tolypocladium* sp. | 0.000 | 0.000 | 0.000 | 0.000 | 0.005 | 0.015 | 0.000 | 0.001 | 0.000 | 0.000 | 0.000 | 0.000 | Hypocreales | 99 | E |
| Trichocomaceae | 0.000 | 0.000 | 0.000 | 0.114 | 0.001 | 0.011 | 0.000 | 0.019 | 0.195 | 0.000 | 0.000 | 0.001 | Eurotiales | 100 | A |
| *Trichoderma* sp. + *T. alcalifuscescens*  + *T. asperellum* + *T. crassum* + *T. cremeum*  + *T. foliicola* + *T. fomiticola* + *T. longipilis*  + *T. martiale* + *T. neokoningii* + *T. nothescens* + *T. pachypallidum* + *T. parapiluliferum*  + *T. pleuroti* + *T. pubescens* + *T. rhododendri*  + *T. rossicum* + *T. spirale* + *T. stellatum*  + *T. theobromicola* | 0.170 | 0.082 | 2.793 | 0.468 | 0.162 | 0.130 | 0.200 | 0.216 | 0.266 | 0.037 | 0.009 | 0.054 | Eurotiales | 97-100 | A |
| *Trichophaea hybrida* | 0.001 | 0.000 | 0.000 | 0.000 | 0.000 | 0.000 | 0.000 | 0.005 | 0.000 | 0.000 | 0.000 | 0.000 | Pezizales | 99 | A |
| *Truncatella restionacearum* | 0.000 | 0.000 | 0.000 | 0.000 | 0.000 | 0.000 | 0.000 | 0.001 | 0.000 | 0.016 | 0.000 | 0.000 | Amphisphaeriales | 99 | S |
| *Tuber* sp. + ***T. anniae*** + *T. borchii* + *T. cistophilum* | 0.05 | 0.205 | 0.000 | 0.000 | 0.000 | 0.002 | 1.700 | 11.136 | 0.043 | 0.007 | 32.279 | 0.035 | Pezizales | 98-100 | M |
| *Tumularia* sp. | 0.000 | 0.000 | 0.000 | 0.000 | 0.000 | 0.000 | 0.000 | 0.000 | 0.096 | 0.000 | 0.000 | 0.000 | Incertae Sedis | 98 | S |
| *Umbilicaria americana* | 0.000 | 0.004 | 0.000 | 0.000 | 0.000 | 0.000 | 0.000 | 0.000 | 0.266 | 0.000 | 0.030 | 0.000 | Umbilicariales | 99 | L |
| *Valdensinia heterodoxa* | 0.000 | 0.000 | 0.000 | 0.075 | 0.000 | 0.000 | 0.000 | 0.000 | 0.000 | 0.000 | 0.000 | 0.000 | Helotiales | 99 | P |
| *Valsa abietis* | 0.000 | 0.000 | 0.035 | 0.000 | 0.001 | 0.000 | 0.025 | 0.000 | 0.046 | 0.002 | 0.000 | 0.003 | Diaporthales | 99 | P |
| Valsaceae | 0.000 | 0.000 | 0.01 | 0.000 | 0.000 | 0.000 | 0.102 | 0.004 | 0.031 | 0.005 | 0.000 | 0.027 | Diaporthales | 100 | P |
| *Venturia* sp. + ***V. hystrioides*** | 0.014 | 0.013 | 0.000 | 0.361 | 0.080 | 0.004 | 1.197 | 0.028 | 0.000 | 0.149 | 0.009 | 0.062 | Venturiales | 99-100 | P |
| Venturiaceae | 0.181 | 0.004 | 0.000 | 0.115 | 0.051 | 0.052 | 0.131 | 0.087 | 0.028 | 0.143 | 0.091 | 0.001 | Venturiales | 100 | P |
| Venturiales | 0.000 | 0.000 | 0.000 | 0.000 | 0.000 | 0.000 | 0.000 | 0.001 | 0.000 | 0.000 | 0.000 | 0.000 |  | 100 | P |
| *Veronaeopsis* sp. | 0.000 | 0.000 | 0.000 | 0.000 | 0.000 | 0.000 | 0.000 | 0.001 | 0.000 | 0.000 | 0.000 | 0.000 | Pleosporales | 99 | A |
| *Verrucaria* sp. + *V. elaeomelaena* | 0.005 | 0.048 | 0.000 | 0.045 | 0.002 | 0.001 | 0.000 | 0.000 | 0.000 | 0.000 | 0.000 | 0.000 | Verrucariales | 99-100 | L |
| Verrucariaceae | 0.000 | 0.000 | 0.000 | 0.000 | 0.000 | 0.000 | 0.000 | 0.000 | 0.000 | 0.000 | 0.000 | 0.000 | Verrucariales | 100 | L |
| *Verticillium* sp. + *V. leptobactrum* | 0.001 | 0.000 | 0.000 | 0.000 | 0.000 | 0.058 | 0.000 | 0.003 | 0.000 | 0.000 | 0.000 | 0.000 | Glomerellales | 99-100 | P |
| *Vestigium* sp. | 0.000 | 0.000 | 0.000 | 0.000 | 0.000 | 0.000 | 0.098 | 0.000 | 0.056 | 0.002 | 0.000 | 0.070 | Incertae Sedis | 99 | S |
| *Volutella* sp. | 0.000 | 0.000 | 0.000 | 0.014 | 0.013 | 0.000 | 0.000 | 0.005 | 0.000 | 0.000 | 0.000 | 0.000 | Hypocreales | 99 | P |
| *Westerdykella multispora* | 0.002 | 0.015 | 0.000 | 0.000 | 0.000 | 0.000 | 0.000 | 0.000 | 0.000 | 0.000 | 0.000 | 0.000 | Pleosporales | 99 | A |
| *Wilcoxina* sp. + *W. rehmii* | 0.000 | 0.006 | 0.000 | 0.000 | 0.003 | 0.000 | 0.008 | 0.018 | 0.000 | 0.174 | 0.061 | 0.032 | Pezizales | 99-100 | M |
| *Xanthoparmelia hottentotta* | 0.001 | 0.004 | 0.000 | 0.000 | 0.000 | 0.000 | 0.000 | 0.006 | 0.000 | 0.000 | 0.000 | 0.000 | Lecanorales | 99 | L |
| *Xenochalara* sp. | 0.001 | 0.000 | 0.000 | 0.009 | 0.000 | 0.001 | 0.000 | 0.001 | 0.000 | 0.002 | 0.000 | 0.000 | Incertae Sedis | 99 | P |
| *Xenopolyscytalum pinea* | 0.224 | 0.011 | 0.000 | 0.018 | 0.033 | 0.002 | 0.102 | 0.034 | 0.000 | 0.117 | 1.903 | 0.278 | Helotiales | 99 | P |
| Xylariaceae | 0.000 | 0.000 | 0.000 | 0.000 | 0.000 | 0.002 | 0.000 | 0.001 | 0.000 | 0.000 | 0.000 | 0.000 | Xylariales | 100 | S |
| Xylariales | 0.046 | 0.041 | 0.000 | 0.022 | 0.009 | 0.003 | 0.000 | 0.006 | 0.000 | 0.000 | 0.000 | 0.000 |  | 100 | S |
| *Yarrowia lipolytica* | 0.001 | 0.000 | 0.000 | 0.000 | 0.000 | 0.000 | 0.000 | 0.000 | 0.000 | 0.000 | 0.000 | 0.000 | Saccharomycetales | 99 | A |
| *Zignoella* sp. | 0.000 | 0.000 | 0.000 | 0.000 | 0.000 | 0.000 | 0.000 | 0.000 | 0.056 | 0.000 | 0.000 | 0.000 | Chaetosphaeriales | 99 | P |
| *Zopfiella marina* | 0.000 | 0.032 | 0.000 | 0.000 | 0.000 | 0.000 | 0.000 | 0.001 | 0.000 | 0.000 | 0.000 | 0.000 | Sordariales | 99 |  |
| *Zymoseptoria verkleyi* | 0.000 | 0.000 | 0.000 | 0.01 | 0.008 | 0.000 | 0.000 | 0.000 | 0.000 | 0.000 | 0.000 | 0.000 | Capnodiales | 99 | P |
| **Frequency of Ascomycota** | 17.073 | 9.174 | 10.777 | 29.681 | 11.758 | 11.270 | 59.979 | 36.051 | 31.514 | 30.589 | 60.41 | 37.277 |  |  |  |
| **Basidiomycota** | | | | | | | | | | | | | | | |
| *Aecidium* sp. + *Aecidium guatteriae* | 0.002 | 0.000 | 0.000 | 0.005 | 0.012 | 0.002 | 0.000 | 0.004 | 0.000 | 0.000 | 0.000 | 0.000 | Pucciniales | 99-100 | P |
| Agaricales | 1.038 | 1.477 | 0.216 | 0.334 | 1.670 | 0.116 | 0.147 | 2.482 | 1.930 | 4.879 | 0.441 | 1.856 |  | 100 |  |
| *Agaricomycetes* sp. | 0.042 | 0.115 | 0.000 | 0.077 | 0.092 | 0.367 | 0.000 | 0.204 | 0.003 | 0.000 | 0.023 | 0.035 | Agaricales | 99 |  |
| *Agaricus sylvaticus* | 0.001 | 0.000 | 0.000 | 0.000 | 0.000 | 0.000 | 0.000 | 0.003 | 0.000 | 0.000 | 0.000 | 0.000 | Agaricales | 99 | S |
| *Agrocybe arvalis* + *A. praecox* | 0.000 | 0.000 | 0.000 | 0.081 | 0.011 | 0.003 | 0.000 | 0.000 | 0.000 | 0.003 | 0.000 | 0.000 | Agaricales | 99-100 | S |
| *Amanita* sp. + *A. beckeri* + *A. fulva*  + *A. gemmata* + *A. muscaria* + ***A. rubescens*** | 0.058 | 0.035 | 0.000 | 0.009 | 0.178 | 7.809 | 0.000 | 0.523 | 0.198 | 0.000 | 0.000 | 0.018 | Agaricales | 98-100 | M |
| *Amphinem*a sp. + ***A. byssoides*** | 0.001 | 0.000 | 0.000 | 0.000 | 0.000 | 0.000 | 0.000 | 0.004 | 0.000 | 0.000 | 10.341 | 0.024 | Atheliales | 99-100 | S |
| *Amphistereum leveilleanum* | 0.000 | 0.000 | 0.000 | 0.000 | 0.000 | 0.000 | 0.208 | 0.033 | 0.000 | 0.098 | 0.105 | 0.316 | Auriculariales | 99 | P |
| *Amylostereum laevigatum* | 0.000 | 0.000 | 0.000 | 0.000 | 0.000 | 0.000 | 0.000 | 0.003 | 0.000 | 0.000 | 0.000 | 0.000 | Russulales | 99 | S |
| *Anomoloma* sp. | 0.001 | 0.000 | 0.000 | 0.000 | 0.000 | 0.000 | 0.000 | 0.000 | 0.000 | 0.000 | 0.000 | 0.000 | Agaricales | 99 | S |
| *Apiotrichum dulcitum* + *A. laibachii* | 0.034 | 0.000 | 0.000 | 0.088 | 0.000 | 0.000 | 0.000 | 0.002 | 0.000 | 0.000 | 0.000 | 0.000 | Trichosporonales | 99-100 | S |
| *Athelia epiphylla* | 0.000 | 0.000 | 0.000 | 0.000 | 0.000 | 0.000 | 0.000 | 0.001 | 0.000 | 0.010 | 0.000 | 0.000 | Atheliales | 99 | S |
| *Athelia* sp. | 0.000 | 0.000 | 0.000 | 0.000 | 0.000 | 0.000 | 0.000 | 0.001 | 0.000 | 0.000 | 0.000 | 0.003 | Atheliales | 99 | S |
| Atheliaceae | 0.202 | 0.143 | 0.000 | 0.000 | 0.000 | 0.010 | 0.724 | 0.018 | 0.000 | 0.014 | 0.014 | 0.051 | Atheliales | 100 |  |
| Atheliales | 0.000 | 0.000 | 0.000 | 0.000 | 0.000 | 0.011 | 0.000 | 0.016 | 0.000 | 0.000 | 1.269 | 0.000 |  | 100 |  |
| *Athelopsis lembospora* | 0.000 | 0.000 | 0.000 | 0.000 | 0.000 | 0.009 | 0.000 | 0.001 | 3.616 | 3.348 | 0.000 | 0.082 | Atheliales | 99 | S |
| Auriculariales | 0.002 | 0.002 | 0.000 | 0.876 | 0.003 | 0.035 | 0.000 | 0.006 | 0.025 | 0.012 | 2.113 | 0.617 |  | 100 |  |
| *Basidiodendron caesiocinereum* | 0.000 | 0.000 | 0.000 | 0.009 | 0.000 | 0.000 | 0.000 | 0.179 | 0.000 | 0.247 | 0.016 | 0.008 | Auriculariales | 99 | S |
| Basidiomycota | 0.722 | 0.277 | 0.000 | 1.900 | 0.467 | 0.431 | 0.417 | 1.471 | 0.371 | 0.423 | 0.009 | 0.147 |  | 100 |  |
| *Bensingtonia* sp. + *B. bomiensis* | 0.026 | 0.017 | 0.000 | 0.004 | 0.000 | 0.000 | 0.000 | 0.005 | 0.000 | 0.000 | 0.000 | 0.000 | Agaricostilbales | 99-100 | S |
| *Biatoropsis usnearum* | 0.000 | 0.000 | 0.000 | 0.013 | 0.011 | 0.001 | 0.000 | 0.000 | 0.000 | 0.000 | 0.000 | 0.006 | Tremellales | 99 | L |
| Boletaceae | 0.001 | 0.000 | 0.000 | 0.000 | 0.005 | 0.047 | 0.000 | 0.000 | 0.000 | 0.000 | 0.000 | 0.000 | Boletales | 100 | M |
| Boletales | 0.000 | 0.000 | 0.000 | 0.000 | 0.000 | 0.000 | 0.000 | 0.001 | 0.000 | 0.000 | 0.000 | 0.000 |  | 100 | M |
| *Boletus edulis* | 0.003 | 0.002 | 0.000 | 0.000 | 0.082 | 0.365 | 0.000 | 0.027 | 0.000 | 0.009 | 0.000 | 0.000 | Boletales | 98 | M |
| *Botryobasidium laeve* | 0.000 | 0.000 | 0.000 | 0.000 | 0.017 | 0.001 | 0.000 | 0.001 | 0.000 | 0.091 | 0.000 | 0.048 | Cantharellales | 99 | S |
| *Buckleyzyma aurantiaca* | 0.000 | 0.000 | 0.000 | 0.000 | 0.000 | 0.000 | 0.131 | 0.000 | 0.000 | 0.007 | 0.000 | 0.000 | Buckleyzymales | 99 | A |
| *Camarophyllus* sp. | 0.003 | 0.002 | 0.000 | 0.000 | 0.002 | 0.007 | 0.000 | 0.001 | 0.000 | 0.000 | 0.000 | 0.000 | Agaricales | 99 | M |
| Cantharellales | 0.449 | 0.409 | 0.000 | 0.000 | 0.000 | 0.173 | 0.033 | 0.043 | 0.659 | 0.016 | 0.005 | 0.041 |  | 100 | M |
| *Cantharellus decolorans* | 0.062 | 0.125 | 0.000 | 0.087 | 0.268 | 0.011 | 0.000 | 0.053 | 0.000 | 0.005 | 0.056 | 0.021 | Cantharellales | 99 | M |
| Ceratobasidiaceae | 0.000 | 0.000 | 0.000 | 0.173 | 0.000 | 0.000 | 0.098 | 0.070 | 0.074 | 0.002 | 0.336 | 1.171 | Cantharellales | 100 | S |
| *Ceratobasidium* sp. | 0.000 | 0.000 | 0.000 | 0.000 | 0.000 | 0.005 | 0.000 | 0.015 | 0.000 | 0.000 | 0.002 | 0.000 | Cantharellales | 99 | S |
| *Chalciporus* sp. | 0.010 | 0.000 | 0.000 | 0.000 | 0.000 | 0.000 | 0.000 | 0.000 | 0.000 | 0.000 | 0.000 | 0.000 | Boletales | 99 | M |
| *Chlorophyllum hortense* | 0.002 | 0.000 | 0.000 | 0.000 | 0.000 | 0.000 | 0.000 | 0.000 | 0.000 | 0.000 | 0.000 | 0.000 | Agaricales | 99 | S |
| *Clavaria* sp. + *C. amoenoides* + *C. argillacea* | 0.002 | 0.006 | 0.000 | 0.007 | 0.005 | 0.000 | 0.000 | 0.013 | 0.000 | 0.000 | 0.000 | 0.000 | Agaricales | 98-99 | S |
| Clavariaceae | 0.095 | 0.033 | 0.000 | 0.001 | 0.000 | 0.119 | 0.000 | 0.563 | 0.102 | 0.000 | 0.000 | 0.000 | Agaricales | 100 | S |
| *Clavulina* sp. + ***C. coralloides*** | 0.017 | 0.002 | 0.000 | 0.010 | 0.100 | 1.760 | 0.000 | 0.104 | 2.094 | 0.000 | 0.000 | 0.000 | Cantharellales | 99-100 |  |
| *Clavulinopsis* sp. | 0.061 | 0.013 | 0.000 | 0.000 | 0.000 | 0.081 | 0.000 | 0.300 | 0.000 | 0.000 | 0.000 | 0.000 | Agaricales | 99 | S |
| *Clinoconidium* sp. | 0.007 | 0.000 | 0.000 | 0.014 | 0.003 | 0.000 | 0.000 | 0.000 | 0.000 | 0.000 | 0.000 | 0.000 | Exobasidiales | 99 | P |
| *Clitocybe nebularis* | 0.000 | 0.002 | 0.000 | 0.000 | 0.000 | 0.000 | 0.000 | 0.000 | 0.000 | 0.000 | 0.000 | 0.000 | Agaricales | 99 | S |
| *Colacogloea* sp. | 0.000 | 0.000 | 0.000 | 0.000 | 0.000 | 0.002 | 0.000 | 0.008 | 0.000 | 0.000 | 0.000 | 0.000 | Heterogastridiales | 99 | P |
| *Conocybe echinata* + *C. semiglobata* | 0.000 | 0.000 | 0.000 | 0.142 | 0.018 | 0.000 | 0.000 | 0.000 | 0.000 | 0.000 | 0.000 | 0.003 | Agaricales | 99-100 | M |
| *Coprinellus micaceus* + *C. verrucispermus* | 0.018 | 0.009 | 0.000 | 0.000 | 0.000 | 0.000 | 0.000 | 0.003 | 0.161 | 0.000 | 0.000 | 0.000 | Agaricales | 99-100 | S |
| *Coprinopsis narcotica* | 0.047 | 0.054 | 0.000 | 0.000 | 0.000 | 0.002 | 0.000 | 0.003 | 0.000 | 0.000 | 0.000 | 0.000 | Agaricales | 99 | S |
| *Corticium confine* | 0.000 | 0.000 | 0.000 | 0.000 | 0.000 | 0.000 | 0.000 | 0.000 | 0.269 | 0.000 | 0.000 | 0.000 | Corticiales | 99 | M |
| Corticiaceae | 0.016 | 0.000 | 0.000 | 0.000 | 0.000 | 0.000 | 0.000 | 0.003 | 0.000 | 0.000 | 0.000 | 0.000 | Corticiales | 100 | M |
| Cortinariaceae | 0.013 | 0.000 | 0.000 | 0.000 | 0.000 | 0.000 | 0.000 | 0.003 | 0.000 | 0.000 | 0.000 | 0.000 | Corticiales | 100 | M |
| *Cortinarius* sp.+ *C. croceus* + *C. fulvoconicus*  + *C. semisanguineus* | 0.006 | 0.000 | 0.000 | 0.03 | 0.004 | 0.188 | 0.000 | 0.014 | 0.26 | 0.003 | 0.000 | 0.000 | Corticiales | 98-100 | M |
| *Cronartium pini* | 0.002 | 0.000 | 0.000 | 0.000 | 0.012 | 0.152 | 0.000 | 0.014 | 0.000 | 0.000 | 0.000 | 0.001 | Corticiales | 99 | P |
| *Cryptococcus* sp. + *C. neoformans*  + *C. psychrotolerans* + C. pseudolongus | 0.000 | 0.000 | 0.000 | 0.028 | 0.017 | 0.002 | 0.000 | 0.144 | 0.074 | 0.005 | 0.000 | 0.045 | Tremellales | 98-100 | S |
| *Curvibasidium cygneicollum* | 0.000 | 0.000 | 0.000 | 0.003 | 0.003 | 0.000 | 0.556 | 0.003 | 0.155 | 0.000 | 0.000 | 0.014 | Microbotryales | 99 | P |
| *Cutaneotrichosporon moniliiforme* | 0.002 | 0.000 | 0.000 | 0.000 | 0.000 | 0.000 | 0.000 | 0.003 | 0.000 | 0.000 | 0.030 | 0.014 | Trichosporonales | 99 | S |
| *Cystobasidiopsis lophatheri* | 0.001 | 0.000 | 0.000 | 0.000 | 0.000 | 0.000 | 0.000 | 0.000 | 0.000 | 0.000 | 0.000 | 0.000 | Agaricostilbales | 99 |  |
| *Cystobasidium psychroaquaticum* | 0.000 | 0.011 | 0.000 | 0.000 | 0.000 | 0.000 | 0.000 | 0.000 | 0.000 | 0.000 | 0.000 | 0.007 | Cystobasidiales | 99 | P |
| Cystofilobasidiales | 0.000 | 0.000 | 0.000 | 0.000 | 0.001 | 0.002 | 0.000 | 0.000 | 0.000 | 0.003 | 0.107 | 0.004 |  | 100 | P |
| *Cystofilobasidium macerans* | 0.011 | 0.000 | 0.000 | 0.000 | 0.000 | 0.000 | 0.000 | 0.000 | 0.000 | 0.000 | 0.000 | 0.000 | Cystofilobasidiales | 99 | P |
| Daedaleopsis confragosa | 0.000 | 0.000 | 0.000 | 0.000 | 0.000 | 0.000 | 0.000 | 0.001 | 0.000 | 0.000 | 0.000 | 0.000 | Polyporales | 99 | P |
| *Deconica* | 0.002 | 0.033 | 0.000 | 0.005 | 0.018 | 0.004 | 0.000 | 0.001 | 0.557 | 0.005 | 0.012 | 0.000 | Agaricales | 100 | S |
| Derxomyces | 0.023 | 0.007 | 0.000 | 0.000 | 0.000 | 0.000 | 0.000 | 0.003 | 0.000 | 0.000 | 0.000 | 0.000 | Tremellales | 100 | P |
| *Dioszegia changbaiensis* + *D. hungarica* | 0.003 | 0.000 | 0.000 | 0.000 | 0.000 | 0.001 | 0.000 | 0.013 | 0.071 | 0.000 | 0.000 | 0.000 | Tremellales | 99-100 | P |
| *Exophiala byssisedum* + *E. conferendum*  + *E. gelatinosum* + *E. juncinum* + *E. kerocarpus* + *E. kristiansenii* *+ E. lanicum* + *E. lepidissimum* + *E. sericeum* | 0.024 | 0.000 | 0.000 | 0.022 | 0.064 | 0.005 | 0.000 | 0.036 | 0.000 | 0.000 | 0.000 | 0.000 | Chaetothyriales | 98-100 | S |
| Entolomataceae | 0.000 | 0.000 | 0.000 | 0.001 | 0.000 | 0.000 | 0.000 | 0.001 | 0.000 | 0.000 | 0.000 | 0.000 | Agaricales | 100 | S |
| Erythrobasidiales | 0.007 | 0.074 | 0.000 | 0.000 | 0.002 | 0.000 | 0.000 | 0.009 | 0.000 | 0.000 | 0.000 | 0.000 |  | 100 |  |
| *Erythrobasidium hasegawae* | 0.000 | 0.000 | 0.000 | 0.000 | 0.000 | 0.000 | 0.000 | 0.001 | 0.000 | 0.000 | 0.000 | 0.007 | Erythrobasidiales | 99 | E |
| *Exobasidium maculosum*  + *E. pieridisovalifoliae* | 0.000 | 0.000 | 0.000 | 0.000 | 0.000 | 0.000 | 0.000 | 0.001 | 0.000 | 0.000 | 0.000 | 0.001 | Exobasidiales | 99-100 | P |
| *Fellozyma inositophila* | 0.013 | 0.000 | 0.000 | 0.000 | 0.000 | 0.000 | 0.000 | 0.003 | 0.000 | 0.000 | 0.000 | 0.015 | Incertae Sedis | 99 | S |
| *Fibulobasidium* | 0.000 | 0.000 | 0.000 | 0.003 | 0.003 | 0.000 | 0.000 | 0.001 | 0.000 | 0.000 | 0.000 | 0.000 | Tremellales | 100 | P |
| *Filobasidium stepposum* + *F. wieringae* | 0.000 | 0.000 | 0.000 | 0.000 | 0.000 | 0.000 | 0.000 | 0.001 | 0.000 | 0.005 | 0.000 | 0.004 | Filobasidiales | 99-100 | S |
| *Flagelloscypha minutissima* | 0.222 | 0.000 | 0.000 | 0.000 | 0.000 | 0.001 | 0.000 | 0.018 | 0.000 | 0.000 | 0.019 | 0.000 | Agaricales | 99 | S |
| *Galerina* sp. + ***G. atkinsoniana*** + *G. fallax*  + *G. nana* + *G. pseudocamerina* + ***G. stylifera*** | 0.018 | 0.015 | 0.000 | 2.148 | 0.013 | 2.132 | 0.000 | 0.364 | 2.221 | 0.000 | 0.017 | 0.082 | Agaricales | 98-100 | S |
| *Geastrum* sp. + *G. striatum* | 0.000 | 0.000 | 0.000 | 0.000 | 0.000 | 0.000 | 0.000 | 0.009 | 0.000 | 0.002 | 0.000 | 0.000 | Geastrales | 99-100 | S |
| *Gelidatrema spencermartinsiae* | 0.000 | 0.000 | 0.000 | 0.000 | 0.000 | 0.000 | 0.102 | 0.000 | 0.000 | 0.000 | 0.000 | 0.004 | Tremellales | 99 | S |
| *Geminibasidium* sp. | 0.006 | 0.002 | 0.000 | 0.283 | 0.001 | 0.016 | 0.000 | 0.072 | 0.059 | 0.000 | 0.000 | 0.008 | Geminibasidiales | 98 |  |
| *Goffeauzyma aciditolerans* + *G. gastrica* | 0.003 | 0.000 | 0.000 | 0.02 | 0.008 | 0.001 | 0.000 | 0.004 | 0.022 | 0.000 | 0.000 | 0.000 | Filobasidiales | 99-100 | S |
| *Gymnopilus decipiens* + *G. penetrans* | 0.014 | 0.000 | 0.000 | 0.000 | 0.000 | 0.568 | 0.000 | 0.037 | 0.000 | 0.000 | 0.000 | 0.000 | Agaricales | 99-100 | S |
| *Gymnopus* sp. + *G. androsaceus* | 0.000 | 0.000 | 0.000 | 0.000 | 0.007 | 0.009 | 0.000 | 0.002 | 0.000 | 0.000 | 0.000 | 0.000 | Agaricales | 99-100 | A |
| *Hebeloma cavipes* | 0.005 | 0.019 | 0.000 | 0.000 | 0.005 | 0.000 | 0.000 | 0.001 | 0.000 | 0.000 | 0.000 | 0.000 | Agaricales | 99 | M |
| Helicobasidiaceae | 0.000 | 0.000 | 0.000 | 0.000 | 0.000 | 0.000 | 0.000 | 0.003 | 0.000 | 0.000 | 0.000 | 0.000 | Helicobasidiales | 100 |  |
| *Henningsomyces candidus* | 0.003 | 0.000 | 0.000 | 0.001 | 0.000 | 0.002 | 0.000 | 0.002 | 0.000 | 0.000 | 0.000 | 0.001 | Agaricales | 99 | S |
| Hydnaceae | 0.000 | 0.007 | 0.01 | 0.000 | 0.000 | 0.000 | 0.004 | 0.160 | 1.512 | 0.005 | 0.435 | 0.004 | Cantharellales | 100 | M |
| Hydnodontaceae | 0.003 | 0.000 | 0.025 | 0.000 | 0.001 | 0.000 | 0.004 | 0.005 | 0.000 | 0.003 | 0.000 | 0.008 | Trechisporales | 100 | M |
| *Hydnomerulius pinastri* | 0.000 | 0.000 | 0.000 | 0.004 | 0.000 | 0.000 | 0.000 | 0.000 | 0.000 | 0.000 | 0.000 | 0.000 | Boletales | 99 | S |
| *Hydnum* sp. | 0.001 | 0.002 | 0.000 | 0.000 | 0.000 | 0.004 | 0.000 | 0.004 | 0.000 | 0.000 | 0.000 | 0.000 | Cantharellales | 99 | M |
| *Hygrocybe* sp. + *H. appalachianensis*  + *H. intermedia* | 0.006 | 0.000 | 0.000 | 0.016 | 0.209 | 0.009 | 0.000 | 0.013 | 0.000 | 0.000 | 0.000 | 0.000 | Agaricales | 98-99 | M |
| Hygrophoraceae | 0.158 | 0.154 | 0.000 | 0.615 | 0.467 | 0.401 | 0.000 | 0.245 | 0.139 | 1.182 | 2.689 | 6.384 | Agaricales | 100 | M |
| *Hygrophoropsis aurantiaca* | 0.001 | 0.000 | 0.000 | 0.000 | 0.000 | 0.000 | 0.000 | 0.001 | 0.000 | 0.000 | 0.000 | 0.000 | Boletales | 99 | S |
| *Hygrophorus hypothejus* | 0.001 | 0.000 | 0.000 | 0.000 | 0.000 | 0.007 | 0.000 | 0.01 | 0.000 | 0.000 | 0.000 | 0.000 | Agaricales | 99 | M |
| Hymenochaetales | 0.000 | 0.000 | 0.000 | 0.004 | 0.008 | 0.000 | 0.000 | 0.142 | 0.213 | 0.009 | 0.175 | 0.000 |  | 100 |  |
| *Hymenogaster boozeri* + ***H. huthii*** +  *H. olivaceus* | 0.254 | 0.011 | 0.000 | 0.069 | 0.015 | 0.002 | 0.707 | 0.024 | 0.000 | 0.041 | 0.009 | 6.731 | Agaricales | 98-100 | M |
| *Hyphodontiella multiseptata* | 0.000 | 0.000 | 0.000 | 0.000 | 0.000 | 0.000 | 0.000 | 0.004 | 0.000 | 0.000 | 0.000 | 0.000 | Agaricales | 99 | P |
| *Hyphodontia pallidula* | 0.000 | 0.000 | 0.000 | 0.001 | 0.000 | 0.000 | 0.000 | 0.004 | 0.000 | 0.000 | 0.000 | 0.000 | Hymenochaetales | 99 | S |
| *Hypholoma fasciculare* | 0.000 | 0.000 | 0.015 | 0.000 | 0.000 | 0.000 | 0.000 | 0.000 | 0.003 | 0.000 | 0.000 | 0.000 | Agaricales | 99 | S |
| *Hypochnicium* sp. + *H. geogenium*  + *H. punctulatum* | 0.000 | 0.020 | 0.000 | 0.000 | 0.072 | 0.000 | 0.000 | 0.000 | 0.000 | 0.01 | 0.000 | 0.000 | Polyporales | 98-99 | S |
| Inocybaceae | 0.000 | 0.000 | 0.000 | 0.029 | 0.000 | 0.000 | 0.000 | 0.000 | 0.000 | 0.000 | 0.000 | 0.000 | Agaricales | 100 | S |
| *Inocybe* sp.+ *I. curvipes* + *I. flocculosa* + *I. napipes* + *I. nitidiuscula* + *I. rimosa*  + ***I. rufoalba*** | 0.046 | 0.755 | 0.000 | 0.002 | 0.292 | 0.783 | 0.000 | 2.461 | 0.043 | 0.259 | 0.341 | 0.001 | Agaricales | 98-100 | M |
| *Itersonilia pannonica* + ***I. perplexans*** | 0.000 | 0.000 | 0.000 | 0.000 | 0.000 | 0.000 | 0.343 | 0.000 | 0.201 | 0.022 | 0.000 | 0.045 | Cystofilobasidiales | 99-100 |  |
| *Kockovaella sacchari* | 0.000 | 0.000 | 0.000 | 0.000 | 0.000 | 0.000 | 0.000 | 0.000 | 0.306 | 0.000 | 0.000 | 0.000 | Tremellales | 99 | S |
| *Kondoa phyllada* | 0.000 | 0.000 | 0.000 | 0.000 | 0.000 | 0.000 | 0.000 | 0.003 | 0.000 | 0.000 | 0.000 | 0.000 | Agaricostilbales | 99 |  |
| *Kurtzmanomyces* sp. | 0.000 | 0.000 | 0.000 | 0.000 | 0.000 | 0.000 | 0.000 | 0.011 | 0.000 | 0.000 | 0.000 | 0.000 | Agaricostilbales | 99 | P |
| *Kwoniella pini* | 0.000 | 0.000 | 0.000 | 0.000 | 0.000 | 0.000 | 0.033 | 0.000 | 0.000 | 0.000 | 0.000 | 0.027 | Tremellales | 99 | S |
| *Lactarius musteus* + *L. necator* + *L. quietus*  + *L. rufus* + ***L. tabidus*** | 0.042 | 0.019 | 0.000 | 0.000 | 0.06 | 3.081 | 0.008 | 0.206 | 7.074 | 0.000 | 0.000 | 0.000 | Russulales | 98-100 | M |
| *Laetiporus* sp. | 0.000 | 0.000 | 0.000 | 0.000 | 0.000 | 0.000 | 0.000 | 0.001 | 0.000 | 0.000 | 0.000 | 0.010 | Polyporales | 99 | P |
| *Lepiota echinella* + *L. fuscovinacea* | 0.000 | 0.000 | 0.000 | 0.000 | 0.008 | 0.069 | 0.000 | 0.009 | 0.000 | 0.000 | 0.000 | 0.000 | Agaricales | 99-100 | S |
| Leucosporidiales | 0.000 | 0.000 | 0.000 | 0.028 | 0.054 | 0.000 | 0.000 | 0.003 | 0.000 | 0.01 | 0.000 | 0.000 |  | 100 | S |
| *Leucosporidiella creatinivora* | 0.000 | 0.000 | 0.000 | 0.007 | 0.000 | 0.000 | 0.000 | 0.001 | 0.000 | 0.000 | 0.000 | 0.008 | Leucosporidiales | 99 | S |
| *Leucosporidium* sp. + *L. drummii*  + *L. fasciculatum* + *L. fellii* + *L. golubevii* | 0.000 | 0.011 | 0.005 | 0.009 | 0.039 | 0.006 | 0.274 | 0.028 | 0.355 | 0.03 | 0.000 | 0.185 | Leucosporidiales |  | S |
| *Luellia* sp. | 0.002 | 0.000 | 0.000 | 0.000 | 0.000 | 0.026 | 0.000 | 0.001 | 0.102 | 0.014 | 0.000 | 0.000 | Trechisporales | 99 | S |
| *Lycoperdon nigrescens* + *L. perlatum*  *L. pratense* + *L. subumbrinum* | 0.011 | 0.000 | 0.000 | 0.043 | 0.018 | 0.008 | 0.000 | 0.028 | 0.000 | 0.000 | 0.000 | 0.000 | Agaricales | 97-100 | S |
| *Malassezia* sp. + *M. cuniculi* + *M. globosa*  + ***M. restricta*** + *M. sympodialis* | 0.000 | 0.002 | 11.043 | 0.001 | 0.005 | 0.004 | 0.433 | 0.003 | 0.285 | 0.003 | 0.009 | 0.004 | Incertae Sedis | 98-100 | P |
| *Mastigobasidium* sp. | 0.000 | 0.000 | 0.000 | 0.001 | 0.015 | 0.009 | 0.000 | 0.000 | 0.000 | 0.000 | 0.000 | 0.000 | Leucosporidiales | 99 | S |
| *Melanogaster ambiguus* | 0.000 | 0.000 | 0.000 | 0.000 | 0.000 | 0.002 | 0.000 | 0.001 | 0.000 | 0.000 | 0.000 | 0.000 | Boletales | 99 | M |
| Meruliaceae | 0.000 | 0.000 | 0.000 | 0.000 | 0.000 | 0.000 | 0.000 | 0.003 | 0.000 | 0.000 | 0.000 | 0.000 | Polyporales | 100 |  |
| Microbotryaceae | 0.000 | 0.019 | 0.000 | 0.000 | 0.002 | 0.001 | 0.000 | 0.001 | 0.000 | 0.000 | 0.000 | 0.000 | Microbotryales | 99 |  |
| Microbotryomycetes | 0.022 | 0.266 | 0.000 | 0.029 | 0.026 | 0.036 | 0.000 | 0.022 | 0.343 | 0.035 | 0.000 | 0.007 |  | 100 |  |
| *Microbotryum kuehneanum* | 0.000 | 0.000 | 0.000 | 0.017 | 0.000 | 0.000 | 0.000 | 0.000 | 0.000 | 0.000 | 0.000 | 0.006 | Microbotryales | 99 | P |
| *Microstroma album* | 0.000 | 0.000 | 0.000 | 0.000 | 0.000 | 0.000 | 0.000 | 0.000 | 0.000 | 0.000 | 0.002 | 0.000 | Microstromatales | 99 | P |
| *Minimedusa polyspora* | 0.341 | 0.095 | 0.000 | 0.000 | 0.000 | 0.009 | 0.000 | 0.023 | 0.000 | 0.000 | 0.000 | 0.000 | Cantharellales | 99 | A |
| *Mrakiella aquatica* | 0.000 | 0.000 | 0.000 | 0.000 | 0.000 | 0.000 | 0.000 | 0.005 | 0.000 | 0.000 | 0.000 | 0.003 | Cystofilobasidiales | 99 | S |
| *Mucronella* sp. | 0.039 | 0.037 | 0.000 | 0.02 | 0.002 | 0.000 | 0.000 | 0.018 | 0.000 | 0.000 | 0.000 | 0.000 | Agaricales | 99 | S |
| *Mycena* sp + *M. cinerella* + *M. pearsoniana*  + *M. sanguinolenta* + *M. strobilinoidea*  + *M. zephirus* | 0.006 | 0.000 | 0.000 | 0.163 | 0.884 | 0.011 | 0.302 | 0.028 | 0.378 | 0.656 | 0.002 | 0.212 | Agaricales | 98-100 | S |
| Mycenaceae | 0.002 | 0.000 | 0.000 | 0.000 | 0.000 | 0.003 | 0.000 | 0.010 | 0.000 | 0.005 | 0.000 | 0.000 | Agaricales | 100 | S |
| *Naganishia cerealis* + *N. diffluens* | 0.03 | 0.205 | 0.000 | 0.051 | 0.016 | 0.006 | 0.000 | 0.008 | 0.000 | 0.002 | 0.012 | 0.000 | Filobasidiales | 99-100 | S |
| *Naucoria escharioides* | 0.000 | 0.000 | 0.000 | 0.000 | 0.000 | 0.000 | 0.004 | 0.000 | 0.000 | 0.000 | 0.000 | 0.000 | Agaricales | 99 | M |
| *Nidulariopsis iowensis* | 0.000 | 0.007 | 0.000 | 0.000 | 0.012 | 0.003 | 0.000 | 0.005 | 0.071 | 0.000 | 0.012 | 0.011 | Geastrales | 99 | S |
| *Oberwinklerozyma silvestris* + *O. straminea* | 0.000 | 0.002 | 0.000 | 0.000 | 0.000 | 0.003 | 0.000 | 0.003 | 0.000 | 0.000 | 0.000 | 0.001 | Incertae Sedis | 99-100 | A |
| *Occultifur externus* | 0.000 | 0.000 | 0.000 | 0.000 | 0.002 | 0.002 | 0.000 | 0.000 | 0.000 | 0.000 | 0.000 | 0.000 | Cystobasidiales | 98 |  |
| *Omphalotus flagelliformis* | 0.000 | 0.022 | 0.000 | 0.000 | 0.000 | 0.000 | 0.000 | 0.000 | 0.000 | 0.000 | 0.000 | 0.000 | Agaricales | 99 | S |
| *Paralepista flaccida* | 0.005 | 0.000 | 0.000 | 0.000 | 0.000 | 0.002 | 0.000 | 0.006 | 0.000 | 0.000 | 0.000 | 0.000 | Agaricales | 99 | S |
| *Paulisebacina allantoidea* | 0.000 | 0.000 | 0.000 | 0.353 | 0.000 | 0.000 | 0.000 | 0.000 | 0.000 | 0.000 | 0.000 | 0.008 | Sebacinales | 99 | M |
| *Peniophora aurantiaca* | 0.000 | 0.000 | 0.000 | 0.000 | 0.000 | 0.000 | 0.000 | 0.001 | 0.000 | 0.000 | 0.000 | 0.000 | Russulales | 99 | S |
| *Peniophorella pallida* + *P. praetermissa* | 0.003 | 0.002 | 0.000 | 0.018 | 0.000 | 0.007 | 0.000 | 0.027 | 0.000 | 0.000 | 0.000 | 0.000 | Hymenochaetales | 99-100 | S |
| *Phallus impudicus* | 0.007 | 0.004 | 0.000 | 0.024 | 0.021 | 0.017 | 0.000 | 0.032 | 0.000 | 0.000 | 0.000 | 0.000 | Phallales | 97 | S |
| *Phellinus castanopsidis* + *P.crustosus* | 0.013 | 0.007 | 0.000 | 0.001 | 0.000 | 0.028 | 0.000 | 0.058 | 0.000 | 0.000 | 0.000 | 0.030 | Hymenochaetales | 99-100 | P |
| *Phellodon atratus* | 0.495 | 0.002 | 0.000 | 0.000 | 0.000 | 0.004 | 0.000 | 0.018 | 0.000 | 0.000 | 0.000 | 0.000 | Thelephorales | 99 | M |
| *Phlebiella* sp. | 0.019 | 0.015 | 0.000 | 0.000 | 0.000 | 0.018 | 0.000 | 0.079 | 0.000 | 0.000 | 0.000 | 0.000 | Polyporales | 99 | S |
| *Phloeomana speirea* | 0.000 | 0.000 | 0.000 | 0.000 | 0.000 | 0.000 | 0.000 | 0.000 | 0.003 | 0.000 | 0.002 | 0.001 | Incertae Sedis | 99 | S |
| *Pholiota highlandensis* | 0.000 | 0.000 | 0.000 | 0.000 | 0.000 | 0.000 | 0.000 | 0.001 | 0.000 | 0.000 | 0.000 | 0.000 | Agaricales | 99 | S |
| *Phylloporus scabripes* | 0.001 | 0.000 | 0.000 | 0.000 | 0.000 | 0.005 | 0.000 | 0.006 | 0.000 | 0.000 | 0.000 | 0.000 | Boletales | 98 | M |
| *Phyllozyma subbrunnea* | 0.000 | 0.000 | 0.000 | 0.000 | 0.000 | 0.000 | 0.000 | 0.000 | 0.000 | 0.000 | 0.000 | 0.024 | Spiculogloeales | 99 |  |
| *Piloderma* sp. | 0.038 | 0.019 | 0.000 | 0.000 | 0.023 | 4.514 | 0.000 | 0.336 | 1.237 | 0.000 | 0.000 | 0.000 | Atheliales | 99 | S |
| *Piskurozyma* sp. | 0.000 | 0.000 | 0.000 | 0.000 | 0.000 | 0.000 | 0.000 | 0.000 | 0.111 | 0.000 | 0.000 | 0.017 | Filobasidiales | 99 | S |
| *Platygloea disciformis* | 0.013 | 0.000 | 0.000 | 0.000 | 0.000 | 0.000 | 0.000 | 0.000 | 0.000 | 0.000 | 0.000 | 0.000 | Platygloeales | 99 | S |
| *Pleurotus* sp. | 0.000 | 0.000 | 0.000 | 0.000 | 0.000 | 0.000 | 0.000 | 0.001 | 0.000 | 0.000 | 0.000 | 0.000 | Agaricales | 99 | S |
| *Pluteus atropungens* | 0.000 | 0.000 | 0.000 | 0.000 | 0.013 | 0.000 | 0.000 | 0.000 | 0.000 | 0.000 | 0.000 | 0.000 | Agaricales | 99 | S |
| *Podoscypha petalodes* | 0.000 | 0.019 | 0.000 | 0.000 | 0.000 | 0.000 | 0.000 | 0.000 | 0.000 | 0.000 | 0.000 | 0.000 | Polyporales | 98 | S |
| Polyporales | 0.162 | 0.153 | 0.000 | 0.05 | 0.038 | 0.008 | 0.000 | 0.014 | 0.000 | 0.000 | 0.000 | 0.023 |  | 100 |  |
| *Psathyrella sacchariolens* + *P. tenuicula* | 0.000 | 0.022 | 0.000 | 0.000 | 0.000 | 0.000 | 0.000 | 0.004 | 0.000 | 0.000 | 0.000 | 0.000 | Agaricales | 99-100 | S |
| *Pseudoinonotus crustosus* | 0.000 | 0.000 | 0.000 | 0.000 | 0.000 | 0.000 | 0.000 | 0.042 | 0.000 | 0.000 | 0.000 | 0.000 | Hymenochaetales | 99 | S |
| *Pseudotomentella* sp. + *P. mucidula* | 0.000 | 0.000 | 0.000 | 0.000 | 0.014 | 0.015 | 0.000 | 0.001 | 0.000 | 0.000 | 0.000 | 0.000 | Thelephorales | 99-100 | S |
| *Pseudotremella moriformis* | 0.000 | 0.000 | 0.000 | 0.000 | 0.000 | 0.000 | 0.000 | 0.000 | 0.093 | 0.000 | 0.000 | 0.000 | Tremellales | 99 | S |
| *Pterula* sp. | 0.000 | 0.000 | 0.000 | 0.000 | 0.000 | 0.000 | 0.000 | 0.000 | 0.000 | 0.000 | 0.842 | 0.000 | Agaricales | 99 | S |
| *Puccinia recondita* + *P. striiformis* | 0.000 | 0.000 | 0.000 | 0.000 | 0.038 | 0.003 | 0.000 | 0.015 | 0.000 | 0.000 | 0.000 | 0.000 | Pucciniales | 99-100 | M |
| Pucciniaceae | 0.000 | 0.000 | 0.000 | 0.004 | 0.165 | 0.000 | 0.000 | 0.000 | 0.000 | 0.000 | 0.000 | 0.000 | Pucciniales | 100 | M |
| *Quambalaria cyanescens* | 0.000 | 0.000 | 0.000 | 0.000 | 0.021 | 0.000 | 0.000 | 0.000 | 0.000 | 0.000 | 0.000 | 0.000 | Microstromatales | 99 | M |
| *Ramariopsis* sp. + *R. crocea* + *R. flavescens* | 0.022 | 0.007 | 0.000 | 0.000 | 0.000 | 0.022 | 0.000 | 0.083 | 0.000 | 0.000 | 0.000 | 0.000 | Agaricales | 98-100 | S |
| *Resinicium bicolor* | 0.000 | 0.000 | 0.000 | 0.000 | 0.000 | 0.000 | 0.000 | 0.000 | 0.000 | 0.06 | 0.000 | 0.000 | Hymenochaetales | 99 | P |
| *Rhizopogon graveolens* + ***R. mohelnensis*** | 0.000 | 0.000 | 0.000 | 0.088 | 0.000 | 0.003 | 0.000 | 0.011 | 0.000 | 0.000 | 0.000 | 0.000 | Boletales | 99-100 | M |
| *Rhodocollybia butyracea* | 0.000 | 0.000 | 0.000 | 0.000 | 0.000 | 0.001 | 0.000 | 0.000 | 0.000 | 0.000 | 0.000 | 0.000 | Agaricales | 99 | S |
| *Rhodotorula* sp. + *R. fragaria* + *R. glutinis* + ***R. toruloides*** | 0.014 | 0.013 | 0.000 | 0.000 | 0.01 | 0.002 | 0.000 | 0.128 | 0.000 | 0.000 | 0.000 | 0.004 | Sporidiobolales | 98-100 | A |
| *Rigidoporus sanguinolentus* | 0.000 | 0.000 | 0.000 | 0.013 | 0.005 | 0.000 | 0.000 | 0.000 | 0.003 | 1.053 | 0.000 | 0.666 | Polyporales | 99 | S |
| *Ripartites metrodii* | 0.000 | 0.000 | 0.000 | 0.000 | 0.000 | 0.000 | 0.000 | 0.003 | 0.000 | 0.000 | 0.000 | 0.000 | Agaricales | 99 | S |
| *Russula* sp. + *R.amethystina* + *R. badia*  + *R. chloroides* + *R. firmula* + *R. fragilis* + ***R. ionochlora*** + *R. nigricans* + *R. ochroleuca* | 0.424 | 0.238 | 0.000 | 0.007 | 0.424 | 14.618 | 0.483 | 2.229 | 2.261 | 0.000 | 0.002 | 0.046 | Russulales | 97-100 | M |
| + *R. paludosa* + *R. puellaris* + *R. sapinea*  + ***R. veternosa*** |  |  |  |  |  |  |  |  |  |  |  |  |  |  |  |
| Russulaceae | 0.007 | 0.033 | 0.005 | 0.000 | 0.008 | 0.133 | 0.000 | 0.018 | 0.031 | 0.000 | 0.000 | 0.000 | Russulales | 100 | M |
| Russulales | 0.000 | 0.000 | 0.000 | 0.000 | 0.000 | 0.000 | 0.000 | 0.001 | 0.000 | 0.000 | 0.000 | 0.000 |  | 100 | M |
| *Saitozyma podzolica* | 0.002 | 0.002 | 0.000 | 0.000 | 0.000 | 0.000 | 0.004 | 0.003 | 0.000 | 0.000 | 0.000 | 0.000 | Tremellales | 99 | A |
| *Schizopora paradoxa* | 0.000 | 0.000 | 0.000 | 0.000 | 0.000 | 0.000 | 0.000 | 0.000 | 0.000 | 0.019 | 0.000 | 0.000 | Hymenochaetales | 99 | S |
| *Scleroderma areolatum* | 0.007 | 0.000 | 0.000 | 0.000 | 0.000 | 0.000 | 0.000 | 0.001 | 0.164 | 0.000 | 0.000 | 0.000 | Boletales | 99 | M |
| *Scytinopogon* sp. | 0.000 | 0.000 | 0.000 | 0.000 | 0.000 | 0.000 | 0.000 | 0.000 | 0.000 | 0.017 | 0.000 | 0.000 | Trechisporales | 99 | L |
| *Sebacina* sp. | 0.000 | 0.000 | 0.000 | 0.000 | 0.093 | 0.000 | 0.000 | 0.000 | 0.000 | 0.079 | 0.000 | 0.000 | Auriculariales | 99 | S |
| Sebacinaceae | 0.003 | 0.000 | 0.000 | 0.013 | 0.004 | 0.037 | 0.000 | 0.022 | 0.000 | 0.896 | 0.000 | 0.000 | Sebacinales | 100 |  |
| Sebacinales | 0.017 | 0.033 | 0.000 | 0.035 | 0.008 | 0.001 | 0.699 | 0.032 | 0.000 | 1.943 | 0.100 | 0.014 |  | 100 |  |
| *Sirobasidium brefeldianum* | 0.000 | 0.000 | 0.000 | 0.000 | 0.000 | 0.000 | 0.000 | 0.003 | 0.000 | 0.000 | 0.000 | 0.000 | Tremellales | 99 | S |
| *Sistotrema* sp. | 0.001 | 0.000 | 0.000 | 0.004 | 0.054 | 0.009 | 0.000 | 0.028 | 0.000 | 0.000 | 0.000 | 0.000 | Cantharellales | 99 | S |
| *Sistotremastrum guttuliferum* | 0.000 | 0.000 | 0.020 | 0.000 | 0.000 | 0.000 | 0.02 | 0.000 | 0.022 | 0.000 | 0.000 | 0.000 | Trechisporales | 99 | S |
| *Solicoccozyma fuscescens* + *S. phenolica*  + ***S. terrea*** + *S. terricola* | 1.297 | 1.525 | 0.000 | 0.257 | 0.518 | 0.072 | 1.443 | 0.386 | 0.000 | 0.016 | 0.035 | 0.011 | Filobasidiales | 98-100 | A |
| Sporidiobolales | 0.819 | 0.011 | 0.000 | 0.000 | 0.001 | 0.008 | 0.364 | 0.054 | 0.000 | 0.000 | 0.023 | 0.014 |  | 100 |  |
| *Sporobolomyces* sp. + *S. roseus* | 0.002 | 0.000 | 0.000 | 0.003 | 0.000 | 0.001 | 0.000 | 0.006 | 0.000 | 0.000 | 0.000 | 0.003 | Sporidiobolales | 99-100 |  |
| *Strobilomyces* sp. | 0.001 | 0.000 | 0.000 | 0.000 | 0.000 | 0.000 | 0.000 | 0.000 | 0.000 | 0.000 | 0.000 | 0.003 | Boletales | 98 |  |
| *Stereum sanguinolentum* | 0.000 | 0.000 | 0.000 | 0.000 | 0.000 | 0.000 | 0.000 | 0.000 | 0.439 | 0.000 | 0.000 | 0.000 | Russulales | 99 | P |
| *Subulicystidium perlongisporum* | 0.001 | 0.000 | 0.000 | 0.000 | 0.000 | 0.003 | 0.000 | 0.004 | 0.000 | 0.000 | 0.000 | 0.000 | Trechisporales | 99 | S |
| *Suillus granulatus* | 0.000 | 0.000 | 0.000 | 0.001 | 0.000 | 0.000 | 0.000 | 0.015 | 0.000 | 0.000 | 0.000 | 0.000 | Boletales | 99 | M |
| *Tapinella atrotomentosa* | 0.016 | 0.013 | 0.000 | 0.000 | 0.000 | 0.001 | 0.000 | 0.001 | 0.000 | 0.000 | 0.000 | 0.000 | Boletales | 99 | S |
| *Tephrocybe anthracophila* | 0.000 | 0.000 | 0.000 | 0.000 | 0.000 | 0.000 | 0.000 | 0.008 | 0.000 | 0.000 | 0.000 | 0.000 | Agaricales | 99 | S |
| *Thelephora palmata* | 0.000 | 0.000 | 0.000 | 0.028 | 0.025 | 0.000 | 0.000 | 0.000 | 0.000 | 0.000 | 0.000 | 0.000 | Thelephorales | 99 | M |
| Thelephoraceae | 0.286 | 0.102 | 5.235 | 0.009 | 0.037 | 0.300 | 0.016 | 10.241 | 0.000 | 28.271 | 1.493 | 0.238 | Thelephorales | 100 | M |
| *Tilletiopsis* sp. | 0.000 | 0.000 | 0.000 | 0.000 | 0.012 | 0.000 | 0.000 | 0.000 | 0.000 | 0.000 | 0.000 | 0.000 | Incertae Sedis | 99 | P |
| *Tomentella* sp. + *T. badia* | 0.295 | 0.145 | 0.000 | 0.001 | 0.017 | 0.004 | 0.000 | 0.033 | 0.000 | 0.000 | 0.000 | 0.000 | Thelephorales | 99-100 | M |
| *Trametes* | 0.000 | 0.015 | 0.000 | 0.000 | 0.000 | 0.001 | 0.000 | 0.003 | 0.000 | 0.000 | 0.000 | 0.000 | Polyporales | 100 | S |
| *Trechispora* sp. + *T. suaveolens*  + *T. hymenocystis* + *T. invisitata*  + *T. stevensonii* | 0.034 | 0.011 | 0.000 | 0.077 | 0.038 | 0.481 | 0.000 | 0.228 | 0.096 | 0.000 | 0.000 | 0.000 | Trechisporales | 97-100 | S |
| Trechisporales | 0.018 | 0.013 | 0.000 | 0.000 | 0.191 | 0.084 | 0.000 | 0.122 | 0.000 | 0.278 | 0.000 | 0.001 |  | 100 | S |
| Tremella sp. + T. globispora | 0.000 | 0.000 | 0.000 | 0.000 | 0.000 | 0.000 | 0.000 | 0.003 | 0.000 | 0.000 | 0.000 | 0.000 | Tremellales | 99-100 | S |
| Tremellales | 0.033 | 0.002 | 0.000 | 0.081 | 0.02 | 0.007 | 0.127 | 0.03 | 0.006 | 0.000 | 0.000 | 0.11 |  | 100 | S |
| *Tremellomycetes* sp. | 0.029 | 0.011 | 0.000 | 0.031 | 0.19 | 0.073 | 0.000 | 0.139 | 0.223 | 0.047 | 0.002 | 0.02 |  | 99 | S |
| *Tricholoma* sp. + ***T. fulvum*** + *T. saponaceum* | 0.032 | 0.024 | 0.000 | 0.033 | 0.065 | 4.795 | 0.000 | 0.251 | 0.000 | 0.000 | 0.000 | 0.000 | Agaricales | 98-100 | M |
| Tricholomataceae | 0.007 | 0.000 | 0.000 | 0.005 | 0.014 | 0.000 | 0.000 | 0.005 | 0.000 | 0.012 | 0.000 | 0.000 | Agaricales | 100 | M |
| *Trichosporon* sp. | 0.001 | 0.000 | 0.000 | 0.093 | 0.637 | 0.024 | 0.008 | 0.022 | 0.003 | 4.771 | 0.194 | 0.073 | Trichosporonales | 99 | A |
| Trichosporonaceae | 0.000 | 0.000 | 0.000 | 0.000 | 0.01 | 0.008 | 0.000 | 0.000 | 0.000 | 0.000 | 0.000 | 0.000 | Trichosporonales | 100 | A |
| *Tubulicrinis* sp. | 0.023 | 0.004 | 0.000 | 0.000 | 0.000 | 0.023 | 0.000 | 0.082 | 0.000 | 0.000 | 0.000 | 0.000 | Hymenochaetales | 98 | S |
| *Tulasnella* sp. | 0.000 | 0.000 | 0.000 | 0.000 | 0.000 | 0.003 | 0.000 | 0.006 | 0.000 | 0.000 | 0.000 | 0.008 | Cantharellales | 99 | S |
| Tulasnellaceae | 0.000 | 0.000 | 0.000 | 0.029 | 0.000 | 0.000 | 0.000 | 0.000 | 0.000 | 0.000 | 0.000 | 0.001 | Cantharellales | 100 | S |
| *Tylopilus felleus* | 0.000 | 0.000 | 0.000 | 0.000 | 0.000 | 0.000 | 0.000 | 0.003 | 0.000 | 0.000 | 0.000 | 0.000 | Boletales | 99 | M |
| *Tylospora* sp. + ***T. asterophora*** | 0.242 | 0.093 | 0.000 | 0.000 | 0.930 | 26.421 | 0.000 | 2.249 | 1.166 | 0.036 | 0.037 | 4.233 | Atheliales | 99-100 | M |
| *Uncobasidium* sp. | 0.000 | 0.000 | 0.000 | 0.000 | 0.000 | 0.000 | 0.000 | 0.000 | 0.179 | 0.000 | 0.000 | 0.000 | Polyporales | 99 | P |
| *Uromyces pisi-sativi* | 0.000 | 0.000 | 0.000 | 0.007 | 0.422 | 0.002 | 0.000 | 0.000 | 0.000 | 0.007 | 0.000 | 0.000 | Pucciniales | 99 | P |
| Ustilaginaceae | 0.000 | 0.000 | 0.000 | 0.000 | 0.004 | 0.001 | 0.000 | 0.001 | 0.000 | 0.000 | 0.000 | 0.000 | Ustilaginales | 100 | P |
| *Ustilentyloma graminis* | 0.000 | 0.004 | 0.000 | 0.001 | 0.002 | 0.000 | 0.000 | 0.000 | 0.000 | 0.000 | 0.000 | 0.003 | Microbotryales | 99 | A |
| *Vishniacozyma carnescens* + *V. globispora*  + *V. victoriae* | 0.006 | 0.004 | 0.000 | 0.009 | 0.000 | 0.001 | 0.014 | 0.014 | 0.099 | 0.000 | 0.000 | 0.230 | Tremellales | 98-100 | P |
| *Vonarxula javanica* | 0.000 | 0.000 | 0.000 | 0.000 | 0.001 | 0.000 | 0.000 | 0.000 | 0.000 | 0.000 | 0.000 | 0.000 | Incertae Sedis | 99 | A |
| *Vuilleminia comedens* | 0.000 | 0.000 | 0.000 | 0.000 | 0.001 | 0.000 | 0.000 | 0.000 | 0.000 | 0.000 | 0.000 | 0.000 | Corticiales | 99 | S |
| *Xenasmatella* sp. | 0.001 | 0.000 | 0.000 | 0.000 | 0.000 | 0.048 | 0.000 | 0.006 | 0.000 | 0.000 | 0.000 | 0.000 | Polyporales | 99 | P |
| *Xerocomellus chrysenteron* + ***X. pruinatus*** | 0.071 | 0.117 | 0.000 | 0.004 | 0.037 | 1.860 | 0.000 | 0.123 | 0.000 | 0.000 | 0.000 | 0.000 | Boletales | 99-100 | M |
| *Xylodon flaviporus* + *X. raduloides* | 0.000 | 0.000 | 0.000 | 0.000 | 0.000 | 0.000 | 0.000 | 0.001 | 0.087 | 0.000 | 0.000 | 0.000 | Hymenochaetales | 99-100 | S |
| **Frequency of Basidiomycota** | 8.738 | 7.184 | 16.575 | 8.765 | 9.422 | 72.591 | 7.706 | 27.661 | 30.169 | 48.978 | 24.151 | 24.083 |  |  |  |
| **Chytridiomycota** | | | | | | | | | | | | | | | |
| *Alphamyces chaetifer* | 0.001 | 0.000 | 0.000 | 0.000 | 0.000 | 0.000 | 0.000 | 0.001 | 0.000 | 0.000 | 0.000 | 0.000 | Rhizophydiales | 99 | P |
| *Betamyces americaemeridionalis* | 0.015 | 0.002 | 0.000 | 0.000 | 0.000 | 0.001 | 0.000 | 0.000 | 0.000 | 0.000 | 0.000 | 0.000 | Rhizophydiales | 99 | P |
| *Blastocladiella emersonii* | 0.000 | 0.000 | 0.000 | 0.000 | 0.000 | 0.001 | 0.000 | 0.004 | 0.000 | 0.000 | 0.000 | 0.000 | Blastocladiales | 99 | P |
| Chytridiomycetes | 0.000 | 0.000 | 0.000 | 0.001 | 0.000 | 0.000 | 0.000 | 0.004 | 0.000 | 0.000 | 0.000 | 0.000 |  | 100 | P |
| Chytridiomycota | 0.034 | 0.126 | 0.000 | 0.01 | 0.023 | 0.343 | 0.000 | 0.049 | 0.000 | 0.007 | 0.002 | 0.000 |  | 100 | P |
| *Gallinipes pseudodichotomus* | 0.000 | 0.000 | 0.000 | 0.000 | 0.000 | 0.000 | 0.000 | 0.003 | 0.000 | 0.000 | 0.000 | 0.000 | Spizellomycetales | 99 | P |
| *Lobulomyces* sp. | 0.001 | 0.000 | 0.000 | 0.000 | 0.000 | 0.002 | 0.000 | 0.003 | 0.000 | 0.000 | 0.000 | 0.000 | Lobulomycetales | 99 | P |
| Monoblepharidomycetes | 0.001 | 0.000 | 0.000 | 0.005 | 0.000 | 0.002 | 0.000 | 0.004 | 0.000 | 0.000 | 0.000 | 0.000 |  | 97 | P |
| Olpidiales | 0.000 | 0.000 | 0.000 | 0.029 | 0.000 | 0.000 | 0.000 | 0.000 | 0.000 | 0.000 | 0.000 | 0.000 |  | 100 | P |
| *Olpidium brassicae* | 0.048 | 0.108 | 0.000 | 0.000 | 0.000 | 0.003 | 0.000 | 0.004 | 0.000 | 0.000 | 0.000 | 0.000 | Olpidiales | 99 | P |
| *Operculomyces laminatus* | 0.110 | 0.000 | 0.000 | 0.000 | 0.000 | 0.001 | 0.000 | 0.000 | 0.000 | 0.000 | 0.000 | 0.000 | Rhizophydiales | 99 | P |
| *Paraphysoderma sedebokerense* | 0.000 | 0.115 | 0.000 | 0.000 | 0.000 | 0.000 | 0.000 | 0.003 | 0.000 | 0.000 | 0.000 | 0.000 | Physodermatales | 98 | P |
| *Phlyctochytrium planicorne* | 0.001 | 0.000 | 0.000 | 0.000 | 0.000 | 0.000 | 0.000 | 0.001 | 0.000 | 0.000 | 0.000 | 0.000 | Chytridiales | 99 | P |
| Powellomyces sp. | 0.000 | 0.000 | 0.000 | 0.000 | 0.000 | 0.000 | 0.000 | 0.005 | 0.000 | 0.000 | 0.000 | 0.000 | Spizellomycetales | 99 | P |
| *Rhizophlyctis rosea* | 0.038 | 0.000 | 0.000 | 0.000 | 0.000 | 0.000 | 0.000 | 0.000 | 0.000 | 0.000 | 0.000 | 0.000 | Rhizophlyctidales | 99 | P |
| Rhizophydiales | 0.000 | 0.000 | 0.000 | 0.000 | 0.000 | 0.000 | 0.000 | 0.000 | 0.000 | 0.000 | 0.000 | 0.003 |  | 100 | P |
| *Sonoraphlyctis ranzonii* | 0.000 | 0.000 | 0.000 | 0.000 | 0.000 | 0.000 | 0.000 | 0.000 | 0.000 | 0.000 | 0.021 | 0.000 | Rhizophlyctidales | 99 | P |
| *Spizellomyces lactosolyticus* | 0.000 | 0.000 | 0.000 | 0.000 | 0.149 | 0.000 | 0.000 | 0.000 | 0.000 | 0.000 | 0.000 | 0.000 | Spizellomycetales | 99 | P |
| **Frequency of Chytridiomycota** | 0.249 | 0.352 | 0.000 | 0.046 | 0.171 | 0.352 | 0.000 | 0.081 | 0.000 | 0.007 | 0.023 | 0.003 |  |  |  |
| **Glomeromycota** | | | | | | | | | | | | | | | |
| *Acaulospora* sp. + *A. lacunosa* | 0.001 | 0.000 | 0.000 | 0.003 | 0.103 | 0.001 | 0.000 | 0.028 | 0.000 | 0.000 | 0.000 | 0.000 | Diversisporales | 99-100 | M |
| Acaulosporaceae | 0.001 | 0.000 | 0.000 | 0.000 | 0.000 | 0.001 | 0.000 | 0.006 | 0.000 | 0.000 | 0.000 | 0.000 | Diversisporales | 100 | M |
| *Ambispora leptoticha* | 0.000 | 0.000 | 0.000 | 0.000 | 0.000 | 0.000 | 0.000 | 0.004 | 0.000 | 0.000 | 0.000 | 0.000 | Archaeosporales | 99 | M |
| *Archaeospora* sp. | 0.002 | 0.000 | 0.000 | 0.035 | 0.000 | 0.003 | 0.000 | 0.018 | 0.000 | 0.000 | 0.000 | 0.000 | Archaeosporales | 99 | M |
| Archaeosporaceae | 0.002 | 0.000 | 0.000 | 0.000 | 0.000 | 0.005 | 0.000 | 0.027 | 0.000 | 0.000 | 0.000 | 0.000 | Archaeosporales | 100 | M |
| Archaeosporales | 0.000 | 0.000 | 0.126 | 0.000 | 0.010 | 0.008 | 0.000 | 0.100 | 0.009 | 0.000 | 0.000 | 0.000 |  | 100 | M |
| *Cetraspora* sp. | 0.000 | 0.000 | 0.000 | 0.000 | 0.000 | 0.000 | 0.000 | 0.001 | 0.000 | 0.000 | 0.000 | 0.000 | Diversisporales | 99 | M |
| *Claroideoglomus luteum* | 0.000 | 0.000 | 0.000 | 0.000 | 0.000 | 0.000 | 0.000 | 0.000 | 0.241 | 0.000 | 0.000 | 0.000 | Glomerales | 99 | M |
| *Entrophospora* sp. | 0.000 | 0.000 | 0.000 | 0.000 | 0.000 | 0.000 | 0.000 | 0.000 | 0.000 | 0.000 | 0.000 | 0.000 | Diversisporales | 99 | M |
| Gigasporaceae | 0.000 | 0.000 | 0.000 | 0.000 | 0.000 | 0.000 | 0.000 | 0.009 | 0.000 | 0.000 | 0.000 | 0.000 | Diversisporales | 98 | M |
| Glomeraceae | 0.006 | 0.000 | 0.000 | 0.024 | 0.055 | 0.007 | 0.000 | 0.048 | 0.000 | 0.000 | 0.000 | 0.054 | Glomerales | 100 | M |
| Glomerales | 0.000 | 0.000 | 0.000 | 0.000 | 0.000 | 0.000 | 0.000 | 0.015 | 0.000 | 0.000 | 0.000 | 0.000 |  | 100 | M |
| Glomeromycetes | 0.002 | 0.045 | 0.000 | 0.021 | 0.012 | 0.002 | 0.000 | 0.004 | 0.000 | 0.000 | 0.000 | 0.000 |  | 97 | M |
| Glomeromycota | 0.001 | 0.000 | 0.000 | 0.003 | 0.000 | 0.001 | 0.000 | 0.006 | 0.000 | 0.000 | 0.000 | 0.004 |  | 100 | M |
| Paraglomerales | 0.000 | 0.002 | 0.000 | 0.000 | 0.000 | 0.001 | 0.000 | 0.004 | 0.000 | 0.000 | 0.000 | 0.000 |  | 100 | M |
| *Paraglomus* sp. | 0.002 | 0.000 | 0.000 | 0.000 | 0.000 | 0.006 | 0.000 | 0.010 | 0.000 | 0.000 | 0.000 | 0.000 | Paraglomerales | 99 | M |
| *Scutellospora* sp. + *S. alterata* | 0.000 | 0.000 | 0.000 | 0.012 | 0.000 | 0.000 | 0.000 | 0.007 | 0.000 | 0.000 | 0.000 | 0.000 | Diversisporales | 99-100 | M |
| Rozellomycota | 0.133 | 2.768 | 0.005 | 1.400 | 2.219 | 0.221 | 1.247 | 0.973 | 1.432 | 0.211 | 0.648 | 0.096 |  | 100 |  |
| **Frequency of Glomeromycota** | 0.151 | 2.814 | 0.131 | 1.498 | 2.399 | 0.254 | 1.247 | 0.131 | 1.682 | 0.211 | 0.648 | 0.154 |  |  |  |
| **Zygomycota** | | | | | | | | | | | | | | | |
| *Absidia* sp. + *A. caerulea* + *A. cylindrospora*  + *A. glauca* | 0.057 | 0.004 | 0.000 | 0.055 | 0.024 | 0.043 | 0.000 | 0.117 | 0.000 | 0.000 | 0.033 | 0.000 | Mucorales | 98-100 | S |
| Basidiobolaceae | 0.000 | 0.000 | 0.000 | 0.054 | 0.000 | 0.000 | 0.000 | 0.000 | 0.000 | 0.000 | 0.000 | 0.000 | Basidiobolales | 100 | S |
| Basidiobolales | 0.050 | 0.000 | 0.000 | 0.000 | 0.000 | 0.000 | 0.000 | 0.001 | 0.000 | 0.000 | 0.000 | 0.000 |  | 100 | S |
| *Basidiobolus ranarum* | 0.053 | 0.061 | 0.000 | 1.121 | 7.345 | 0.083 | 0.000 | 0.223 | 0.000 | 0.000 | 0.000 | 0.028 | Basidiobolales | 99 | S |
| Endogonales | 0.000 | 0.000 | 0.000 | 0.000 | 0.000 | 0.009 | 0.000 | 0.000 | 0.000 | 0.000 | 0.000 | 0.000 |  | 100 | S |
| *Endogone* sp. | 0.000 | 0.000 | 0.000 | 0.001 | 0.003 | 0.000 | 0.004 | 0.011 | 0.003 | 0.060 | 0.042 | 0.220 | Endogonales | 99 | S |
| *Halteromyces radiatus* | 0.000 | 0.000 | 0.000 | 0.016 | 0.000 | 0.001 | 0.000 | 0.003 | 0.000 | 0.000 | 0.000 | 0.000 | Mucorales | 99 | S |
| *Jimgerdemannia lactiflua* | 0.000 | 0.000 | 0.000 | 0.000 | 0.000 | 0.000 | 0.000 | 0.010 | 0.000 | 0.019 | 0.070 | 0.062 | Endogonales | 98 | S |
| *Mortierella* sp. | 10.311 | 6.511 | 0.000 | 10.359 | 13.572 | 2.728 | 1.639 | 4.323 | 0.812 | 0.300 | 0.322 | 0.248 | Mortierellales | 99 | S |
| Mortierellaceae | 0.015 | 0.009 | 0.000 | 0.998 | 0.790 | 0.158 | 0.556 | 0.087 | 0.133 | 0.088 | 0.040 | 0.118 |  | 100 | S |
| Mortierellales | 0.105 | 0.158 | 0.000 | 1.598 | 1.116 | 0.216 | 0.000 | 0.217 | 0.000 | 0.031 | 0.016 | 0.066 |  | 97 | S |
| *Mucor* sp. | 0.059 | 0.026 | 0.000 | 0.110 | 0.028 | 0.000 | 0.069 | 0.005 | 0.000 | 0.000 | 0.063 | 0.000 | Mucorales | 99 | S |
| Mucorales | 0.000 | 0.002 | 0.000 | 0.000 | 0.000 | 0.002 | 0.000 | 0.009 | 0.000 | 0.000 | 0.000 | 0.000 |  | 100 | S |
| *Pandora kondoiensis* | 0.000 | 0.000 | 0.000 | 0.000 | 0.000 | 0.004 | 0.000 | 0.008 | 0.000 | 0.000 | 0.000 | 0.000 | Entomophthorales | 100 | S |
| *Piptocephalis graefenhanii* + *P. tieghemiana* | 0.013 | 0.004 | 0.000 | 0.059 | 0.000 | 0.000 | 0.000 | 0.001 | 0.000 | 0.000 | 0.000 | 0.000 | Zoopagales | 99-100 | S |
| *Ramicandelaber* sp. | 0.025 | 0.000 | 0.000 | 0.001 | 0.000 | 0.001 | 0.000 | 0.003 | 0.000 | 0.000 | 0.000 | 0.000 | Ramicandelaberales | 99 | S |
| *Rhizopus arrhizus* | 0.000 | 0.043 | 0.000 | 0.000 | 0.000 | 0.001 | 0.000 | 0.000 | 0.000 | 0.000 | 0.000 | 0.000 | Mucorales | 99 | S |
| *Schizangiella* sp. + *S. serpentis* | 0.002 | 0.000 | 0.000 | 0.000 | 0.015 | 0.007 | 0.000 | 0.014 | 0.000 | 0.000 | 0.000 | 0.000 | Basidiobolales | 99-100 | S |
| *Smittium morbosum* | 0.000 | 0.000 | 0.000 | 0.000 | 0.000 | 0.002 | 0.000 | 0.005 | 0.000 | 0.000 | 0.000 | 0.000 | Harpellales | 99 | S |
| *Spiromyces aspiralis* | 0.000 | 0.000 | 0.000 | 0.000 | 0.000 | 0.001 | 0.000 | 0.000 | 0.000 | 0.000 | 0.000 | 0.000 | Kickxellales | 98 | S |
| *Sporodiniella umbellata* | 0.000 | 0.000 | 0.000 | 0.003 | 0.000 | 0.001 | 0.000 | 0.000 | 0.000 | 0.000 | 0.000 | 0.000 | Mucorales | 99 | S |
| *Syncephalis* sp. | 0.592 | 0.143 | 0.000 | 0.168 | 0.030 | 0.004 | 0.000 | 0.047 | 0.000 | 0.014 | 0.002 | 0.004 | Zoopagales | 99 | S |
| *Umbelopsis* sp. | 0.056 | 0.033 | 0.000 | 0.396 | 0.139 | 0.283 | 0.004 | 0.558 | 0.776 | 0.012 | 0.063 | 0.023 | Umbelopsidales | 99 | S |
| *Utharomyces epallocaulus* | 0.000 | 0.000 | 0.000 | 0.000 | 0.000 | 0.009 | 0.000 | 0.015 | 0.000 | 0.000 | 0.000 | 0.000 | Mucorales | 98 | S |
| **Frequency of Zygomycota** | 11.339 | 6.994 | 0.000 | 14.94 | 23.063 | 3.553 | 2.272 | 5.657 | 1.724 | 0.524 | 0.651 | 0.769 |  |  |  |
| Uncultured Fungi | 31.692 | 26.915 | 60.485 | 19.882 | 22.037 | 7.004 | 7.840 | 12.200 | 21.159 | 1.992 | 6.547 | 3.073 |  |  |  |
| no sequence in database | 16.434 | 35.299 | 9.481 | 16.871 | 23.162 | 3.368 | 8.252 | 8.548 | 10.344 | 5.898 | 5.849 | 2.499 |  |  |  |
|  |  |  |  |  |  |  |  |  |  |  |  |  |  |  |  |
